# Metabolomic and chemometric profiling of crimson grapes during drying and its impact on antidiabetic activity

**DOI:** 10.1038/s41538-025-00509-5

**Published:** 2025-07-23

**Authors:** Doaa M. Abo-Atya, Doaa A. Ghareeb, Badr Aldahmash, Shaden A. M. Khalifa, Hesham R. El-Seedi, Eman Shawky, Dina S. Ghallab

**Affiliations:** 1 Department of Chemistry, Faculty of Science, Menoufia University, Shebin El-Kom, Egypt; 2 Bio‑Screening and Preclinical Trial Lab, Biochemistry Department, Faculty of Science, Alexandria University, Alexandria, Egypt; 3 Center of Excellence for Drug Preclinical Studies (CE-DPS), Pharmaceutical and Fermentation Industry Development Center, City of Scientific Research & Technological Applications (SRTA-city), New Borg El Arab, Egypt; 4 Research Projects unit, Pharos University, Alexandria, Egypt; 5 Department of Zoology, College of Science, King Saud University, P.O. Box 2455, Riyadh, 11451, Saudi Arabia; 6 Neurology and Psychiatry Department, Capio Saint Göran’s Hospital, Göransplan 1, 112 19, Stockholm, Sweden; 7 Department of Pharmacognosy, Faculty of Pharmacy, Alexandria University, Alexandria, Egypt

**Keywords:** Secondary metabolism, Metabolomics, Natural products

## Abstract

The present study investigates the metabolic profile and antidiabetic potential of Crimson Seedless grapes during the drying process using UPLC–QTOF-MS/MS and multivariate statistical analyses. A total of 50 metabolites were tentatively identified, including phenolic acids, flavonoids, stilbenes, anthocyanins, and lipids. Fresh grapes contain higher levels of glycosylated anthocyanins and stilbenes, while raisins exhibit an increased concentration of fatty acids and phosphatidylcholines. In vitro assays demonstrated potent *α*-amylase and *α*-glucosidase inhibitory activities of fresh grape extracts (IC_50_ = 157.5 ± 0.56 μg/mL and 91.89 ± 0.47 μg/mL, respectively), surpassing acarbose (positive control). Orthogonal projection to latent structures analysis correlated bioactive metabolites, identifying key compounds including dihydrokaempferol glucoside, quercetin-3-*O*-glucuronide, cyanidin-3-*O*-glucoside, protocatechuic acid glucoside, ergosterol, and bilobalide as potential contributors to α-amylase and α-glucosidase inhibitory activities, which may support antidiabetic potential. These findings suggest that fresh Crimson Seedless grapes possess superior antidiabetic potential, advocating their potential role as a functional food for diabetes management.

## Introduction

Grapes are worldwide-valued fruits increasingly consumed either fresh or in processed products including seed oil, wine, vinegar, jam, seed extract, juice, jelly, raisins, and vinegar^1,2^. An estimate of seventy species exists in the genus *Vitis*, with *Vitis vinifera* L. being the most predominant species extensively cultivated and studied^3,4^. *V. vinifera* is a deciduous perennial climbing vine that produces clusters of 15–300 grape berries and can reach 50 feet in height^5^. The exterior colors of berries’ skin range from green/yellow to dark blue colors based on *Vitis* species and varieties, as well as the amount and composition of anthocyanins^6^. In addition to skin color, grapes could be differentiated according to the presence or absence of seeds as seeded or seedless^5^.

Crimson Seedless is amongst the most prominent cultivars of *V. vinifera* table grape^7^. It was brought to Egypt by the “Horticultural Crops Research Laboratory of the U.S.” Department of Agriculture, California^8^. Today, it is widely cultivated on a vast scale in the newly reclaimed desert lands in Egypt^9^. It is a late-ripening variety with outer firm skin, juicy pulp, bright red berries, and a long shelf-life, making it available in various markets^10^. Concerning the traits of bunch and berry characteristics of various grape varieties, Crimson Seedless exhibited the lowest content of total sugars of 15.59% and the minimum reducing sugars (13.54%) compared to other varieties^11^.

Raisins are the edible dried product of fresh grape berries, extensively utilized worldwide due to their health-promoting constituents^12^. Grapes can be dried on the vine, in the open sun, in the shade, or mechanically to generate raisins^13^. Chemical or physical treatments are required in the case of the grapes' outer waxy cuticle to reduce skin resistance and enhance moisture diffusion^13^.

The process of drying grapes into raisins is a traditional preservation technique that extends the shelf life of the fruit while concentrating its nutrients and flavors. Drying significantly reduces the moisture content of fresh grapes, thereby inhibiting microbial growth and enzymatic degradation, making raisins more stable for long-term storage and transport^12^. This transformation is also economically advantageous, as dried fruits weigh less and require less storage space compared to fresh grapes. Additionally, the drying process enhances the bioavailability of certain phytochemicals, such as polyphenols, which contribute to the antioxidant properties of raisins^14^. However, it also leads to compositional changes, including a reduction in water-soluble vitamins and anthocyanins while increasing sugar concentration, which affects their nutritional and functional properties^15^.

Throughout raisin drying, they acquire a dark brown color due to the buildup of melanin brown-black pigments generated by the activities of polyphenol oxidase and non-enzymatic processes^16^. Raisins are thought to be the most consumed dried fruit, with consumption six times that of other dried fruits due to their widespread use in different meals including pastries, breads, cereals, and muffins^17^. Despite the misconception that raisins are harmful due to their high sugar content (60%)^18^, they are recommended as nutritious snacks with a low glycemic index owned to tartaric acid content and the existing sugar^17^. Additionally, raisins contribute to the enhancement of diet quality and appetite control^12^. Eating raisins has a significant impact on decreasing the chance of getting diabetes or heart diseases^19^. A previous report has revealed that the main compositional variation between grapes and raisins was the water content and the essential nutrients, noticeably not affected by the drying process^20^.

Crimson Seedless grapes serve as a prime store of health-promoting metabolites, including flavonoids, anthocyanins, reducing sugars, cardiac glycosides, quinones, tannins, sterols, triterpenes, and polyphenolic compounds^21–23^. Previous reports have demonstrated the potent antioxidant and anti-proliferative activities of Crimson Seedless grapes^22,23^. Raisins are rich in polyphenolics compared to other dried fruits, including prunes, apricots, and figs, making them powerful antioxidant agents. Compared to equal caloric carbohydrate snacks, raisins’ consumption lowers low-density lipoprotein cholesterol, blood glucose, and blood pressure contributing to a reduced risk of CVD^24,25^.

The current study integrated “Ultra-performance liquid chromatography coupled with quadrupole time of flight mass spectrometry (UPLC–QTOF-MS/MS)” with chemometric analysis for the first monitoring of the chemical patterns of Crimson Seedless grape over the course of the drying process. Next, the sample extracts, including fresh (untreated) and those collected at four distinctive drying stages, were screened for in vitro antidiabetic activity using *α*-amylase and *α*-glucosidase inhibitory assays. Coincidentally, OPLS was performed to rationally correlate the metabolite profiles with the prior investigated antidiabetic activity, identifying the possible bioactive compounds that synergistically mediate antidiabetic activity. To the best of our knowledge, this is the first comprehensive profiling of Crimson Seedless grape secondary metabolites over the course of the drying process.

## Results and discussion

### Tentative annotation of grape metabolites

A total of 50 metabolites were tentatively annotated from the different analyzed samples, including fresh and dehydrated grape extracts, using UPLC–QTOF-MS/MS analysis. Following the metabolomics standards initiative (MSI) guidelines, the characterization of metabolites in the current investigation was carried out through comparison of their retention times and tandem mass profiles (quasi-molecular ions and distinguishing fragmentation patterns at three collision energies of 10 eV, 20 eV and 40 eV) with spectral databases and relevant literature using the same experimental conditions.

These metabolites were categorized into several chemical classes comprising organic acids, phenolic acids, flavonoids, stilbenes, sugars, anthocyanins, terpenoids, coumarins, auxins, sesquiterpenoids, procyanidins, sterols, terpenoids, as well as lipids.

Supplementary Fig. 1 depicts representative base peak chromatograms (BPCs) of the analyzed grape samples, revealing the principal metabolite classes and their elution region. The tentatively annotated metabolites from grape samples were numbered based on their elution order. Further, their possible fragment ions, retention time, molecular formulas, and chemical classes were represented in Table 1. The chemical structures of the tentatively annotated phytochemicals acquired from different grape samples were drawn in Supplementary Fig. 2.

**Table 1:** List of tentatively annotated compounds acquired from different grape samples, including fresh and those collected at distinctive drying stages, using UPLC-QTOF-MS/MS analysis

No.	Rt (min.)	Annotated compound	*m/z* (adduct)	Molecular formula	Error (ppm)	MS/MS fragments	Chemical class	References
1.	1.028	Citric acid	191.1215 [M − H]^−^	C₆H₈O₇	7.8	111	Organic acids	^26^
2.	1.246	Protocatechuic acid glucoside	315.2625 [M − H]^−^	C_13_H_16_O_9_	7.9	153 and 108	Phenolic acids	^31^
3.	1.354	Caffeic acid	179.1611 [M − H]^−^	C_9_H_8_O_4_	6.1	135	Phenolic acids	^31^
4.	1.367	Dimethoxycinnamic acid	207.2120 [M − H]^−^	C_11_H_12_O_4_	9.6	163	Organic acids	^27^
5.	1.445	1-Caffeoyl-beta-*D*-glucose; caffeic acid-glucoside	341.3012 [M − H]^−^	C_15_H_18_O_9_	3.5	161 and 135	Phenolic acids	^41^
6.	1.476	Rosmarinic acid	359.3017 [M − H]^−^	C_18_H_16_O_8_	4.7	197, 182, and 122	Phenolic acids	^70^
7.	1.496	Dihydroferulic acid	195.2015 [M − H]^−^	C_10_H_12_O_4_	7.6	153	Phenolic acids	^70^
8.	1.584	Quercetin-3-*O*-glucuronide	477.4033 [M − H]^−^	C_21_H_18_O_13_	6.8	314, 301, 243, 179, 151, and 108	Flavonoids	^36,37^
9.	1.896	Esculin	341.2815 [M + H]^+^	C_15_H_16_O_9_	4.4	161 and 135	Coumarin glycosides	^71^
10.	2.042	Vinyl caffeate	205.1918 [M − H]^−^	C_11_H_10_O_4_	8.7	163	Phenolic acid esters	^72^
11.	2.117	Polydatin	390.4025 [M]^+^	C_20_H_22_O_8_	6.4	333, 279, and 228	Stilbene glycosides	^39^
12.	2.216	Caftaric acid	311.2310 [M − H]^−^	C_13_H_12_O_9_	3.2	217 and 149	Phenolic acids	^73^
13.	2.233	Uridine 5’-diphosphogalactose	565.3051 [M − H]^−^	C_15_H_24_N_2_O_17_P_2_	9.0	385, 323, 273, 241, 211, and 159	Pyrimidine nucleotide sugars	^74^
14.	2.320	Coutaric acid	295.2318 [M − H]^−^	C_13_H_12_O_8_	6.1	163 and 119	Phenolic acids	^32^
15.	2.334	Sinapinic acid	261.2109 [M − H + K]^−^	C_11_H_12_O_5_	4.0	181 and 119	Phenolic acids	^33^
16.	2.349	Indole-3-acetic acid	175.1816 [M]^−^	C_10_H_9_NO_2_	9.1	131	Auxins	^75^
17.	2.351	Kaempferol diglycoside	611.5020 [M + H]^+^	C_27_H_30_O_16_	3.27	341, 287, 227, 163, and 113	Flavonoids	^41^
18.	2.362	Vitisinol C	449.5011 [M − H + Na]^−^	C_27_H_24_O_5_	2.6	287, 269, 227, 209, 155, and 113	Stilbenes	^76^
19.	2.389	Dihydrokaempferol glucoside	449.4017 [M − H]^−^	C_21_H_22_O_11_	3.8	287, 269, 227, 209, and 155	Flavonoids	^41^
20.	2.390	Cyanidin-3-*O*-glucoside	449.4006 [M]^+^	C_21_H_21_O_11_^+^	1.3	287, 269, 227, 209, 207, and 155	Anthocyanins	^41,77^
21.	2.420	4-Hydroxybenzoyl glucose	299.2616 [M − H]^−^	C_13_H_16_O_8_	5.3	239, 179, and 137	Sugars	^78^
22.	2.467	Abscisic acid	263.3213 [M − H]^−^	C_15_H_20_O_4_	4.9	204, 165, 153, and 135	Sesquiterpenoids	^79^
23.	2.498	Spiraeoside	463.4022 [M − H]^−^	C_21_H_20_O_12_	4.7	300, 179, and 151	Flavonoids	^80^
24.	2.498	Quercetin 3-*O*-galactoside; hyperoside	463.4013 [M − H]^−^	C_21_H_20_O_12_	2.7	300, 179, and 151	Flavonoids	^41,81^
25.	2.499	Peonidin-3-*O*-glucoside	463.9001 [M]^−^	C_22_H_23_O_11_^+^	0.2	301 and 300	Anthocyanins	^41^
26.	2.715	Petunidin 3-(6”-acetylglucoside)	520.4035 [M − H]^-^	C_24_H_25_O_13_^+^	6.7	317, 249, 181, and 113	Anthocyanins	^82^
27.	4.724	Pentahydroxyflavone	303.2326 [M + H]^+^	C_15_H_10_O_7_	8.6	229, 165, 153, and 137	Flavonoids	^83^
28.	5.502	Myristic acid (Tetradecanoic acid)	249.3723 [M − H + Na]^−^	C_14_H_28_O_2_	10.0	155 and 113	Lipids (fatty acids)	^84^
29.	5.678	Viniferal	595.6019 [M − H + Na]^−^	C_35_H_26_O_8_	3.3	461, 415, 279, and 153	Stilbenes	^76^
30.	5.792	PC (16:1/0:0)	494.6026 [M + H]^+^	C_24_H_48_NO_7_P	5.3	311, 184, 125, and 104	Lipids (phosphatidylcholines)	GNPS^42^
31.	5.899	PC (18:2/0:0)	520.7039 [M + H]^+^	C_26_H_50_NO_7_P	7.5	337, 184, 125, and 104	Lipids (phosphatidylcholines)	GNPS^42^
32.	6.148	PC (18:3/0:0)	518.6025 [M + H]^+^	C_26_H_48_NO_7_P	4.8	335, 184, 125, and 104	Lipids (phosphatidylcholines)	GNPS^42^
33.	6.150	PC (0:0/16:0)	496.6022 [M + H]^+^	C_24_H_50_NO_7_P	4.4	313, 184, 125, and 104	Lipids (phosphatidylcholines)	GNPS^42^
34.	6.258	Hydroxytetracosanoic acid	384.6038 [M]^−^	C_24_H_48_O_3_	9.8	181 and 113	Lipids (fatty acids)	^84^
35.	6.373	Myristic acid, 2-(1-octadecenyloxy) ethyl ester	521.9035 [M − H]^−^	C_34_H_66_O_3_	6.7	452, 255, 181, and 113	Lipids (fatty acids)	^84^
36.	6.545	Amurensisin	461.4016 [M − H + Na]^−^	C_22_H_16_O_10_	3.6	279 and 181	Procyanidins	^85^
37.	6.923	1-Hexadecanoyl-sn-glycero-3-phospho-(1’-myo-inositol); PI (16:0/0:0)	571.6028 [M − H]^−^	C_25_H_49_O_12_P	4.9	391, 315, 255, 241, and 153	Lipids (phosphatidylinositols)	GNPS^86^
38.	7.736	Proanthocyanidin A	591.5012 [M − H]^−^	C_31_H_28_O_12_	2.0	523, 455, 351, 277, and 153	Flavonoids	^85^
39.	7.774	Viniferether A	523.5026 [M − H + K]^−^	C_29_H_26_O_7_	5.3	455	Stilbenes	^76^
40.	8.087	Ergosterol	433.6015 [M − H_2_O + Na]^+^	C_28_H_44_O	3.8	325, 311, 255, and 153	Sterols	^87^
41.	8.278	PE(18:2/17:0)	731.0054 [M + 2H]^+^	C_40_H_76_NO_8_P	7.4	337	Lipids (phosphatidylethanolamines)	^88^
42.	8.305	PE(16:0/18:2)	717.0057 [M + H]^+^	C_39_H_74_NO_8_P	7.9	478 and 323	Lipids (phosphatidylethanolamines)	^88^
43.	8.320	PE(18:2/15:1)	699.9051 [M + H]^+^	C_38_H_70_NO_8_P	7.3	305 and 225	Lipids (phosphatidylethanolamines)	^88^
44.	8.428	PE(18:2/22:6)	786.0027 [M − 2H]^−^	C_45_H_74_NO_8_P	3.1	557 and 291	Lipids (phosphatidylethanolamines)	^88^
45.	8.442	PE(14:0/18:3)	685.9017 [M]^+^	C_37_H_68_NO_8_P	2.5	291 and 140	Lipids (phosphatidylethanolamines)	^88^
46.	8.494	PE(18:2/PGE1)	816.0015 [M + 2H]^+^	C_43_H_76_NO_11_P	1.8	587, 599, 293, and 109	Lipids (phosphatidylethanolamines)	^88^
47.	8.507	PE(18:1/18:1)	745.0058 [M + H]^+^	C_41_H_78_NO_8_P	7.8	478 and 351	Lipids (phosphatidylethanolamines)	^88^
48.	8.577	7,10-dihydroxy-octadecadienoic acid (7,10-DiHODE)	311.4028 [M − H]^−^	C_18_H_32_O_4_	8.9	183	Lipids (fatty acids)	^89^
49.	8.791	Bilobalide	325.3028 [M − H]^−^	C_15_H_18_O_8_	8.6	261, 183, 119, and 103	Terpenoids	GNPS^41^
50.	8.989	PE (18:2 (10, 12) + = O (9)/22:1 (13))	813.1068 [M + H]^+^	C_45_H_82_NO_9_P	8.4	585, 557, and 291	Lipids (phosphatidylethanolamines)	^88^

In the present study, only two organic acids (**1** and **4**) were detected in negative ionization mode. Compound (**1**), with a precursor ion [M − H]^−^ *m/z* of 191.1215, was tentatively annotated as citric acid and generated an intense fragment signal at *m/z* 111 corresponding to [M − H − CO_2_ − 2H_2_O]^−^, further supported by previous data^26^. Considering peak **4** at *m*/*z* 207.2120, it was tentatively identified as dimethoxy cinnamic acid and showed a fragment ion at *m*/*z* 163 through the loss of a CO_2_ unit (44 Da)^27^ (Table 1).

Grape phenolics differ in chemical structures and thus in their pharmacological activities. They could be primarily categorized into two main classes: flavonoids and non-flavonoids. The majority of these phenolic compounds exist as glycosylated derivatives in plants and foods and undergo enzymatic changes in the stomach before intestinal absorption^28^. Phenolic acids, flavonoids, stilbenes, and anthocyanins are examples of phenolic compounds detected in different grape samples.

Phenolic acids are essential plant metabolites existing in grapes, as well as their wines and juices^29^. Phenolic acids account for the sensory properties, chemical stability, and several health benefits of grape-derived products^29^.

In the present investigation, a total of nine peaks representing phenolic acids and their derivatives (**2**, **3**, **5**, **6**, **7**, **10**, **12**, **14**, and **15**) were recorded with better response in negative polarity mode. All demonstrated a relatively comparable fragmentation pattern manifested by the loss of the carboxyl group (CO_2_) unit (44 Da)^30^. Compound (**2**), generated a deprotonated precursor ion [M − H]^−^ *m/z* of 315.2625 and was tentatively annotated as protocatechuic acid glucoside. Two fragment ion signals were observed at *m/z* 153 and 108, attributed to successive loss of glucose moiety (162 Da) and CO_2_ unit (44 Da), respectively^31^.

The suggested candidate for peak (**3**) was caffeic acid based on the precursor ion at *m/z* 179.1611. MS^2^ confirmed the tentative identification and revealed a fragment at *m/z* 135, evidently explained by the loss of CO_2_^31^. Within the same context, compound (**14**) was tentatively characterized as coutaric acid with the main parent ion of *m/z* 295.2318, accompanied by two product ion peaks at *m/z* 163 and 119 after the sequential loss of the hydroxy succinic acid moiety (134 Da) and carboxyl group (44 Da), respectively^32^. The proposed compound for peak (**15**) was sinapinic acid with molecular ion signal [M − H + K] ^−^ *m/z* 261.2109. The highest MS^2^ fragments detected were at *m/z* 181 and 119, caused by coherent loss of carboxyl group (44 Da) and two methoxy groups (62 Da), respectively^33^ (Table 1).

Grape berries’ complex phytochemistry exerts exceptional medicinal and health-promoting properties^34^. Among them, polyphenolic compounds, particularly flavonoids, are the main candidates responsible for grape antimicrobial, neuroprotective, antioxidant, cardioprotective, anti-cancer, antiviral, anti-inflammatory, and hepatoprotective effects^35^.

Seven flavonoids (**8**, **17**, **19**, **23**, **24**, **27**, and **38**) were clearly profiled in negative ionization mode. These metabolites were tentatively identified as quercetin-3-*O*-glucuronide, kaempferol diglycoside, dihydrokaempferol glucoside, spiraeoside, hyperoside, pentahydroxyflavone, and proanthocyanidin A as represented in Table 1. Compound (8) at *R*t = 1.584 min offered a [M − H]^−^ at *m/z* 477.4033 accompanied by successive fragment ion peaks at *m/z* 314, 301, 243, 179, 151, and 108, and it was tentatively identified as quercetin-3-*O*-glucuronide. The loss of glucuronide moiety [M − H − 176]^−^ generated a product ion at *m/z* 301 corresponding to quercetin. The fragment ions at *m/z* 179, 151, and 108 were formed due to the retrocyclisation pathway for bond 1, producing two ions at 179 [^1,2^A]^−^ and 151 [^1,3^A]^−^ and further loss of the (CO_2_) unit (44 Da). The above data is consistent with that of refs. ^36–38^. The LC-MS/MS fragmentation pattern of compound 38 is illustrated in Supplementary Fig. 40.

The stilbenes were exemplified in four peaks (**11**, **18**, **29**, and **39**). Compound (**39**) showed a [M − H + K]^−^ precursor ion at *m/z* 523.5026. The collision-induced dissociation (CID) spectrum of *m/z* 523.5026 generated an intense fragment ion peak at 455, arising from the loss of (CH_3_O). Accordingly, compound (**39**) was tentatively assigned as viniferether A. The fragmentation pattern was presented in Supplementary Fig. 41. Polydatin was the candidate for peak (**11**) detected in positive ESI mode at *m/z* 390.4025. Its MS/MS analysis exhibited a main peak at *m/z* 228, matching with the loss of hexose moiety (162 Da). The mass data was in line with a previous study^39^.

Anthocyanins, natural food pigments, are widely distributed in grapes^40^. Three compounds (**20, 25**, and **26**) were tentatively annotated as anthocyanins. Cyanidin-3-*O*-glucoside was proposed as compound (**20**) with a parent ion peak at *m/z* 449.4006. Its MS^2^ generated product ions at *m/z* 287 and 269, resulting from the loss of sugar unit (162 Da) followed by a water molecule (18 Da). The dehydrated aglycone was further fragmented, losing [C_2_H_2_O] ^+^ to form a key fragment ion at *m/z* 227, accompanied by H_2_O loss, furnishing an ion at *m/z* 209, which finally loses [C_4_H_3_]^+^ to give a product ion at *m/z* 155.

Compound (**25**) was tentatively characterized as peonidin-3-*O*-glucoside with a precursor ion peak at *m/z* 463.9001. The CID spectrum of *m/z* 463.9001 generated a major fragment ion of 301, indicating loss of the glucoside moiety (162 Da). The mass profile described above came in line with previous literature^41^.

Fatty acids, phosphatidylcholines, phosphatidylinositols, and phosphatidylethanolamines are the predominant lipids extracted from different grape samples.

Four fatty acids (**28**, **34**, **35**, and **48**) were tentatively detected in the current analysis. The saturated fatty acid myristic acid (**28**) with a molecular ion [M − H + Na]^−^ appeared at *m/z* 249.3723 along with MS^2^ putative fragmentation ions of *m/z* 155 and 113 generated upon loss of propionate ion (C_3_H_5_O_2_^•^) (73 Da) followed by propylene ion (C_3_H_6_^•^) (42 Da) from the hydrocarbon chain.

Four phosphatidylcholines (**30**, **31**, **32**, and **33**) were characterized in positive ionization mode [M + H]^+^. Compound **30** was recorded at *m/z* 494.6026 [M + H]^+^ and tentatively assigned as a PC (16:1/0:0). It has different fragment signals at *m/z* 311, 184, 125, and 104. The main signals at *m/z* 184, 125, and 104 were common among all phosphatidylcholines (**30**, **31**, **32**, and **33**) and corresponded to the fragmentation of phosphocholine C_5_H_14_NO_4_P^+^ ion (molar mass of ionic form, 184 Da), phosphate diester C_2_H_6_O_4_P^+^ ion (molar mass, 125 Da), and ethoxy trimethylamine (C_5_H_14_NO^+^) ion (104 Da). That profile was consistent with the fragmentation pathway mentioned in a previous study^42^.

Compound (**37**) was observed at *m/z* 571.6028 [M − H]^−^ and tentatively assigned as 1-hexadecanoyl-sn-glycero-3-phospho-(1’-myo-inositol) or PI (16:0/0:0). A series of fragment ions were yielded at *m/z* 391, 315, 255, 241, and 153. The fragment ion at *m/z* 391 was observed after loss of C_6_H_11_O_6_^•^ ion (179 Da). The fragment ion *m/z* 315 was generated from loss of myo-inositol phosphate moiety (C_6_H_12_O_9_P^•^), 259 Da. The product ion at *m/z* 255 reflected the presence of hexadecanoate ion (C_16_H_31_O_2_^•^, 255 Da). The cleavage of the C-O bond in the carboxyl group liberates C_16_H_31_O_2_^•^ (333 Da), resulting in a product ion with *m/z* 241. An additional ion at *m/z* 153 referring to [H_2_C = C(OH)CH2-PO_4_H]^−^ (154 Da) was recorded.

Under the current chromatographic conditions, phosphatidylethanolamines are the most abundant class among the detected lipids.

This class was represented in eight peaks (**41**, **42**, **43**, **44**, **45**, **46**, **47**, and **50**) eluted late at the end of the chromatogram. Compound **41** was tentatively annotated as PE (18:2/17:0) with precursor ion [M + 2H]^+^ of *m/z* 731.0054. The loss of C_17_H_33_O^•^ (253 Da) and C_2_H_7_NO_4_P (140 Da) gave a product ion peak at *m/z* 337. This data was in line with the fragmentation pathway reported in a previous investigation^43^.

In the current analysis, other phytochemical classes that were detected in relatively lower concentrations compared with the above-stated classes included sugars, coumarins, auxins, sesquiterpenoids, procyanidins, sterols, and terpenoids.

The LC-MS/MS spectra for the tentatively identified metabolites were included in the Supplementary Materials file (Figs. S3–S52).

### GNPS analysis

**Fig. 1:**
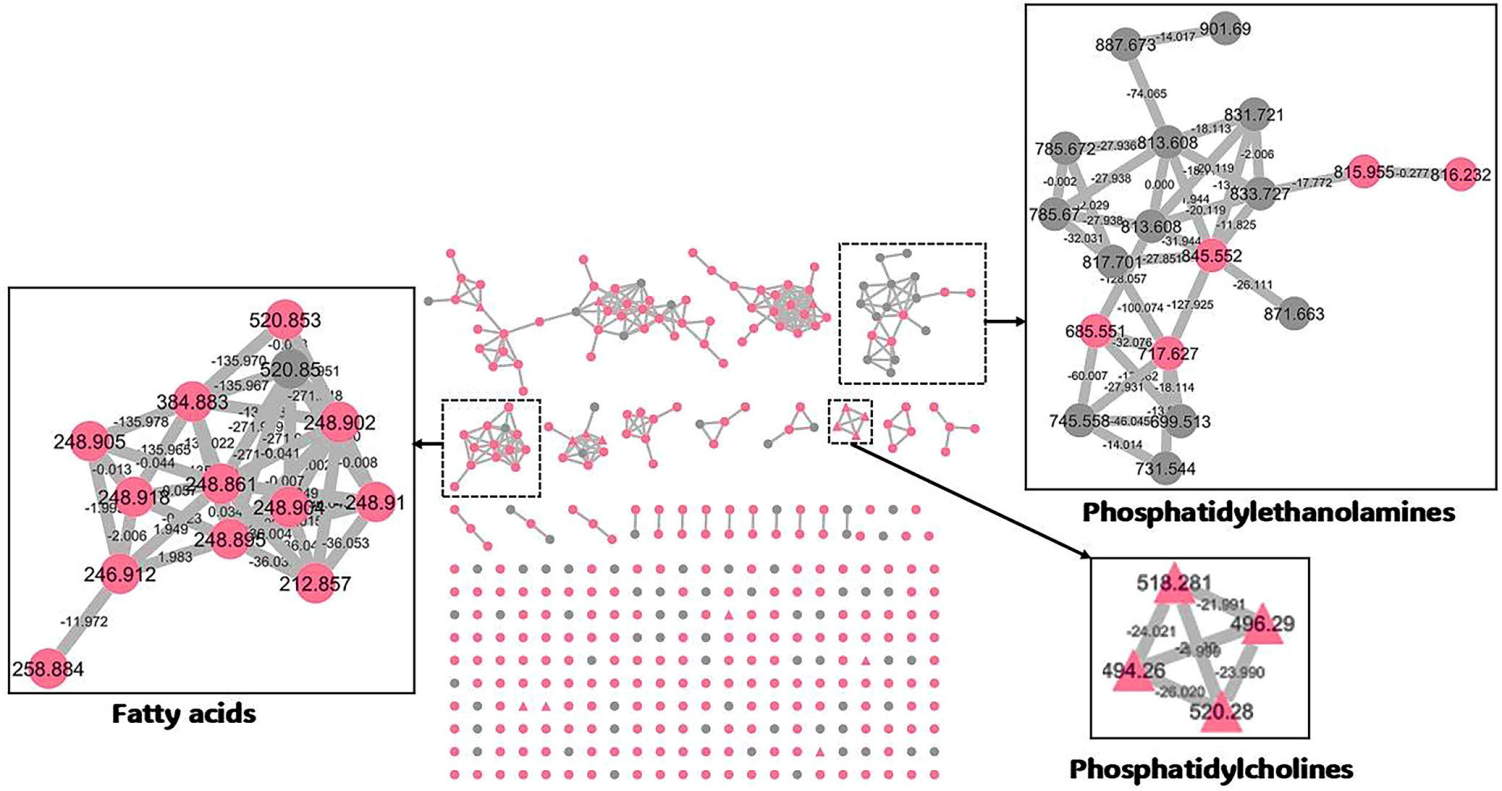
Molecular network for 401 metabolites of different grape samples. The Gray nodes refer to uncommon metabolites that were specific to only one sample, while the fuchsia nodes refer to the metabolites that were common among different samples.

The mass profiles of fresh and processed grape extracts were analyzed using the molecular networking database GNPS to display 401 metabolites, and their masses were represented as nodes, as shown in Fig. 1.

All nodes depicted the metabolites with distinctive peaks in the raw mass spectra, where the tentatively identified metabolites were presented in Table 1. Some metabolites are clustered together with correlated molecular weights and similar phytochemical classes to form a single cluster. Herein, we have seven major clusters. Three of them included fatty acids, phosphatidylcholines, and phosphatidylethanolamines. The tentative identification of these metabolites was confirmed by their clustering. PE (18:2/17:0), PE (16:0/18:2), PE (18:2/15:1), PE (18:2/22:6), PE (14:0/18:3), PE (18:2/PGE1), PE (18:1/18:1) and PE (18:2(10,12) + = O(9)/22:1(13)) were clustered together as phosphatidylethanolamines. Four fatty acids were detected, including myristic acid, hydroxytetracosanoic, myristic acid, 2-(1-octadecenyloxy) ethyl ester, and 7,10-DiHODE. The clustered phosphatidylcholines were PC (16:1/0:0), PC (18:2/0:0), PC (18:3/0:0), and PC (0:0/16:0). The four phosphatidylcholines were further confirmed using GNPS as their fragment signals matched with similar fragment signals in the GNPS library. The mirror plot images for this matching were shown in Figs. S32–35.

**Fig. 2:**
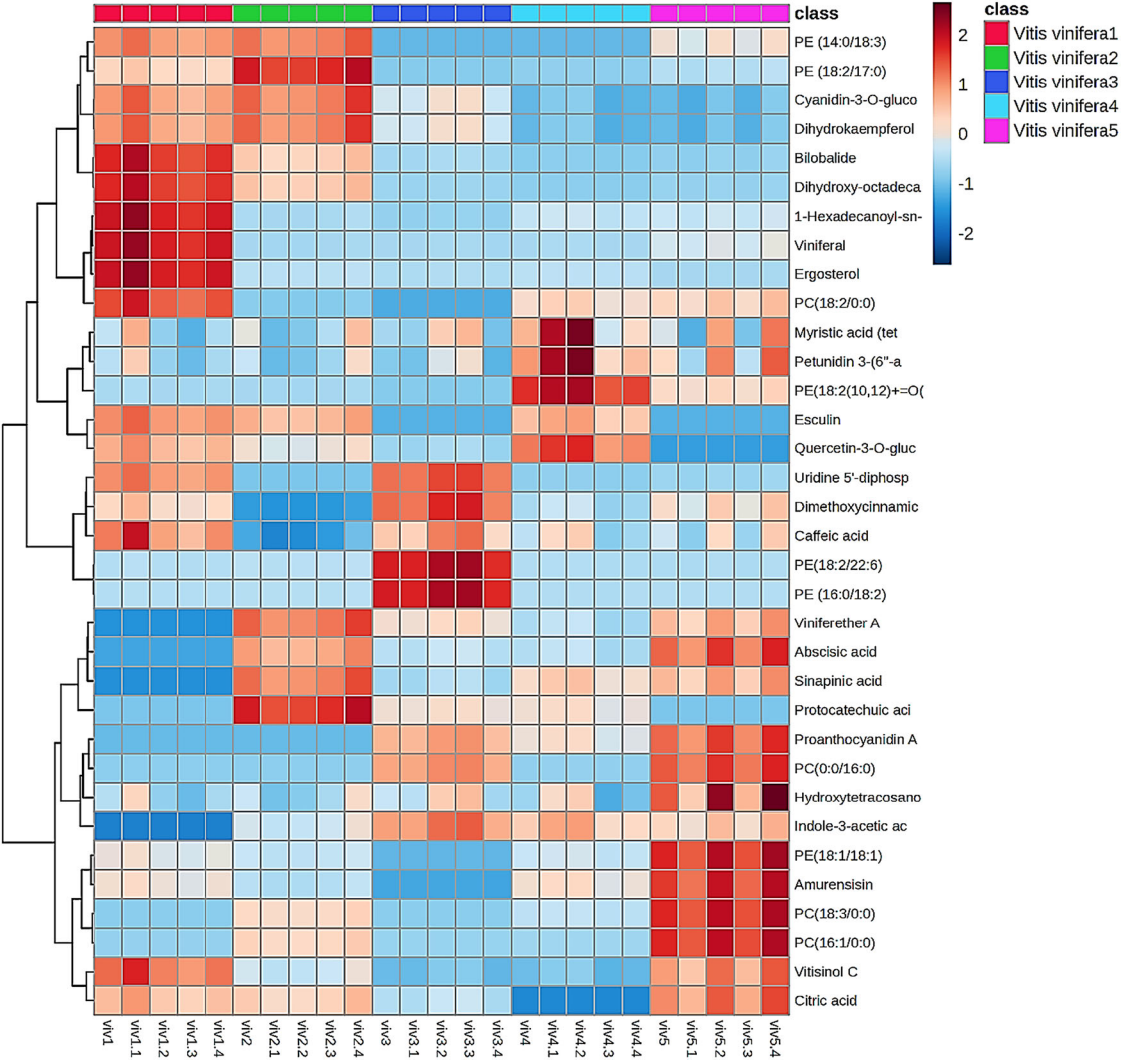
Heat maps of all tentatively annotated metabolites in different grape samples, grading from red to blue according to metabolites’ concentration from high to low. Vitis vinifera1: fresh grapes, Vitis vinifera2: grapes after 5 days of drying, Vitis vinifera3: grapes after 10 days of drying, Vitis vinifera4: grapes after 15 days of drying, Vitis vinifera5: raisins (after 20 days). The data are presented as the mean of five determinations (*n* = 5). Cyanidin-3-*O*-gluco cyanidin-3-*O*-glucoside, Dihydrokaempferol dihydrokaempferol glucoside, Dihydroxy-octadeca 7,10-dihydroxy-octadecadienoic acid (7,10-DiHODE), 1-Hexadecanoyl-sn 1-Hexadecanoyl-sn-glycero-3-phospho-(1’-myo-inositol), PI (16:0/0:0) myristic acid (Tet: Myristic acid (Tetradecanoic acid), Petunidin 3-(6”-a Petunidin 3-(6”-acetylglucoside), PE (18:2(10,12) + = O(PE (18:2(10,12) + = O(9)/22:1(13)), Quercetin-3-*O*-gluc quercetin-3-*O*-glucuronide, uridine 5’-diphosph uridine 5’-diphosphogalactose, Dimethoxycinnamic dimethoxycinnamic acid, Protocatechuic aci protocatechuic acid glucoside, Hydroxytetracosano hydroxytetracosanoic acid, Indole-3-acetic ac indole-3-acetic acid.

As depicted in Fig. 2, the heatmap uncovered the metabolic dynamics among the comparable samples, where a color-coded scale from red to blue stands for the peak area of each compound from high to low. In a more targeted approach, phenolic compounds, including flavonoids, stilbenes, and anthocyanins, as well as lipid species, primarily phosphatidylcholines, fatty acids, and phosphatidylethanolamines, were the predominant chemical classes existing in the five investigated samples. Concerning the fresh grape samples, it was observed that stilbenes and glycosylated anthocyanins majorly dominated the detected metabolites, while they experienced a slight decline over the drying process. On other domains, some lipid species, primarily fatty acids and phosphatidylcholines were comparably higher in raisin samples than others.

### Multivariate data analysis of the fresh and different processed grape samples

Owing to the multifaceted nature of metabolomics data, the implementation of multivariate statistical analyses seems imperative. In the current investigation, the gained mass data were analyzed using different multivariate statistical models.

PCA is an unsupervised approach that converts multivariate data into a few principal components (PCs), providing a general overview of variance, dispersion, and clustering trends in the dataset across the PC space^44^. As depicted in Supplementary Fig. 53, the PCA score plot revealed the five samples of fresh and different dried grapes distributed into two main clusters along PC1. The fresh (untreated) grapes (VIV1) and the grape samples gathered post 5 days of sun-drying (VIV2) were clustered altogether along the PC1 positive side. Meanwhile, the 10 and 15 days of sun-dried samples (VIV3 and VIV4), as well as the raisin samples (VIV5) were segregated along the negative direction of PC1, affirming their chemical discrepancies. Further, PC2 discriminated between (VIV3 & VIV4) samples and (VIV5) ones where the former samples (VIV3 &VIV4) were allocated in the upper left quadrant while the later ones (VIV5) were clustered in the lower left segment suggesting the more or less possible similarity in their chemical makeup (Supplementary Fig. 53).

**Fig. 3:**
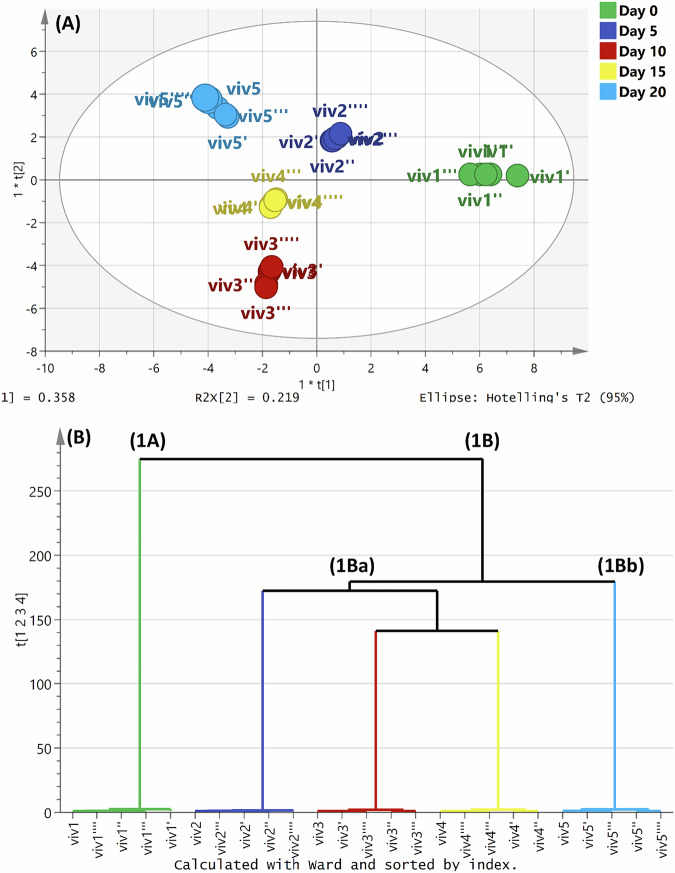
Discriminatory models of different grape samples. OPLS-DA score scatter (**A**) HCA dendrogram (**B**), according to the Ward method.

To allow superior discrimination among the explored samples, a supervised OPLS-DA model was used. Of note, the performance of the created model was monitored through the computed parameters “R^2^ (0.991)” and “Q^2^ (0.986),” assuring the good predictive capacity of the model. As presented in Fig. 3A, OPLS-DA, prescribed by five components, effectively discriminated the five grape samples into two to three clusters. OPLS-DA exhibited significant separation among the samples where the fresh grapes (VIV1) and the grapes in the first stage of drying (VIV2) were located in opposite direction far away from the grapes (VIV3 & VIV4) in the second and third stages of drying, as well as raisins (VIV5) confirming their quite compositional divergences. Further, the raisin samples (VIV5) were distantly clustered in the upper left quadrant and well-distinguished from the other samples. On the other domain, both VIV3 and VIV4 samples were noticed in the lower left quadrant. In agreement with the above OPLS-DA results, the dendrogram (Fig. 3B) acquired from Hierarchical clustering analysis (HCA) separated the five comparable samples into two major clusters referred to as groups **1A** and **1B**, respectively. Group “**1A**” corresponds to the fresh grape samples (VIV1) while the remaining four groups (VIV2, VIV3, VIV4, and VIV5) were clustered in the other group “**1B**”. Samples VIV2, VIV3, VIV4, and VIV5 were separated into two sub-clusters, **1Ba** and **1Bb**, revealing their compositional variances. The VIV5 samples were partially deviated from the others, suggesting their significant metabolic difference.

**Fig. 4:**
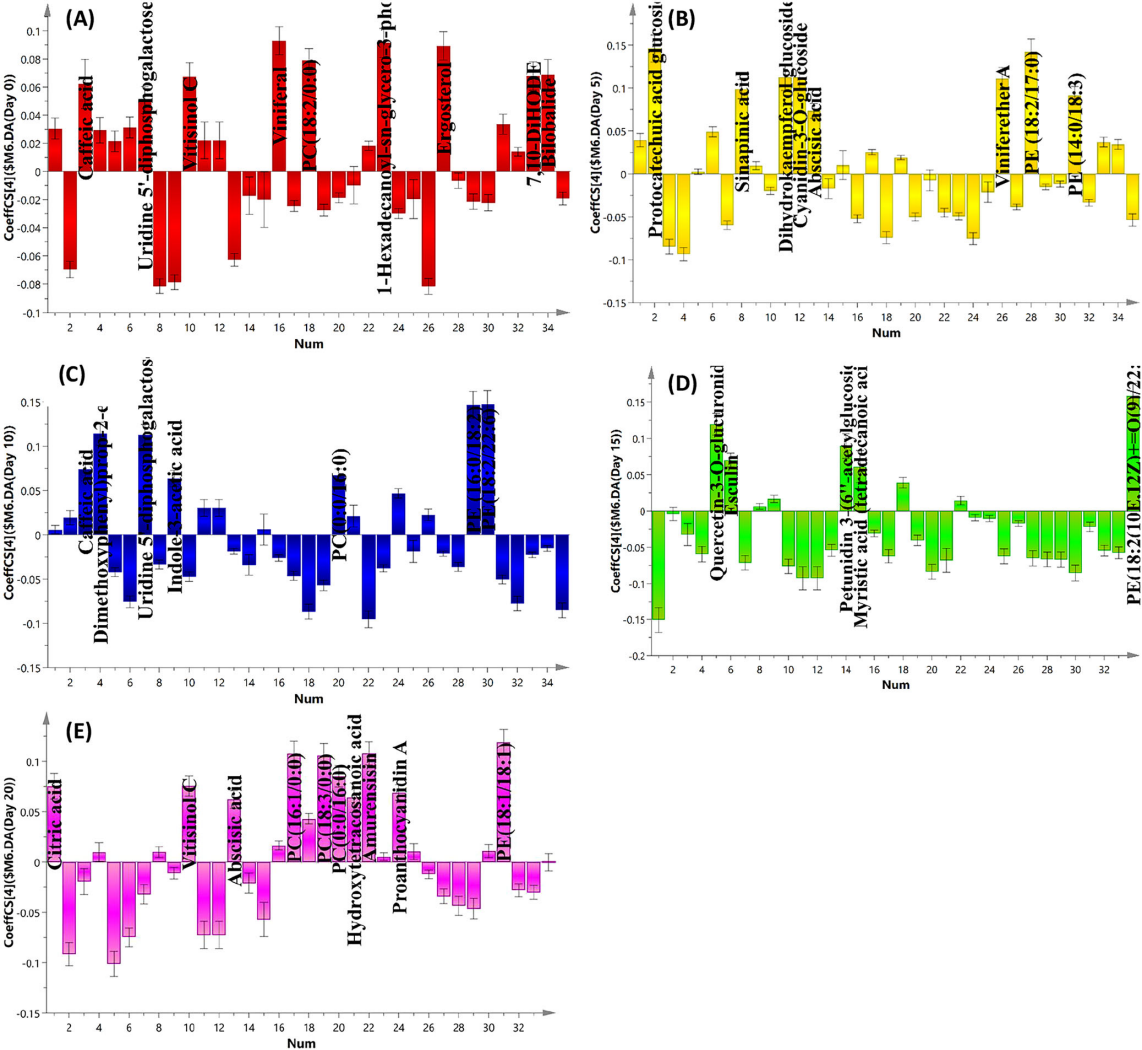
Coefficient plots of the constructed OPLS-DA model of grape samples. Fresh (untreated) grapes (**A**), sun-dried grapes after 5 days (**B**), sun-dried grapes after 10 days (**C**), sun-dried grapes after 15 days (**D**), and sun-dried grapes after 20 days raisins (**E**).

To rapidly catch the primary distinctive metabolites distinguishing between the five samples, the OPLS-DA derived coefficient plots were implemented (Fig. 4). In Fig. 4A the influential metabolites enriched in fresh grape samples (VIV1) were caffeic acid, uridine 5’-diphosphogalactose, vitisinol C, viniferal, PC (18:2/0:0), PI (16:0/0:0), ergosterol, 7,10-DiHODE, and bilobalide. On the other domain, the noticeable metabolites majorly detected in grape samples (VIV2) included protocatechuic acid glucoside, sinapinic acid, dihydrokaempferol glucoside, cyanidin-3-*O*-glucoside, abscisic acid, viniferether A, PE (18:2/17:0), and PE (14:0/18:3) (Fig. 4B). Figure 4C showed the enrichment of compounds namely caffeic acid, dimethoxycinnamic acid, uridine 5’-diphosphogalactose, indole-3-acetic acid, PC (0:0/16:0), PE (16:0/18:2), and PE (18:2/22:6) in grape samples (VIV2). Successively, inspection of the coefficient plot of grape samples (VIV4) (Fig. 4D) suggested MS peaks for quercetin-3-*O*-glucuronide, esculin, petunidin 3-(6”-acetylglucoside), myristic acid (tetradecanoic acid), and PE (18:2(10,12) + = O(9)/22:1(13) as the discriminatory markers of these samples. The coefficient plot presented in Fig. 4E illuminated that citric acid, vitisinol C, abscisic acid, PC (16:1/0:0), PC (18:3/0:0), PC (0:0/16:0), hydroxytetracosanoic acid, amurensisin, proanthocyanidin A, and PE (18:1/18:1) were the main metabolites accumulated in raisins (VIV5). Compared to previous studies, the phenolic profile of raisin was similar to that of fresh grape, and the difference between them was only quantitative^45^. Furthermore, the anthocyanins were reported to appear only in grape skin, and their concentration in fresh grapes was greater than in raisins due to degradation during sun drying^45^. Another study was conducted on raisins to evaluate the influence of drying on the fatty acid profile. During the drying process, approximately sixteen fatty acids were detected and quantified. Some of them (C16:1, C17:0, C24:0, and C23:0) were not characterized in fresh grapes^46^.

### In vitro antidiabetic activity

Diabetes is a chronic disorder associated with multiple complications, including cardiovascular diseases, cancer, blindness, obesity, kidney and nerve damage, and limb amputation^47,48^ due to immunological disruptions caused by abnormal metabolism. Inhibiting *α*-glucosidase, a crucial intestinal enzyme, lowers glucose levels and delays carbohydrate digestion^49^. *α*-amylase is a glycoside hydrolase enzyme excreted by salivary glands^50^. *α*-amylase could be considered a marker for diabetes diagnosis^50^. Their levels are higher in diabetes than in healthy people due to excessive glucose production^50^. The inhibition of intestinal α-amylase and α-glucosidase activities are two in vitro antidiabetic assays commonly used to ascertain the antidiabetic potential of natural products^49^.

Red varieties of grape were proven to exert remarkable antidiabetic activities compared with other varieties^51^. Some previous reports have demonstrated the promising *α*-amylase and *α*-glucosidase inhibitory activities of red grapes as being enriched with potential bioactive compounds, primarily procyanidins, flavonoids, anthocyanins, phenolics, catechins, and tannins^51^.

**Fig. 5:**
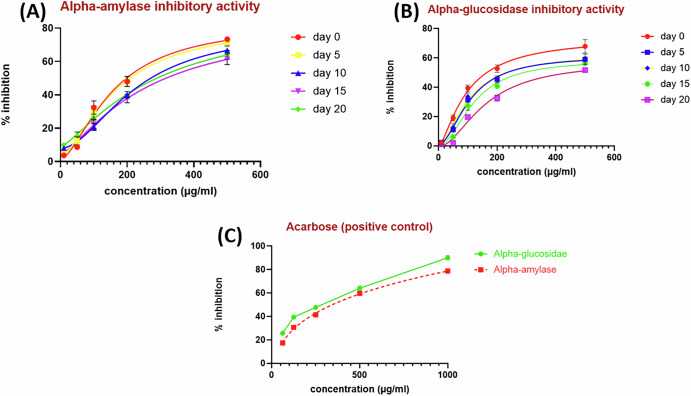
Dose response curves. α-Amylase inhibition capacity of the five tested grape samples (**A**); α-glucosidase inhibitory activity of the five tested grape samples (**B**); and α-amylase and α-glucosidase inhibitory activity of the positive control (acarbose) (**C**).

In our investigation, all the examined samples exhibited prominent inhibitory activity against both *α*-amylase and *α*-glucosidase enzymes in a dose-dependent manner, as depicted in Fig. 5. The fresh grape extracts exerted a noteworthy inhibition capacity against both *α*-amylase and *α*-glucosidase with IC_50_ values of 157.5 ± 0.56 and 91.89 ± 0.47 μg/mL, respectively, compared to the positive control acarbose (355.0 ± 0.65 μg/mL) in the case of α-amylase and (253.0 ± 0.83 μg/mL) in the case of *α*-glucosidase. Equally important, the sun-dried grape samples in the early phase (after Days 5 and 10) revealed roughly comparable inhibitory potential against *α*-glucosidase enzyme with IC_50_ (104.4 ± 1.05 and 107.1 ± 0.62 μg/mL), respectively. Relatedly, both samples displayed inhibitory actions against *α*-amylase with IC_50_ values of 168.1 ± 0.73 and 207.9 ± 0.52 μg/mL, respectively. Successively, the samples of dried grapes (after Day 15), as well as raisins, exerted moderate inhibitory activities against both *α*-amylase and *α*-glucosidase enzymes with respective IC_50_ values of 259.2 ± 1.05 μg/mL and 271.9 ± 0.73 μg/mL against *α*-amylase, as well as 121.2 ± 0.49 μg/mL and 160.4 ± 0.92 μg/mL against *α*-glucosidase.

Taken together, it was observed that the drying process of grapes negatively affects both *α*-glucosidase and *α*-amylase inhibitory activities, and the fresh grapes surpassed the antidiabetic activity of the positive control (acarbose).

These results were consistent with the previous investigations, which proved the superior antidiabetic activity of the fresh grapes. In a previous study, the inhibitory capacity of *α*-amylase and *α*-glucosidase red grape skin extract were estimated and represented by IC_50_ as 3.06 ± 0.30 and 1.06 ± 0.16 μg/mL, respectively in a comparable manner to that of acarbose (positive control) which was 3.91 ± 0.44 μg/mL in case of *α*-amylase and 1.75 ± 0.14 μg/mL in case of *α*-glucosidase^52^. Another in vivo study aimed to investigate the anti-diabetic and anti-inflammatory efficacy of grape-derived stilbene concentrate on 40 male Wistar rats with metabolic syndrome induced by the fructose model. The usage of the grape-derived stilbene concentrate had positive changes in carbohydrate and lipid levels, and inflammatory markers. It also mitigated the associated inflammation and cardiac damage^53^.

**Fig. 6:**
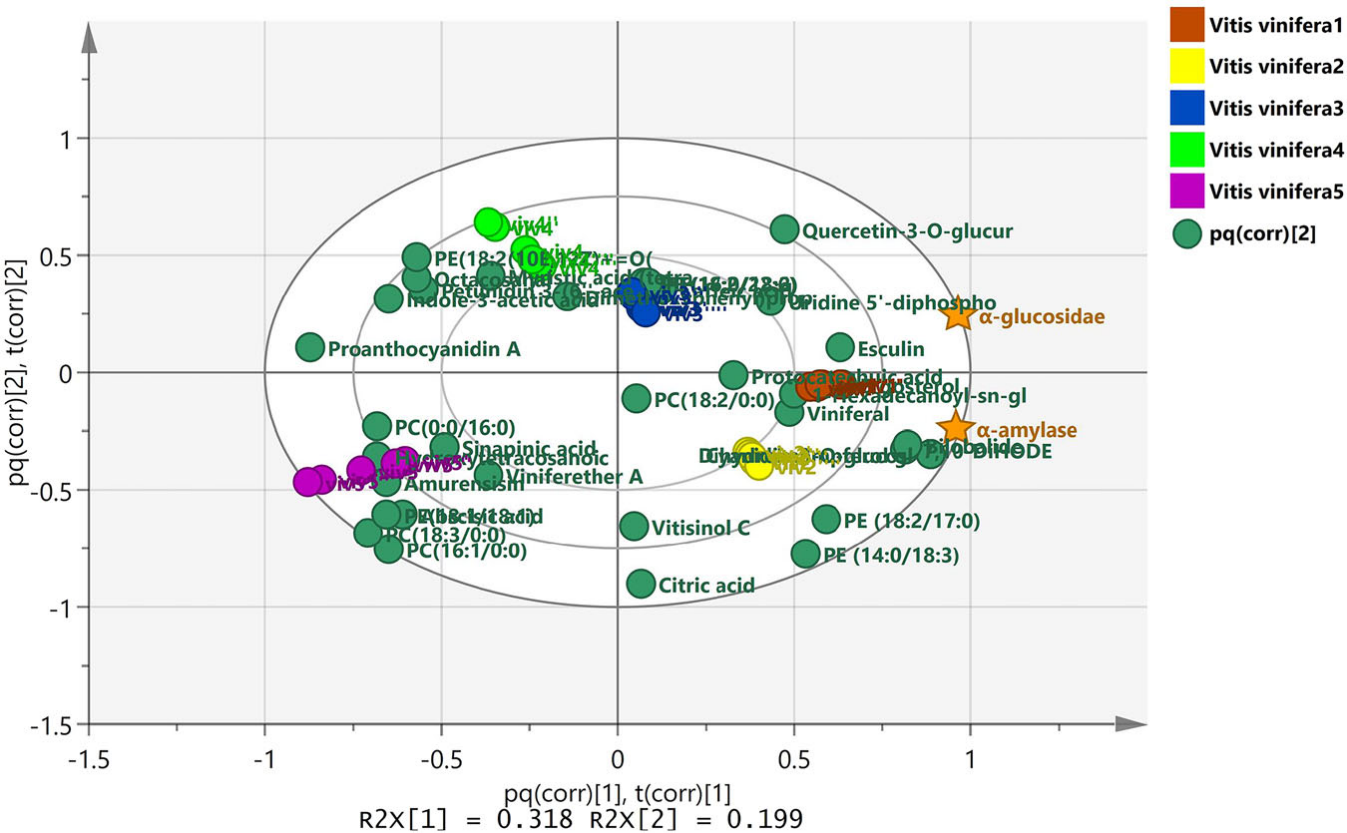
OPLS biplot showing inhibitory activity of the examined samples against α-amylase and α-glucosidase enzymes. pq(corr) p; loading weight for X and q; loading weight for Y, scaled as the correlation coefficient between X, Y in the OPLS model.

OPLS analysis was established by merging MS/MS data as *X* variables with *α*-glucosidase and *α*-amylase inhibition capacity as *Y* variables to readily detect antidiabetic metabolites in the investigated samples. The OPLS model was validated by calculating Q^2^ (predicted variance) and R_2_Y (explained variance), which were 0.981 and 0.983, respectively, revealing the model's reliability. OPLS biplot model (Fig. 6) shows the spatial correlation between discriminated metabolites and the corresponding antidiabetic impact of the five explored samples. According to Fig. 6, the fresh grape sample (VIV1) was projected close to *α*-glucosidase and *α*-amylase inhibitory effects, indicating that they were strongly correlated. This was in line with IC_50_ results of fresh grapes (VIV1) against both *α*-glucosidase and *α*-amylase enzymes, which were much better than acarbose (positive control). Furthermore, raisin samples (VIV5) with the least activity were the most distant from *α*-glucosidase and *α*-amylase inhibitory effects in the biplot chart. Given these outcomes, we hypothesized that anti-diabetic activity decreased during grape drying, to be the least with raisins.

**Fig. 7:**
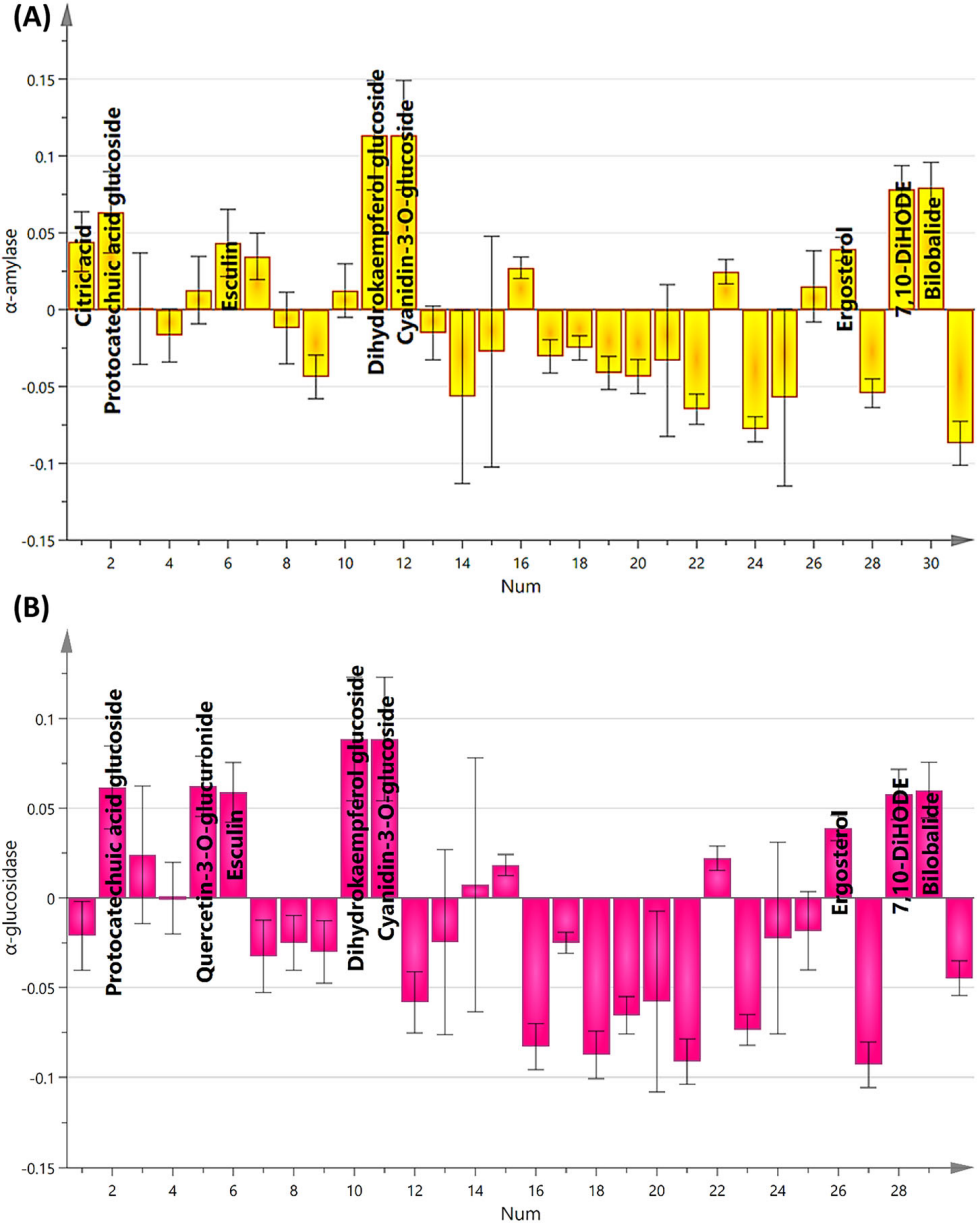
OPLS-derived coefficient plots representing putative biomarkers responsible for antidiabetic activity. **A** α-Amylase and **B** α-glucosidase.

As shown in Fig. 7, OPLS-derived coefficient plots were implemented to better pick out the metabolites that were possibly associated with the noticed antidiabetic activity.

More specifically, the main contributors beyond the antidiabetic activity might be phenolic compounds as quercetin-3-*O*-glucuronide, dihydrokaempferol glucoside, protocatechuic acid glucoside, and cyanidin-3-*O*-glucoside, that prevalently found in fresh grapes (highest active samples). Additionally, other compounds belonging to different classes, including ergosterol, 10-DiHODE, citric acid, esculin, and bilobalide, are recognized för their positive impact. While in the case of *α*-glucosidase inhibitory activity (Fig. 7).

These outcomes were consistent with previous studies, which demonstrated the superior *α*-glucosidase and *α*-amylase inhibitory effects of flavonoids and phenolics^54^. In an in vitro anti-diabetic assay, kaempferol-3-O-rhamnoside displayed remarkable inhibition of glycation with IC_50_ (84.27 ± 11.38 μg/mL)^55^. Cyanidin-3-*O*-glucoside is a promising anthocyanin with an eminent inhibitory effect against *α*-glucosidase enzyme with IC_50_ = 479.8 μM^56^. In a streptozotocin-induced diabetic mouse model, the oral administration of soluble starch with cyanidin-3-*O*-glucoside significantly decreased the levels of postprandial blood glucose^57^. Similarly, quercetin-3-*O*-glucuronide was proven to be an effective hypoglycemic agent for diabetes management as it effectively suppressed the glycation products such as 5-hydroxymethylfurfural, fructosamine, and advanced glycation end products^58^. Equally important, protocatechuic acid exerted an acclaimed postprandial hypoglycemic activity in diabetic mice^58^. A recent in vivo study has suggested that ergosterol could be applied in the treatment of type 2 diabetes as a potential hypoglycemic agent^59^. In a streptozotocin-injected rat model, ergosterol treatment attenuated diabetic nephropathy *via* significantly suppressing different biochemical markers, including uric acid, plasma glucose, triglyceride, total cholesterol, creatinine, and cytokines (IL-6, TNF-α, and MCP-1) levels^60^.

Esculin displayed in vitro pancreatic lipase inhibition in a dose-dependent manner with an IC_50_ value of 11.6 ± 0.92 μg/mL compared to that of orlistat (positive control), which showed an IC_50_ of 0.114 ± 0.01 μg/mL^61^. The administration of bilobalide decreased blood glucose levels and exhibited anti-retinopathy activity in the streptozotocin-induced diabetic rats^62^.

Collectively, the current work was concerned with tracking the metabolic profile of *Vitis vinifera* (Crimson Seedless) grapes over the course of the drying process in parallel with evaluating their antidiabetic activity through UPLC-MS/MS coupled with multivariate statistical analyses. Given this analysis, approximately 50 metabolites categorized under several chemical classes comprising organic acids, phenolic acids, flavonoids, stilbenes, sugars, anthocyanins, terpenoids, coumarins, auxins, sesquiterpenoids, procyanidins, sterols, terpenoids, as well as some lipid species, were tentatively identified from fresh and dehydrated grape extracts. Different phenolic compounds, as well as lipid species, were the prevalent classes existing in the investigated samples. Experimentally speaking, all samples (VIV1, VIV2, VIV3, VIV4 and VIV5) exhibited prominent inhibitory activity against both *α*-amylase and *α*-glucosidase enzymes in a dose-dependent manner with the most leading outcomes were noted in the fresh grape extracts with IC_50_ values of 157.5 ± 0.56 and 91.89 ± 0.47 μg/mL, respectively which was greater than the positive control (acarbose). OPLS-derived coefficient plots illuminated that citric acid, protocatechuic acid glucoside, esculin, dihydrokaempferol glucoside, cyanidin-3-*O*-glucoside, ergosterol, 7,10-DiHODE, citric acid, and bilobalide might be the main contributors that collectively contribute to the underlined evident anti-diabetic activity.

Conducting deeper in vivo anti-diabetic investigations, as well as clinical trials on fresh grapes and raisins, is highly recommended to explore a promising hypoglycemic functional food. Additionally, quantifying the daily amount suggested for both healthy and diabetic people from grapes and raisins is urgently needed.

## Methods

### Plant material acquisition

The fresh samples of Crimson Seedless grape (*Vitis vinifera*) were purchased from the wholesale market for vegetables and fruits located in Hosny Mubarak Street, Shibin El-Kom, Menoufia Governorate 6132234, Egypt.

### Grape drying process

**Fig. 8:**
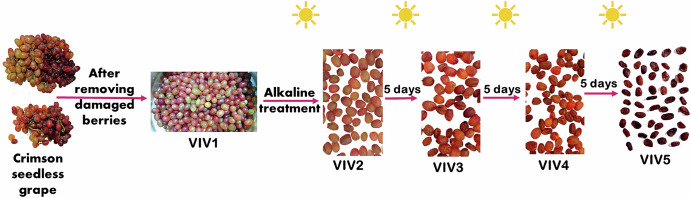
Visual representation of the three-week-long Crimson Seedless grape drying process till raisin production.

Fresh Crimson Seedless grapes (9000 g) were bought in December 2023. After removing the damaged or diseased berries and discarding the stems, only 6900 g of fresh, suitable grapes remained. About 400 g were kept fresh and directly subjected to the extraction process. The remaining amount was sun-dried after pre-processing treatment to produce raisins. The alkaline pre-processing treatment was conducted as described previously^63^ with some modifications. Grape berries were immersed in 0.3% sodium hydroxide solution at 100 °C for 3 s and immediately rinsed with cold water before drying. The sun drying procedure was conducted according to ref. ^64^ with some modifications. After alkaline treatment, berries were distributed on a cloth and subjected to sunshine and outside conditions. The average humidity and temperature were 24%, 14 °C (low) and 23 °C (high), respectively. Berries were completely dried within three weeks, yielding dry raisins. As depicted in Fig. 8, four samples labeled with (VIV2, VIV3, VIV4, and VIV5) were collected at distinctive drying stages (every five days) as shown in Supplementary Table 1, and submitted to the extraction process. The remaining amount of raisins was stored in the refrigerator for any further analyses.

### Preparation of plant extracts

Four hundred grams of fresh grape berries (VIV1) were rinsed with tap water and then blended in 800 mL 70% ethanol (1:2 *w*/*v* for 10 min using (Panasonic MX-119N Super Mixer Grinder). After that, the mixture was sonicated for 30 min at 50 °C using a Cole-Parmer Ultrasonic Homogenizer (CPX400). The extraction process was conducted in triplicate, and the resulting extracts were filtered using metal refinery first, then filter paper (Whatman Quantitative Filter Paper, 125 mm, Grade Egypt). The combined filtrates were concentrated using a rotary evaporator (Heidolph VV2000) at 40 °C and 120 rpm. After that, the partially dried residue was placed in a Heraeus electric oven at 40 °C till reaching a constant weight (complete drying). Analogously, 400 g of the collected four samples (VIV2, VIV3, VIV4, and VIV5) were extracted using the same procedure described above to get dry residues.

### Chemical characterization of different grape extracts using UPLC–QTOF-MS/MS analysis

One mg of each extract was dissolved in 1 mL pure ACN (HPLC-grade acetonitrile from VWR chemicals), sonicated for 15 min, and then centrifuged for 3 min at 13,000 rpm. After that, the supernatant was diluted using Mill-Q® water with 0.1% formic acid to reach 30% ACN and centrifuged again. The 20 µL of each sample solution was packed into an insert of a glass vial and placed in the samples’ rack of the machine. The 2 µL of each sample was injected and analyzed using ultra-performance liquid chromatography coupled with quadrupole time of flight mass spectrometry PLC-QTOF- UPLC-QTOF-nanospray MS (Waters Nano Acquity, QTOF Micro (75 µm × 250 mm, 1.7 µm), BEH130 C18) set up at 30 °C. The chromatographic separation was conducted using a gradient eluent of 5–90% acetonitrile in % 0.05 formic acid over 10 min (8 min for the gradient run and 2 min for column conditioning). The flow rate was 0.1 mL/min. The biphasic mobile phase comprised of acidified ultrapure water (0.1% formic acid) (Phase A) and acidified acetonitrile (0.1% formic acid) (Phase B) was gradient eluted as follows: 0–1 min, 5–20% B; 2–4 min, 20–60% B; 4–6 min, 60- 60% B; 6–8 min, 60–100% B with a post-run of 2 min to equilibrate the system.

Mass spectrometry was conducted in both positive and negative electrospray ionization (ESI) modes over a mass range of 100–1200 *m*/*z*. The optimized parameters for nano-ESI interface were as follows: capillary voltage of 4300 V, cone voltage; 35 V, the ion source temperature was 150 °C, the nebulizer (nitrogen gas) pressure was 35 psi, drying and sheath gas (N_2_) temperatures were 440°C and 350°C, respectively. The drying and sheath gas flows were applied at 900 L/h and 50 L/h, respectively. In MS^E^ mode, three collision energies of 10 eV, 20 eV, and 40 eV were used to attain full tandem mass spectra.

Of note, 5 µL from each examined sample was pooled to generate a quality control (QC) sample for judging the stability and robustness of the analytical platform. Five replicates of each sample were utilized in the current investigation.

The mass spectroscopy raw file data acquired from the QTOF machine was analyzed using “the Global Natural Products Social Molecular Networking (GNPS)” dataset. GNPS helps to identify and discover metabolites by comparing the mass fragmentation pattern from raw mass spectrum to the GNPS library, with the help of MSConvert (MSConvertGUI (64-bit)) installed from the ProteoWizard site. Then, FileZilla was utilized for the secure transfer of the converted files from the local computer to the GNPS server (https://gnps.ucsd.edu/ accessed on 22 January 2025)^65,66^. After that, Cytoscape version 3.5.1 (https://cytoscape.org) permitted the visualization of the final molecular network following GNPS analysis. Molecular networking was accomplished using the GNPS data analysis workflow using the default parameters, the precursor mass tolerance 2.0 Da, fragment ion mass tolerance 0.5 Da, the minimum peak intensity was 0.0, a minimum of 6 matched peaks, minimum cluster size 2, a minimum of 6 matched fragment ions, and a cosine score of 0.7^67,68^.

The data was processed using MZmine-2.53 (https://github.com/mzmine/mzmine2/releases/tag/v2.53) analysis software. The processing parameters were established as follows: mass range 100–1200 Da, mass tolerance 1.0 Da, RT tolerance (min) 1.0, group intensity threshold 5.0E2, and min highest intensity 3.0E5. Subsequently, a data matrix containing the retention duration, mass-to-charge ratio (*m/z*), and normalized peak area was created. Lastly, metabolite annotations were established based on matching MS/MS fragmentation patterns from the raw MS files with the self-built database and relevant literature data. Collecting the literature database was carried out using the Dictionary of Natural Products website, Google Scholar, PubMed, MDPI, Web of Science, and Wiley publications. Of note, all of these resources were used to demonstrate high confidence in annotation that follows the MSI criteria.

### In vitro antidiabetic activity

*Saccharomyces cerevisiae α*-glucosidase (EC.3.2.1.20) inhibitory activity assay was conducted according to a previously reported procedure with some modifications^69^. The buffer solution of potassium phosphate at pH = 7.4 was utilized to prepare the *α*-glucosidase solution. Samples were properly prepared in 1 mL of 0.1 M phosphate buffer. Then, they were serially diluted with dH_2_O to get the needed working concentrations of 0.025, 0.05, 0.125, 0.25, 0.5, and 1 mg/mL (test), dH_2_O (blank), and the positive control was acarbose (10 mM). A series of different concentrations was utilized in the current assay based on relevant reports of different fruit extracts, as well as different preliminary trials. Subsequently, Bovine pancreatin enzyme solution (5 mg/0.5 mL in 0.1 M phosphate buffer) was mixed with the reaction mixture in equal amounts and then incubated for 20 min at 35 °C. After adding 50 μL of PNPG (5 mM), the reaction persisted for 15 min. Finally, 1 M Na_2_CO_3_ was added to end the reaction. The obtained color was measured spectrophotometrically at 400 nm. The *α*-glucosidase inhibitory activity of the examined grape samples was expressed using IC_50_ (μg/mL) and conducted in triplicate, with values ± standard deviation. The software Prism 8.0 (GraphPad Software) was utilized to get the median growth inhibitory concentration by nonlinear regression analysis of the logarithm of concentration as a function of the normalized response.

Porcine pancreatic *α-*Amylase (EC 3.2.1.1) inhibitory activity was conducted according to ref. ^69^. The buffer solution of potassium phosphate at pH = 6.9 was utilized to prepare the *α*-Amylase solution. 50 μL of *α*-amylase (5 mg/0.5 mL in 0.1 M phosphate buffer), 10 μL of each extract sample at various concentrations 0.025, 0.05, 0.125, 0.25, 0.5, and 1 mg/mL (test) or the control (sodium phosphate buffer at pH 7.4), were appropriately mixed and poured into a 96-well plate. A series of different concentrations was utilized in the current assay based on relevant reports of different fruit extracts, as well as different preliminary trials. The plates were incubated for 45 min after adding 50 μL of dextrin substrate solution (1% *w*/*v*). Afterward, the absorbance was monitored at 580 nm after adding 100 μL of glucose kit reagents (AAP, 1 mM/L, GOD > 20KU/L, and NaN3, 8 mmol/l) to the plates. Acarbose was used as a positive control (10 mM). The grape samples' *α*-amylase inhibition activity was expressed using IC_50_ (μg/mL) and conducted in triplicate, with values ± standard deviation.

### Statistical analysis

The data was interpreted statistically using GraphPad Prism v8 (GraphPad Software, San Diego, California, USA) and expressed as the mean ± standard deviation through one-way analysis of variance, where *P* < 0.05. After that, SIMCA-P version 14.0 software (Umetrics, Sweden) was utilized successfully to conduct a series of innovative multivariate analyses on the acquired MS/MS data. Initially, “principal component analysis (PCA)” was performed to analyze the clustering trend and provide a comprehensive overview of the complex metabolomic data. Consequently, supervised discriminant analysis, such as “orthogonal projection to latent structures-discriminant analysis (OPLS-DA),” was accomplished to fully screen the variances among various grape samples and clearly pick out their discriminatory metabolites or markers. Furthermore, “Hierarchical clustering analysis (HCA)” was utilized for grouping a set of metabolomic data into different clusters based on their chemical discrepancies. Heatmap analysis was also accomplished using MetaboAnalyst 6.0 (https://www.metaboanalyst.ca/MetaboAnalyst/ModuleView.xhtml/) to visually uncover the relative abundance of the metabolites among the comparable samples. Complementarily, OPLS and its derived coefficient plots were utilized to evidently discern the biologically relevant compounds that probably underlie the observable antidiabetic activity.

## Supplementary information

Supplementary materials

## Data Availability

The data generated from this work is available and can be provided upon request to the corresponding authors.
