# Supplementary material for: Metabolomic and chemometric profiling of crimson grapes during drying and its impact on antidiabetic activity
Source: NPJ Sci Food. 2025 Jul 23;9:148. doi: 10.1038/s41538-025-00509-5 (PMC12284023; doi:10.1038/s41538-025-00509-5)
Supplement: Supplementary file 1 — Supplementary materials [file 41538_2025_509_MOESM1_ESM.docx]

**Supplementary materials**

Supplementary Table 1. Grape samples' codes and their indications

| **Sample name** | **Indication** | **Code** |
| --- | --- | --- |
| *Vitis vinifera*1 | Fres grapes (Day 0) | VIV1 |
| *Vitis vinifera*2 | Grapes after 5 days of drying (Day 5) | VIV2 |
| *Vitis vinifera*3 | Grapes after 10 days of drying (Day 10) | VIV3 |
| *Vitis vinifera*4 | Grapes after 15 days of drying (Day 15) | VIV4 |
| *Vitis vinifera*5 | Raisins (Day 20) | VIV5 |


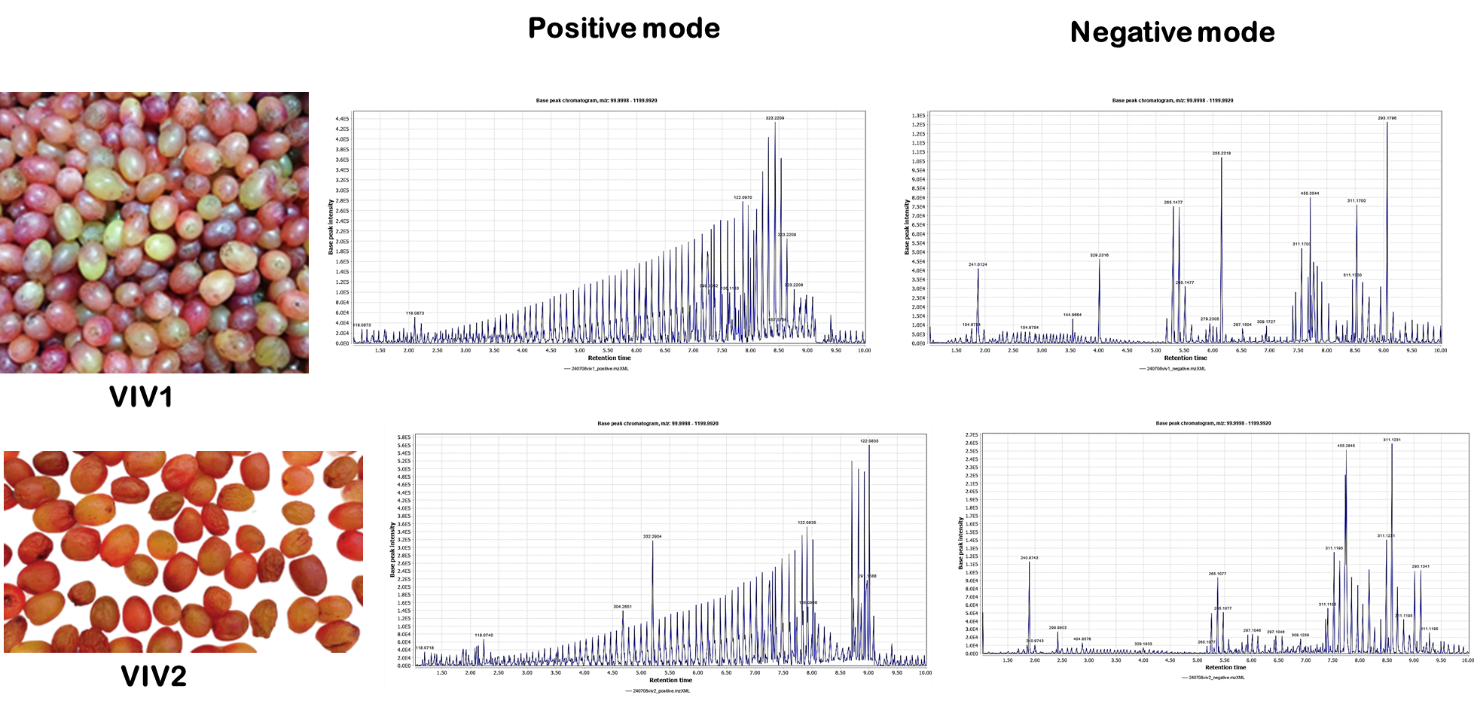

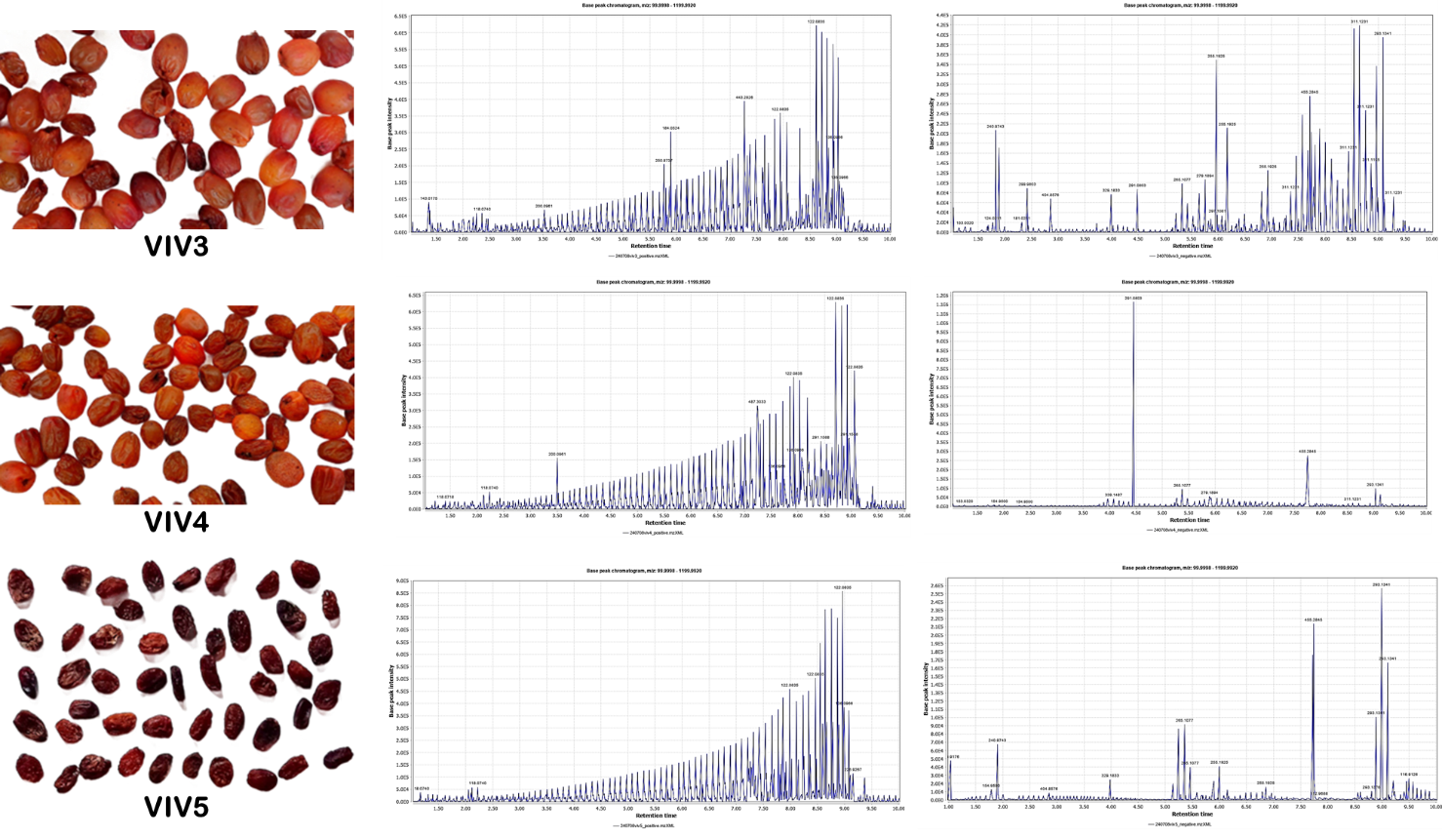


Supplementary Figure 1. Base peak chromatograms (BPCs) of the fresh (untreated) and the different sun-dried grapes collected at distinctive drying stages.

VIV1: Fresh grapes, VIV2: Grapes after 5 days of drying, VIV3: Grapes after 10 days of drying, VIV4: Grapes after 15 days of drying, VIV5: Raisins (after 20 days).

Supplementary Figure 2. Chemical structures of the identified phytochemical compounds from fresh and dried grape samples


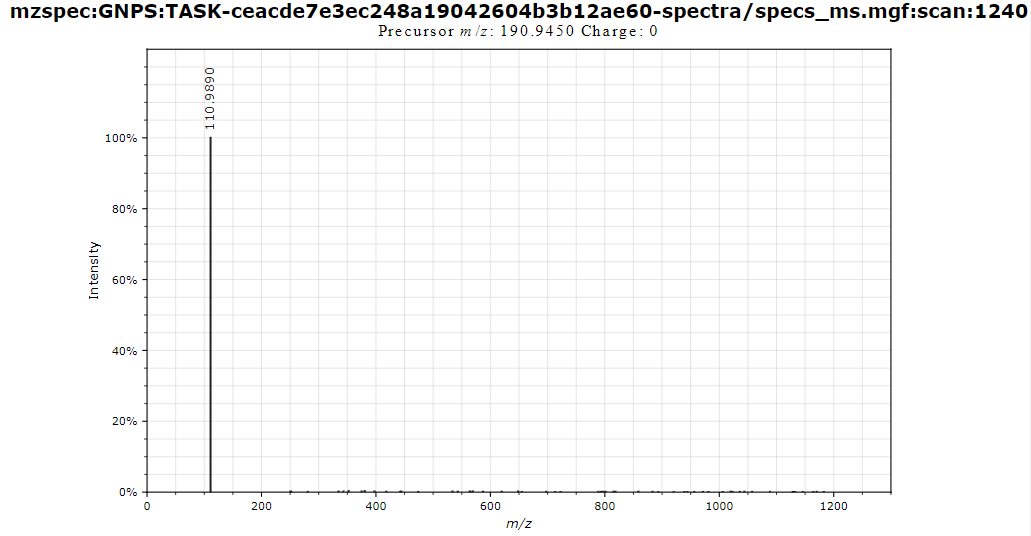


Supplementary Figure 3. LC-MS/MS of citric acid


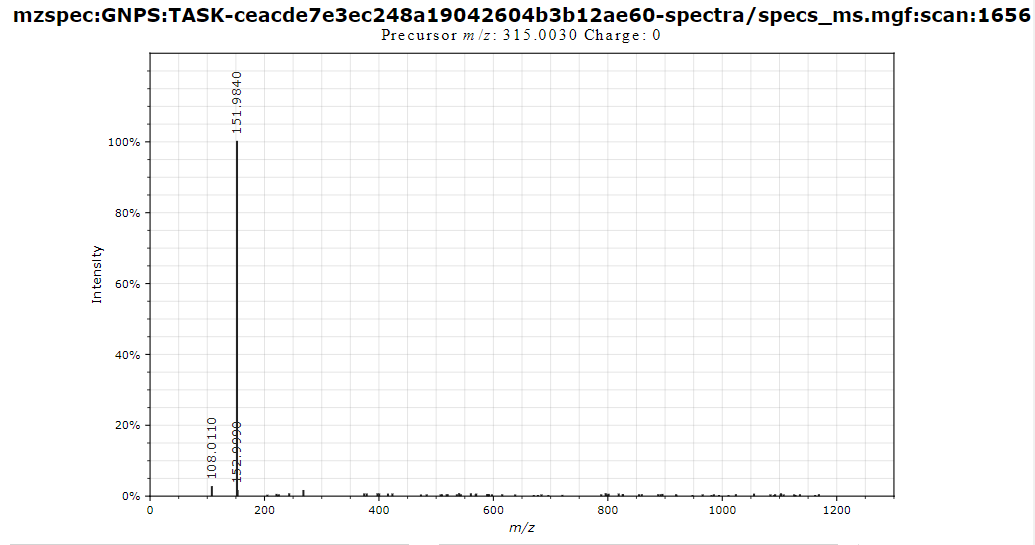


Supplementary Figure 4. LC-MS/MS of protocatechuic acid glucoside


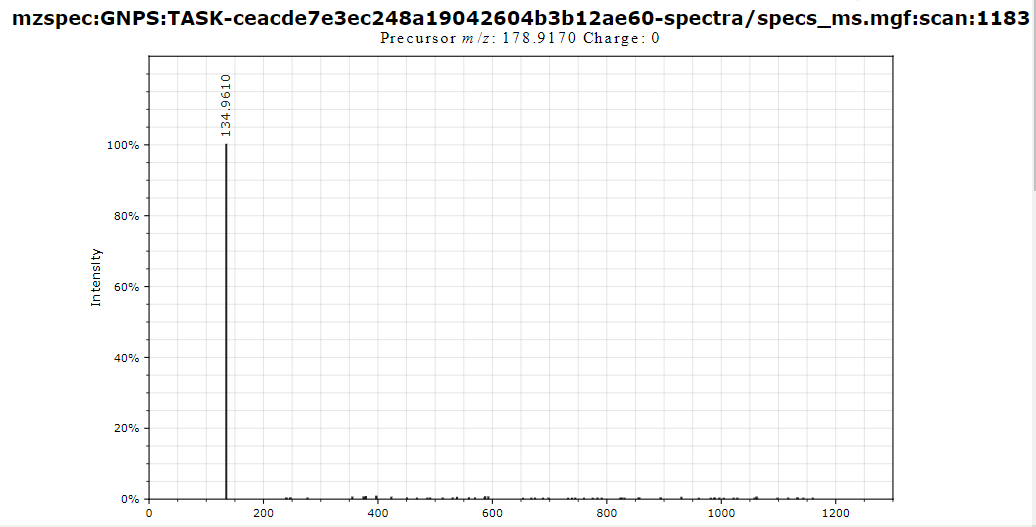


Supplementary Figure 5. LC-MS/MS of caffeic acid


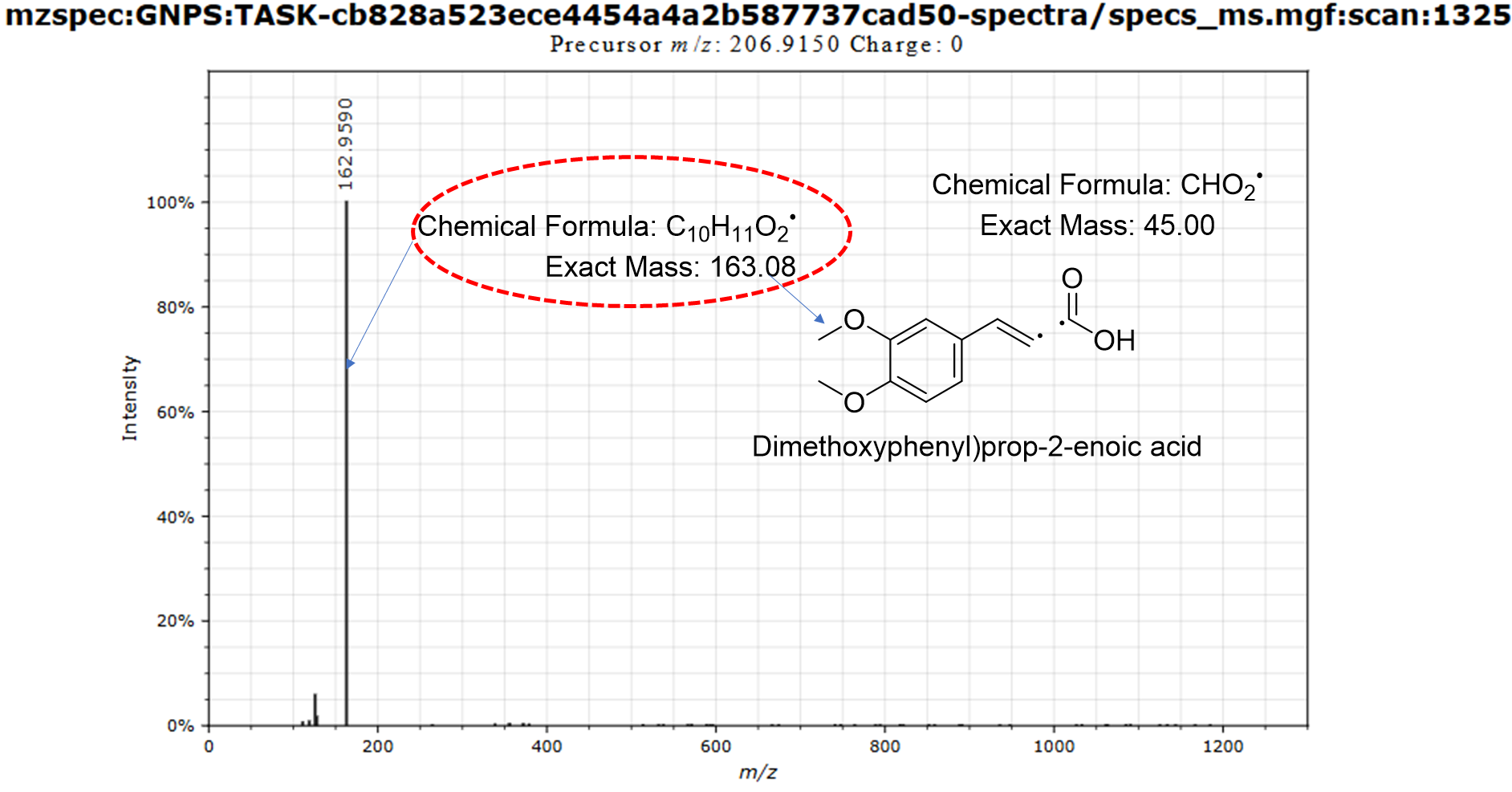


Supplementary Figure 6. LC-MS/MS of dimethoxycinnamic acid


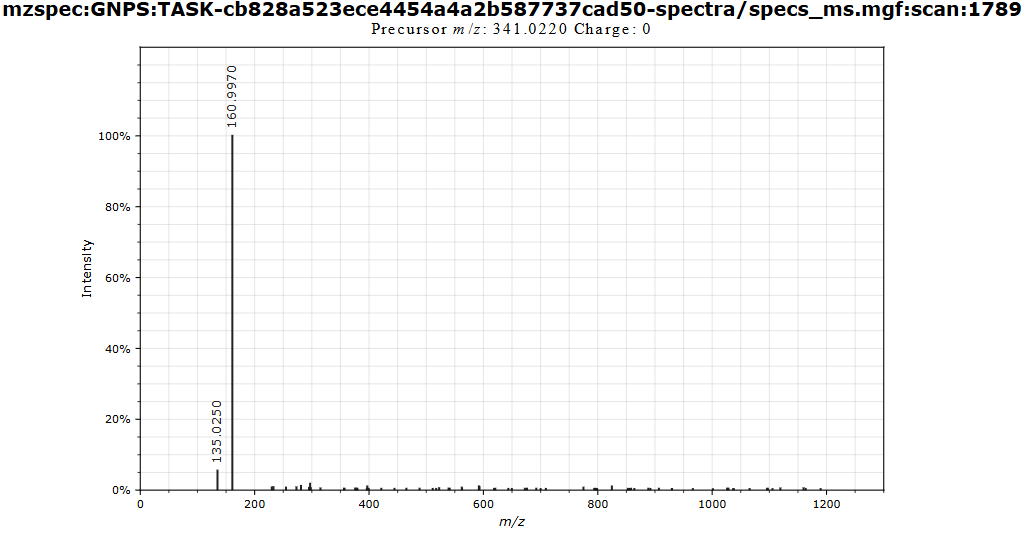


Supplementary Figure 7. LC-MS/MS of caffeic acid-glucoside


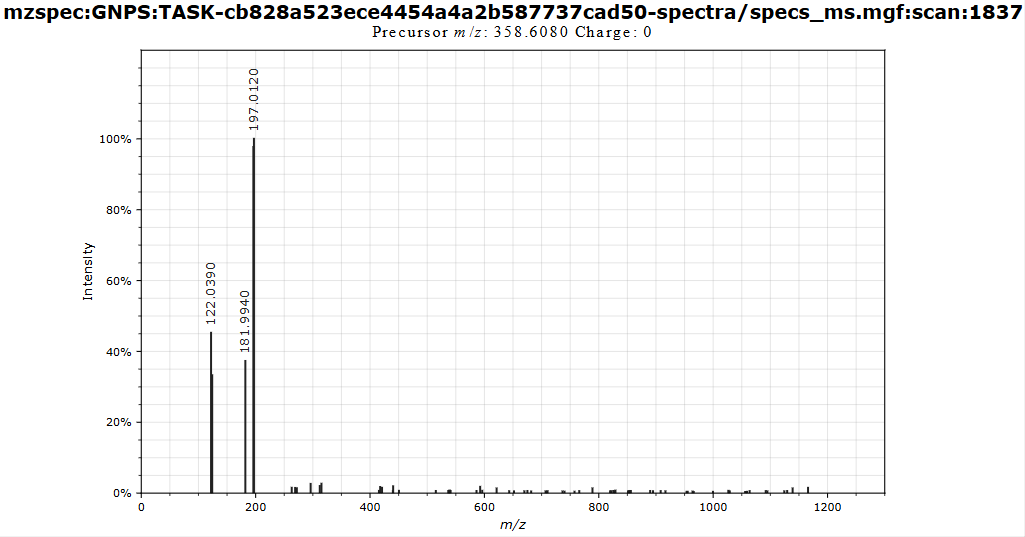


Supplementary Figure 8. LC-MS/MS of rosmarinic acid


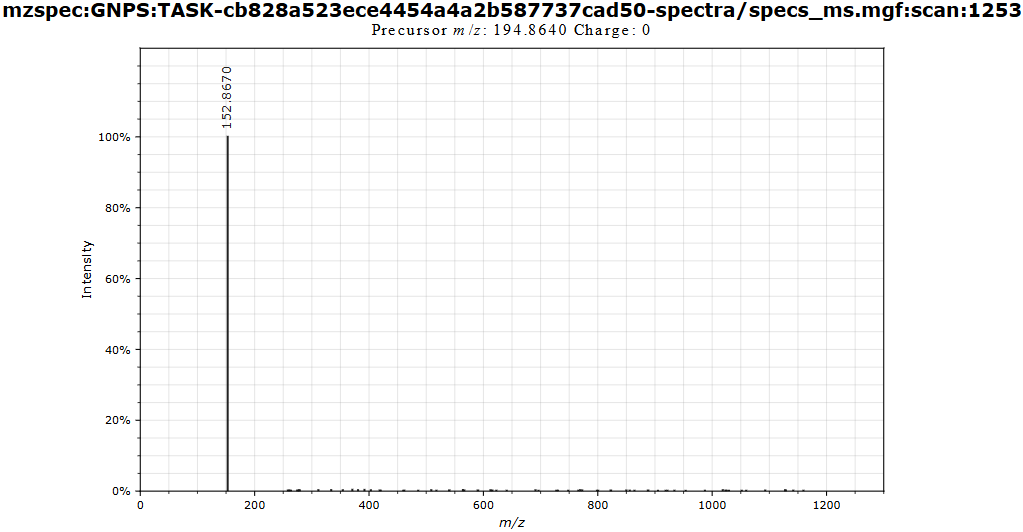


Supplementary Figure 9. LC-MS/MS of dihydroferulic acid


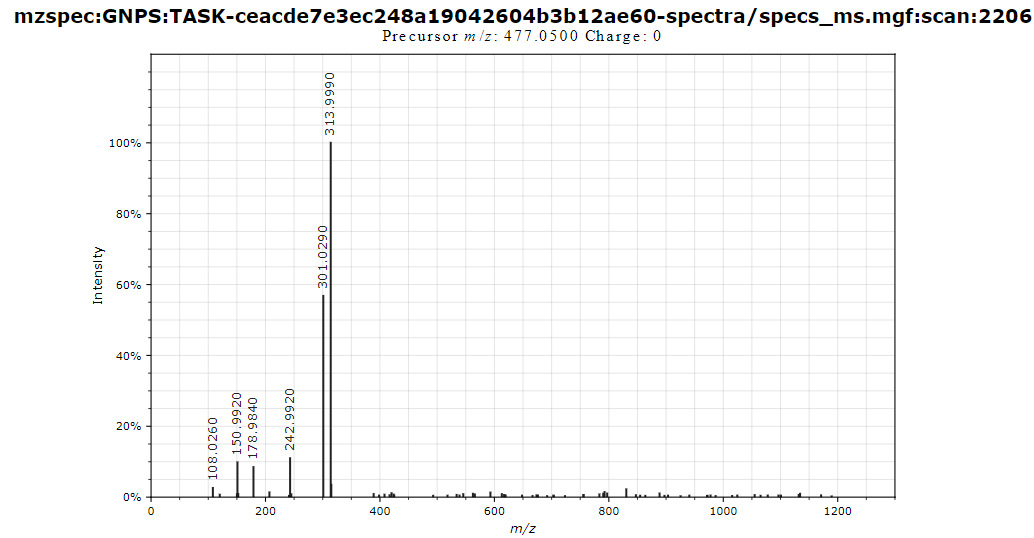


Supplementary Figure 10. LC-MS/MS of quercetin-3-O-glucuronide


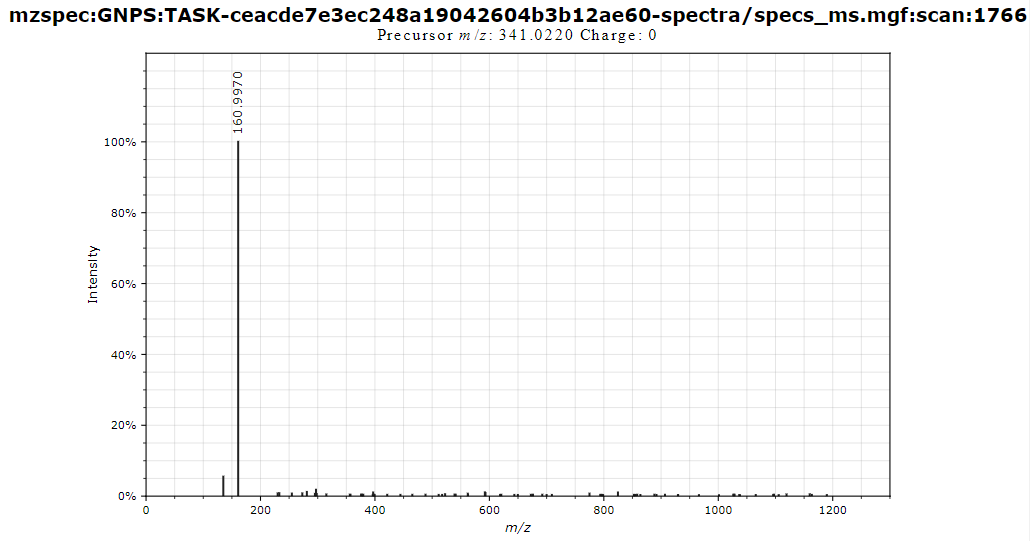


Supplementary Figure 11. LC-MS/MS of aesculin


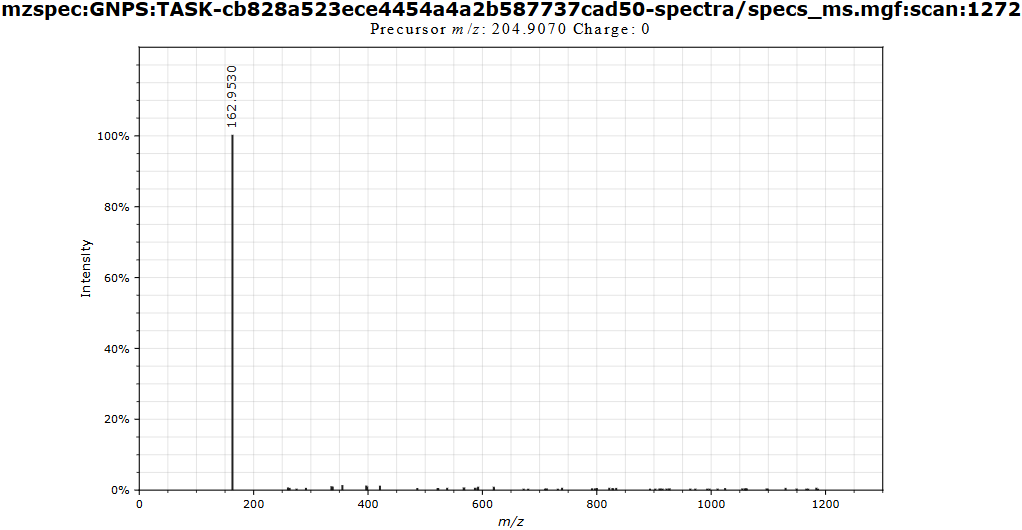


Supplementary Figure 12. LC-MS-MS of vinyl caffeate


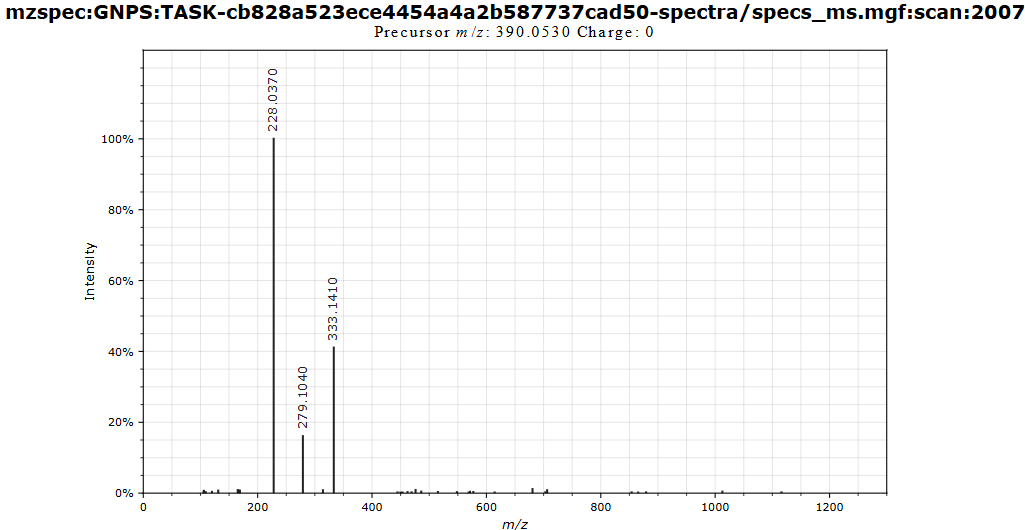


Supplementary Figure 13. LC-MS/MS of polydatin


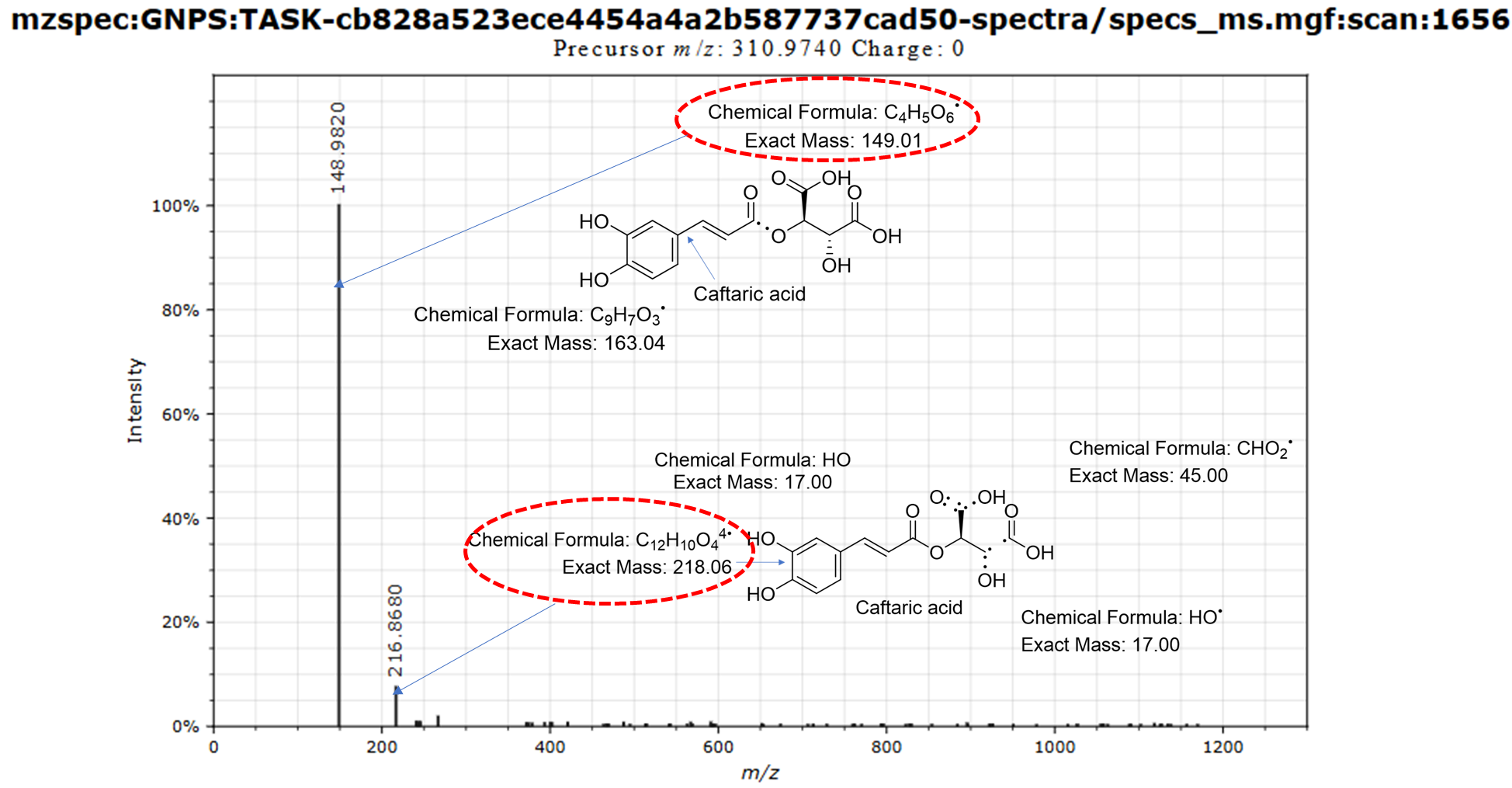


Supplementary Figure 14. LC-MS/MS of caftaric acid


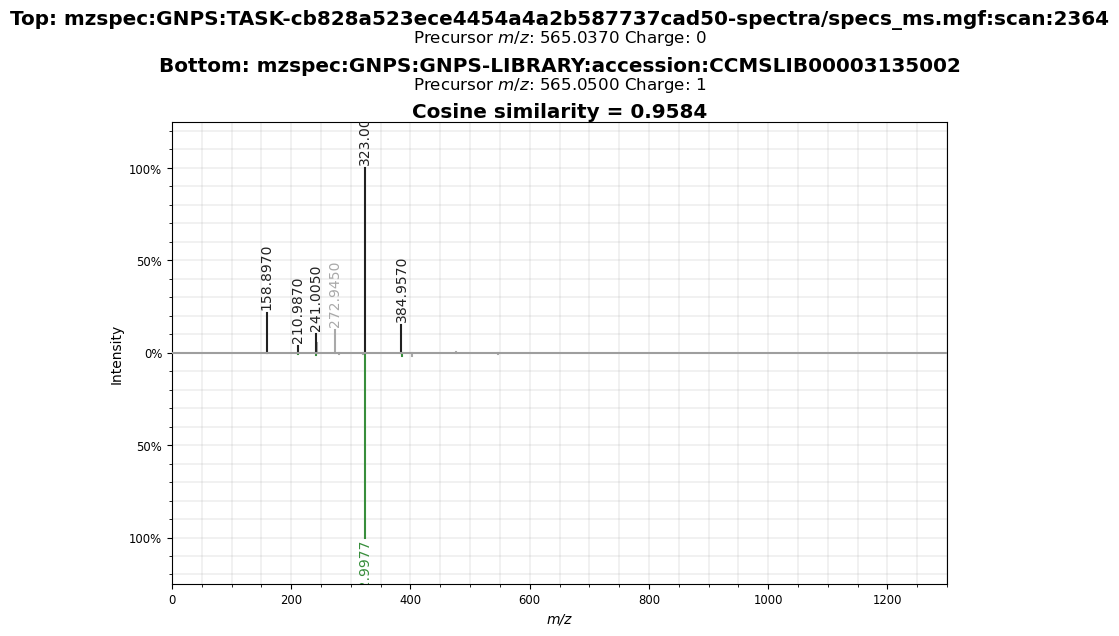


Supplementary Figure 15. Mirror plot image for the matching between the raw mass spectrum fragments and that of the GNPS library for uridine 5'-diphosphogalactose. The upper fragments from the raw MS data file and the lower ones from the GNPS library.


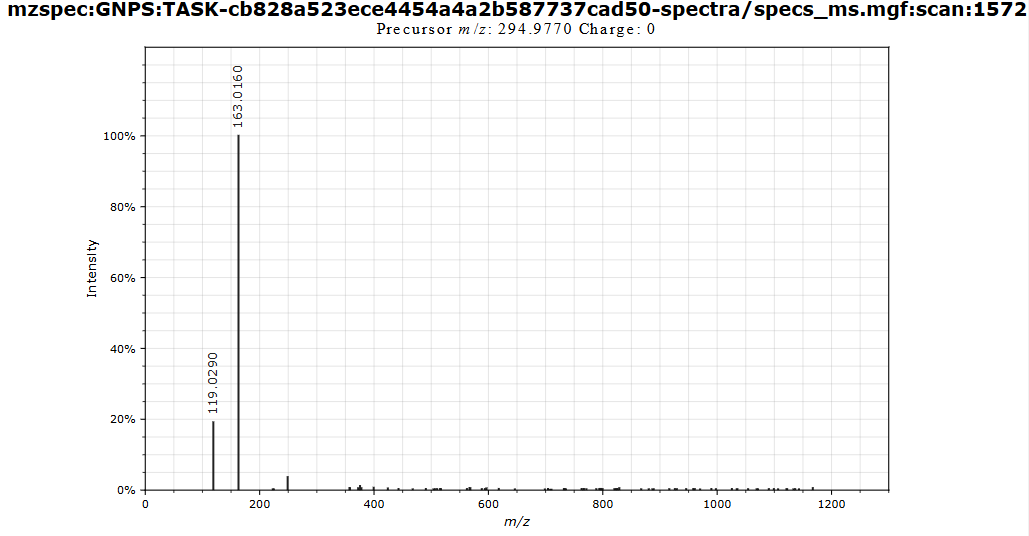


Supplementary Figure 16. LC-MS/MS of coutaric acid


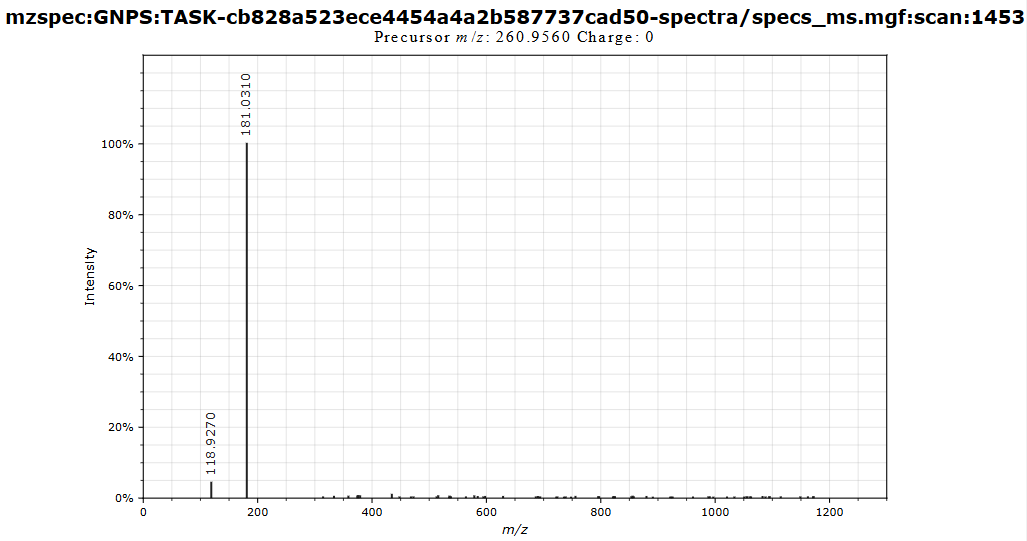


Supplementary Figure 17. LC-MS/MS of sinapinic acid


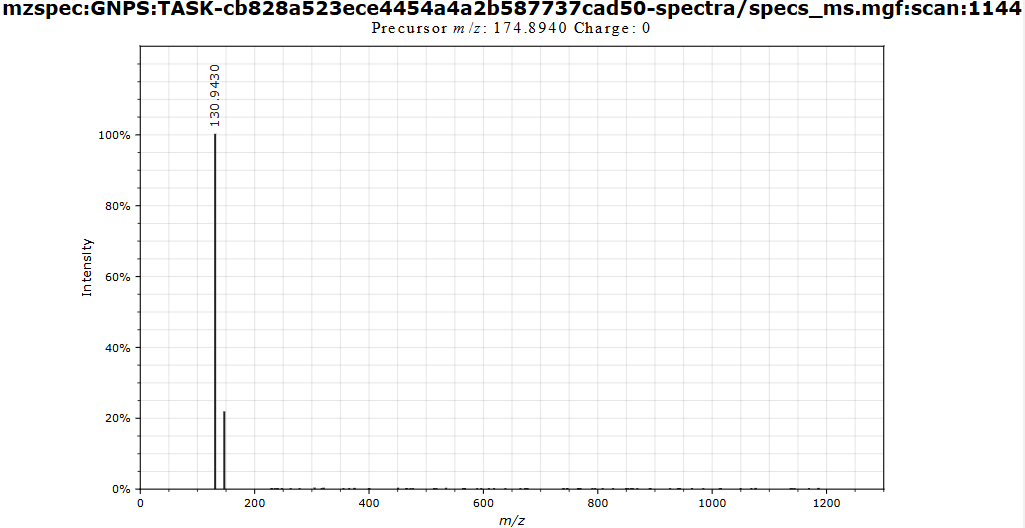


Supplementary Figure 18. LC-MS/MS of indole-3-acetic acid


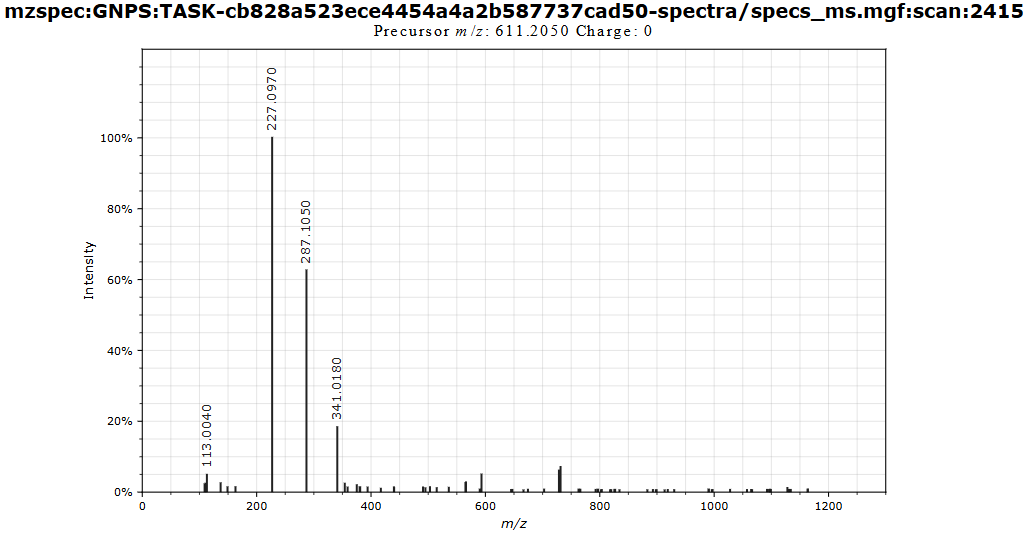


Supplementary Figure 19. LC-MS/MS of kaempferol diglycoside


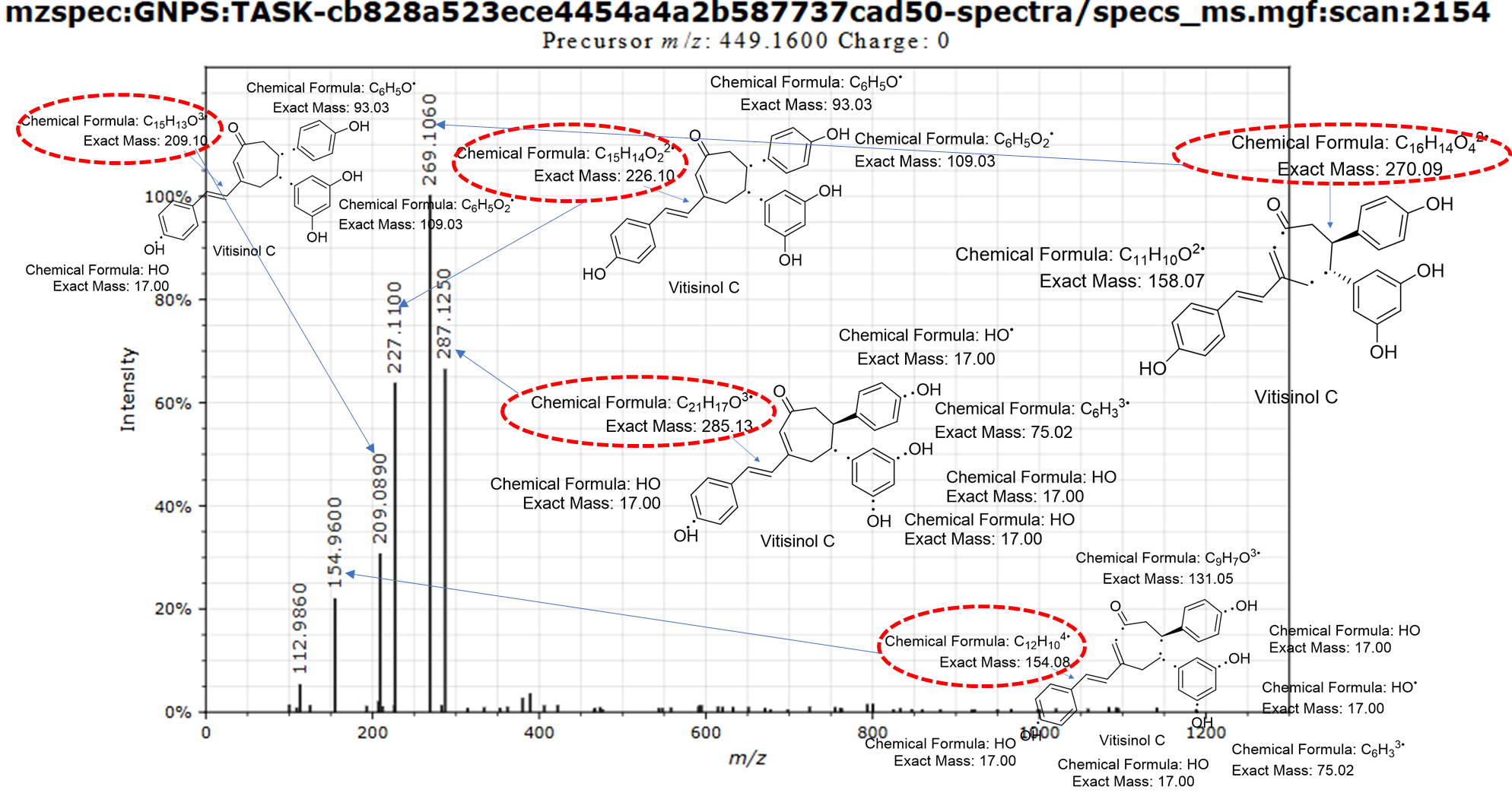


Supplementary Figure 20. LC-MS/MS of vitisinol C


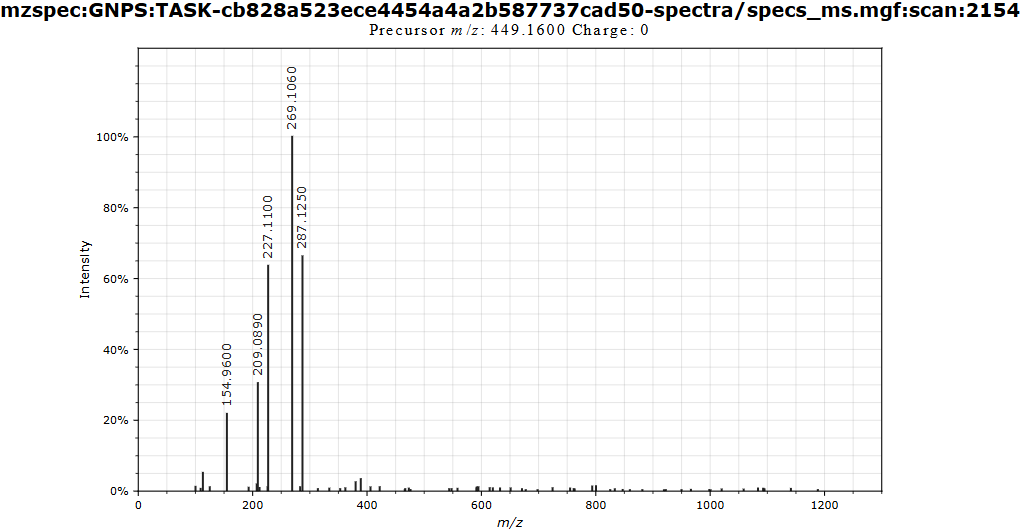


Supplementary Figure 21. LC-MS/MS of dihydrokaempferol glucoside


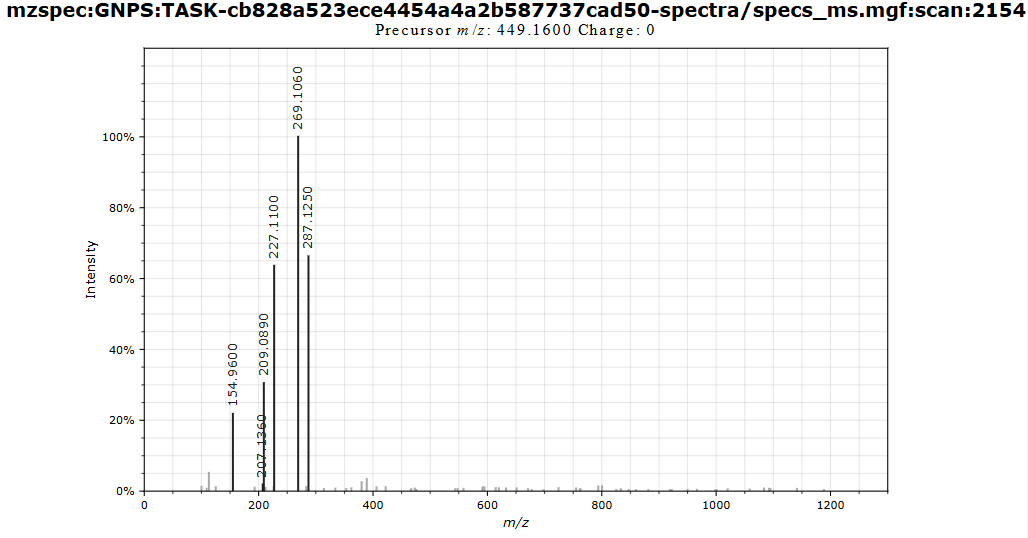


Supplementary Figure 22. LC-MS/MS cyanidin-3-O-glucoside


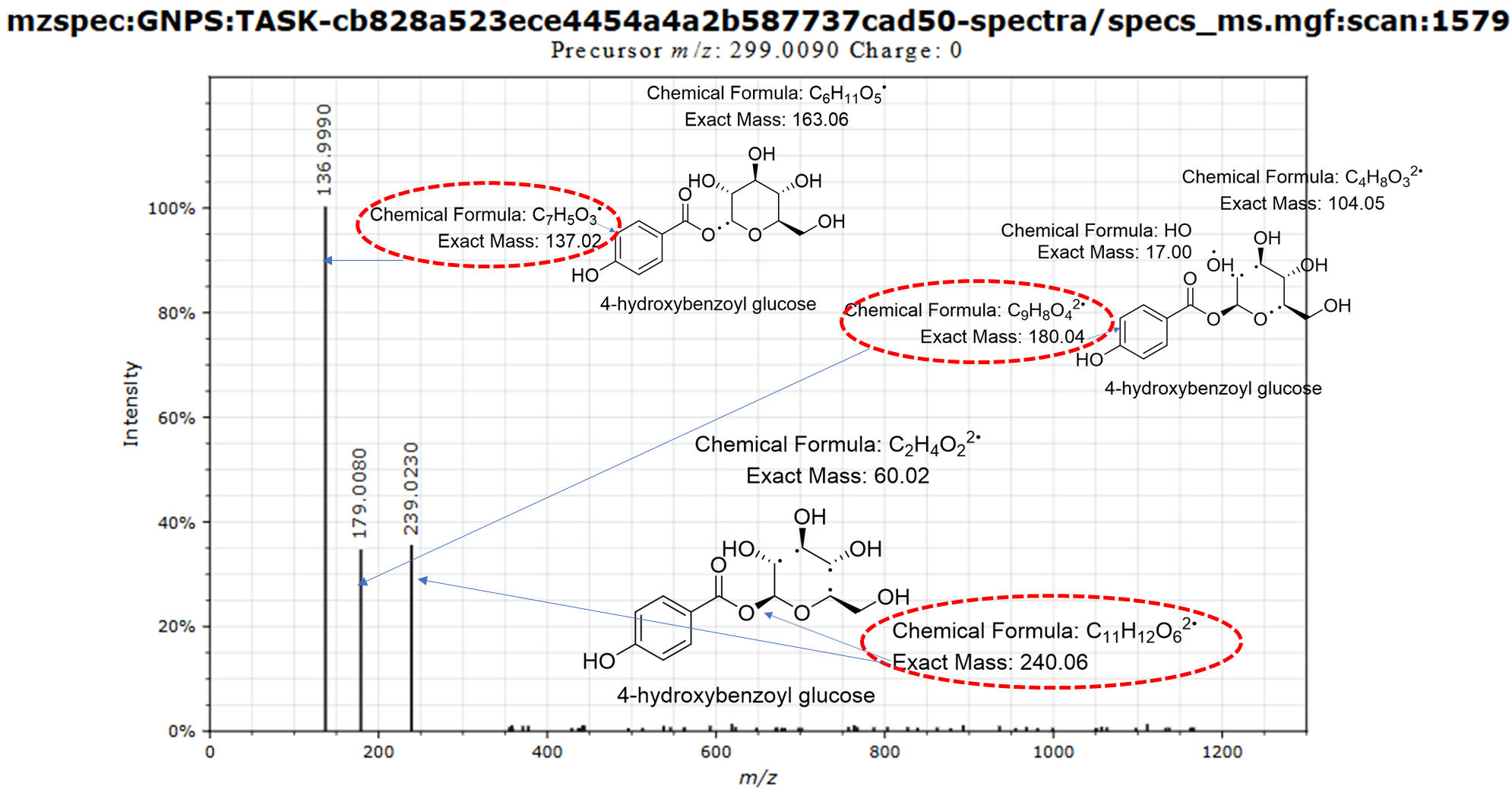


Supplementary Figure 23. LC-MS/MS of 4-hydroxybenzoyl glucose


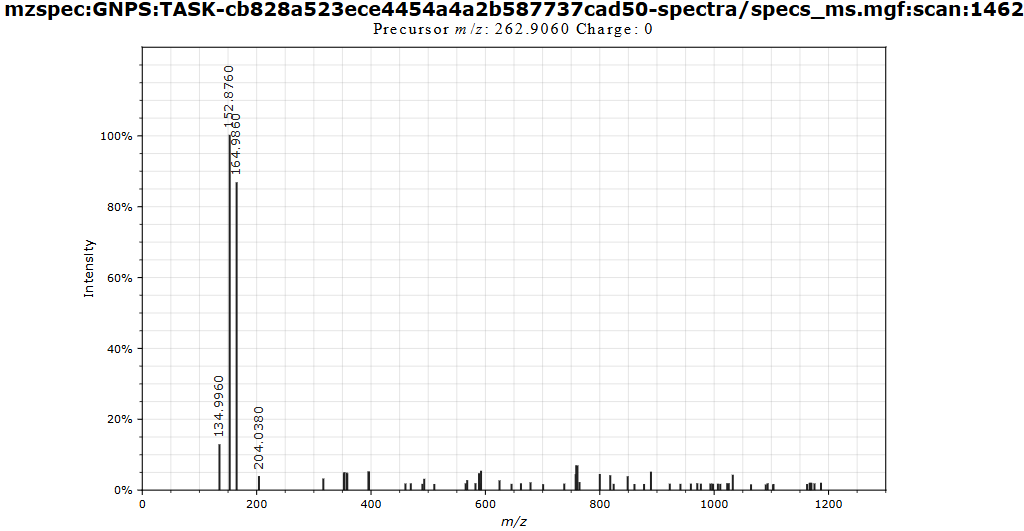


Supplementary Figure 24. LC-MS/MS of abscisic acid


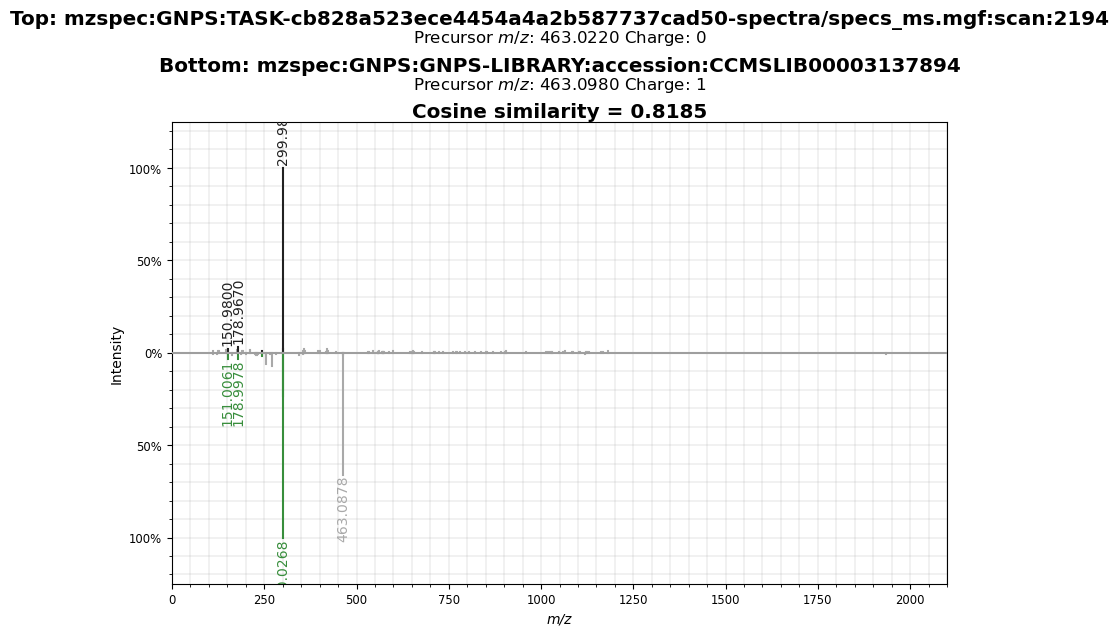


Supplementary Figure 25. Mirror plot image for the matching between the raw mass spectrum fragments and that of the GNPS library for spiraeoside. The upper fragments from the raw MS data file and the lower ones from the GNPS library.


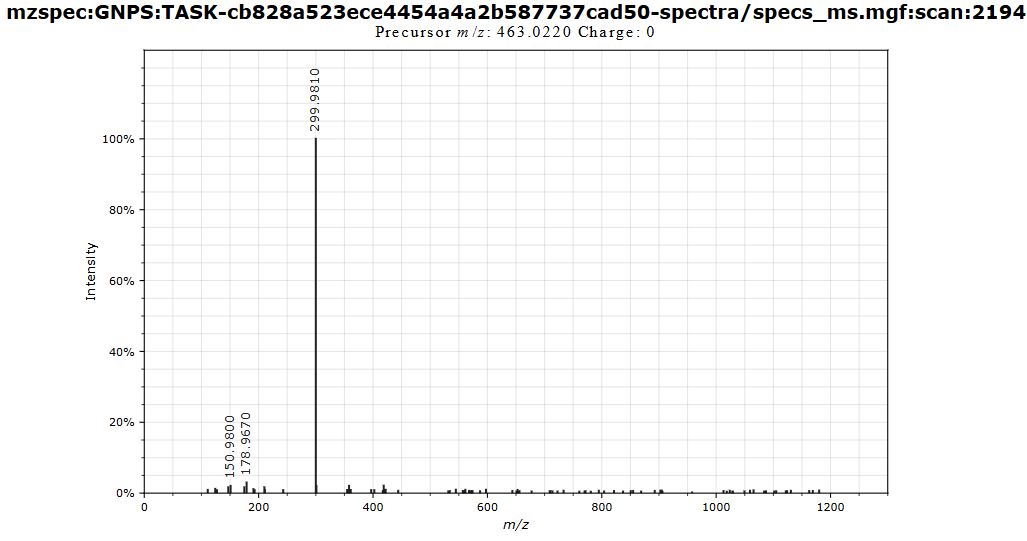


Supplementary Figure 26. LC-MS/MS of quercetin 3-O-galactoside; hyperoside


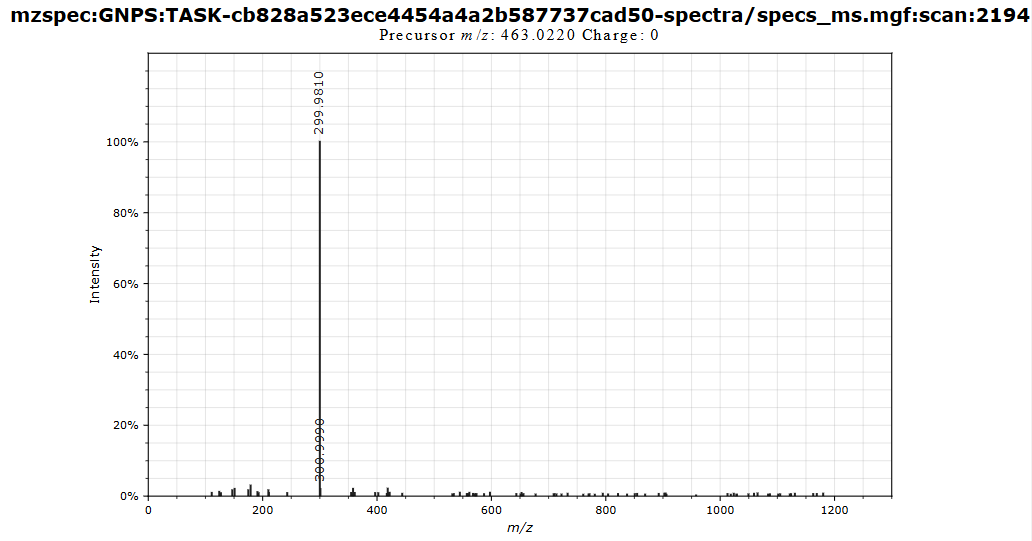


Supplementary Figure 27. LC-MS/MS of peonidin-3-O-glucoside


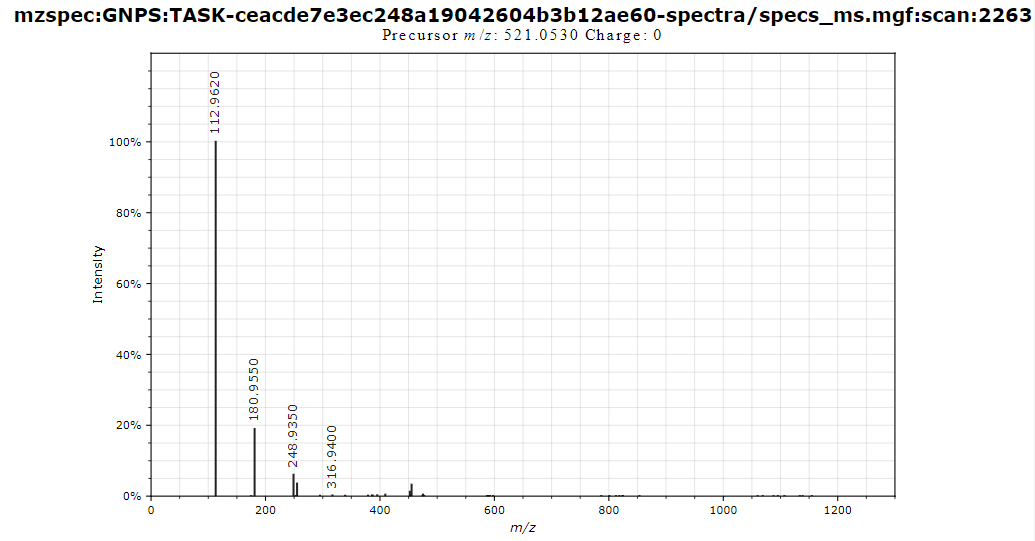


Supplementary Figure 28. LC-MS/MS of petunidin 3-(6''-acetylglucoside)


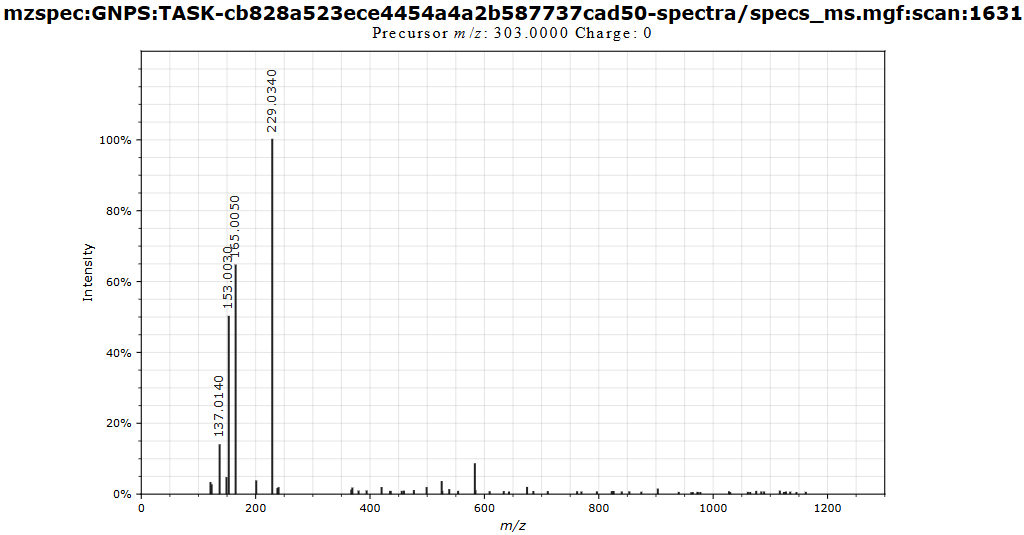


Supplementary Figure 29. LC-MS/MS of pentahydroxyflavone


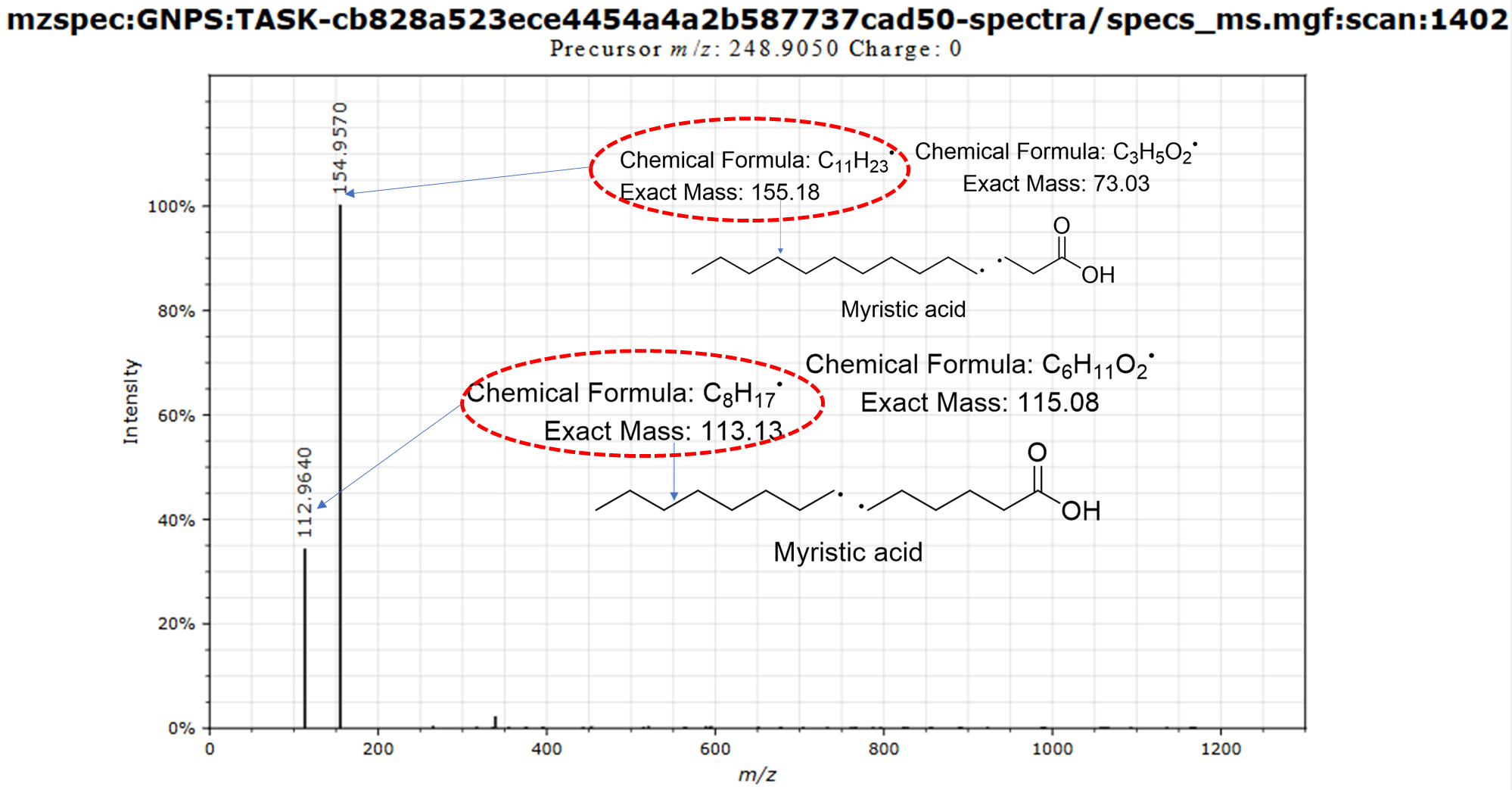


Supplementary Figure 30. LC-MS/MS of myristic acid (tetradecanoic acid)


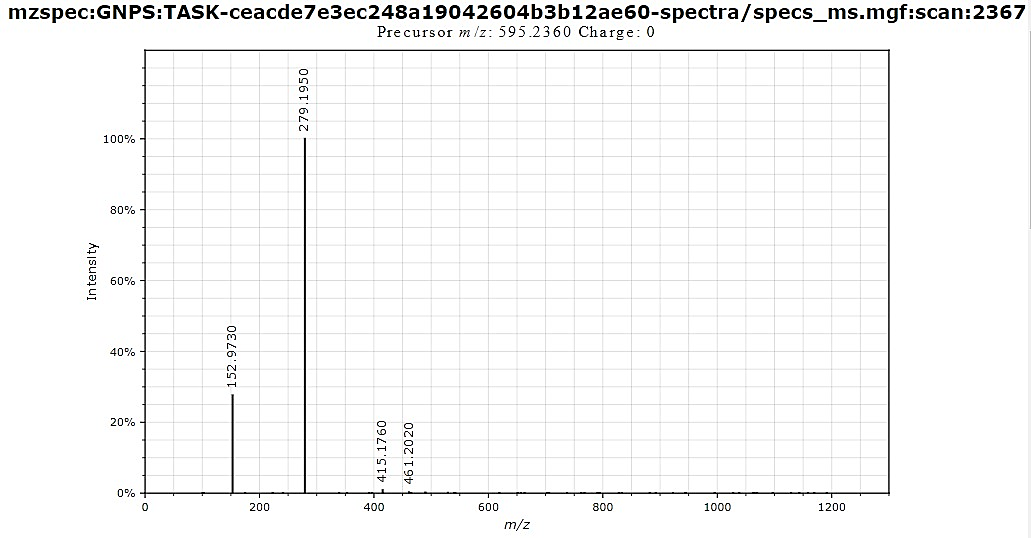

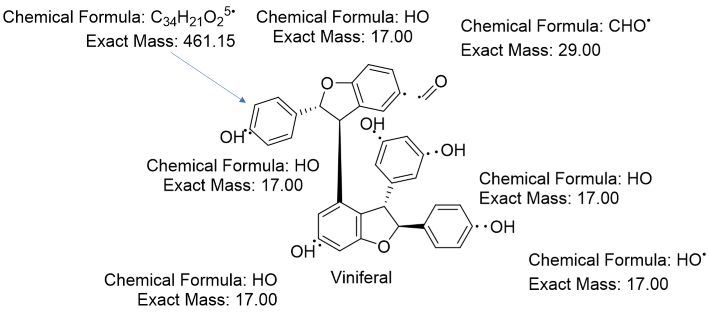

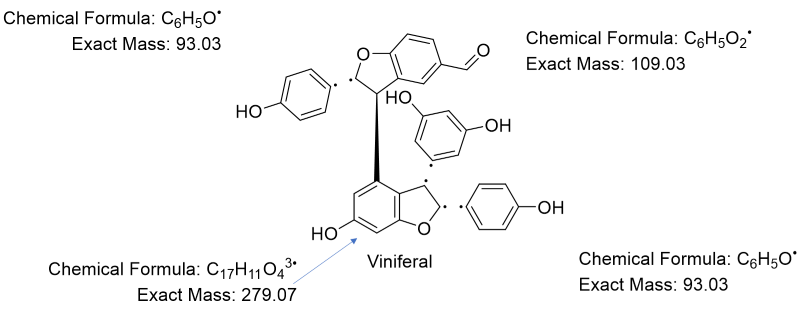

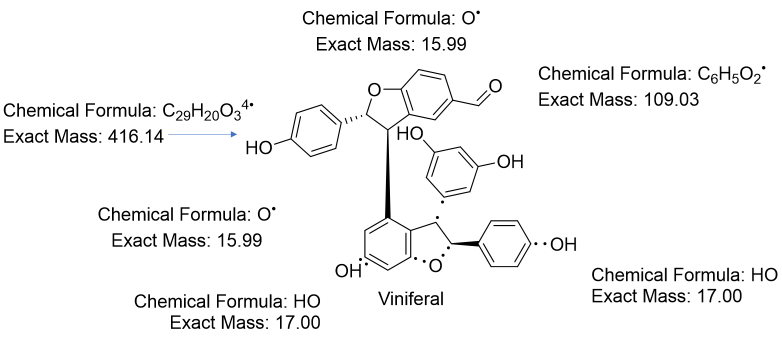

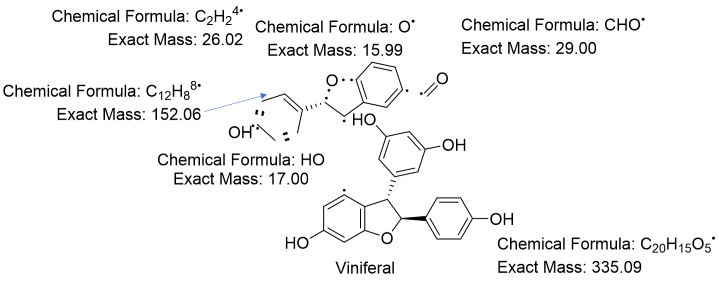


Supplementary Figure 31. LC-MS/MS of viniferal


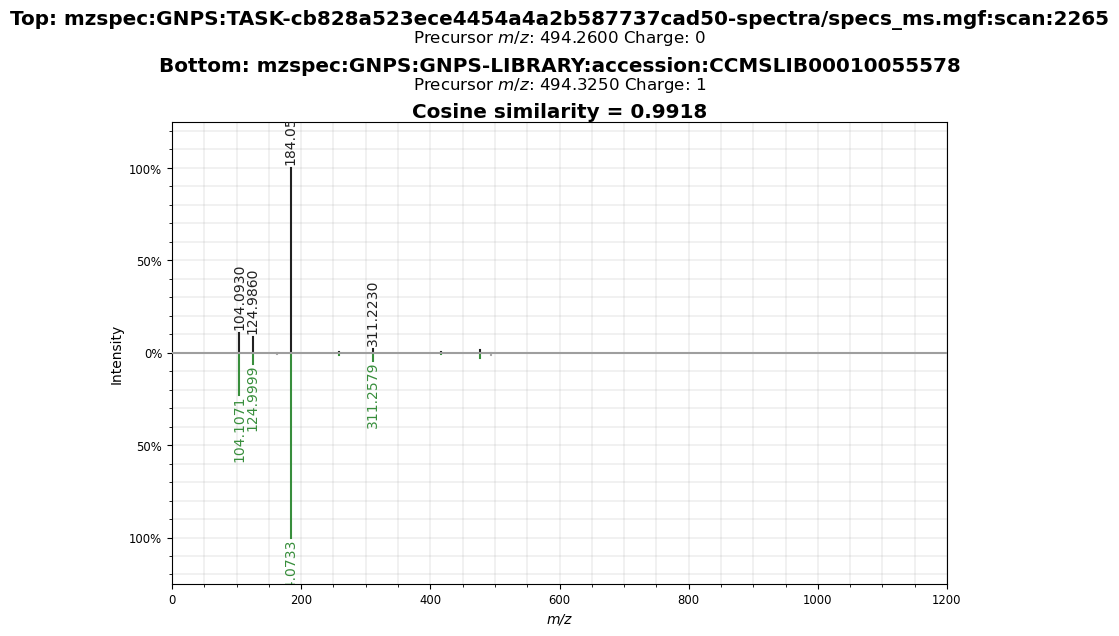


Supplementary Figure 32. Mirror plot image for the matching between the raw mass spectrum fragments and that of the GNPS library for PC(16:1/0:0). The upper fragments from the raw MS data file and the lower ones from the GNPS library.


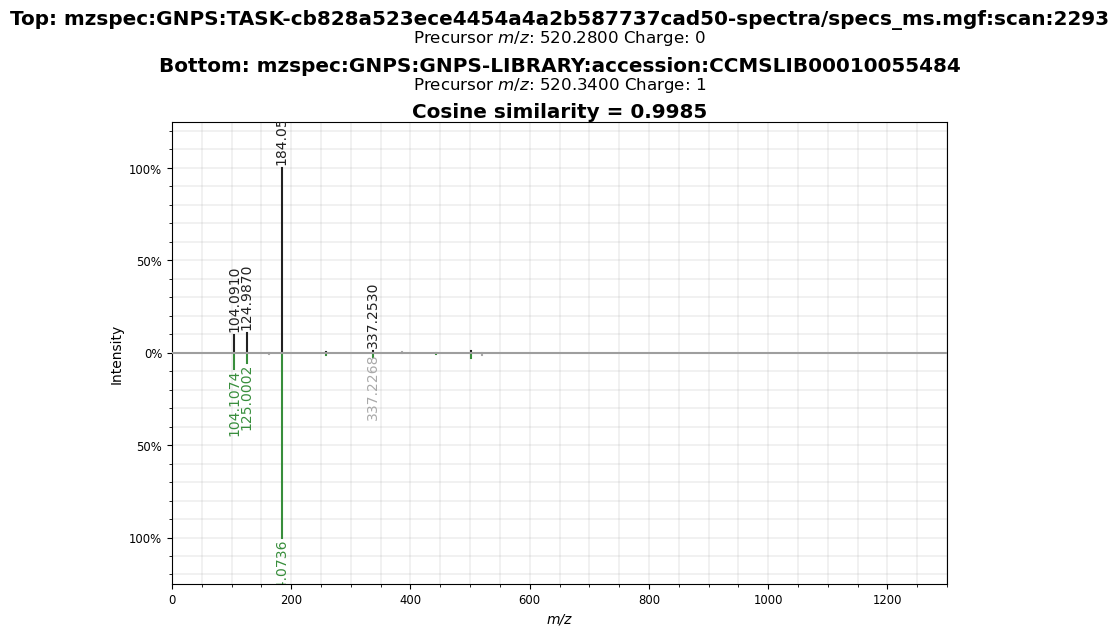


Supplementary Figure 33. Mirror plot image for the matching between the raw mass spectrum fragments and that of the GNPS library for PC(18:2/0:0). The upper fragments from the raw MS data file and the lower ones from the GNPS library.


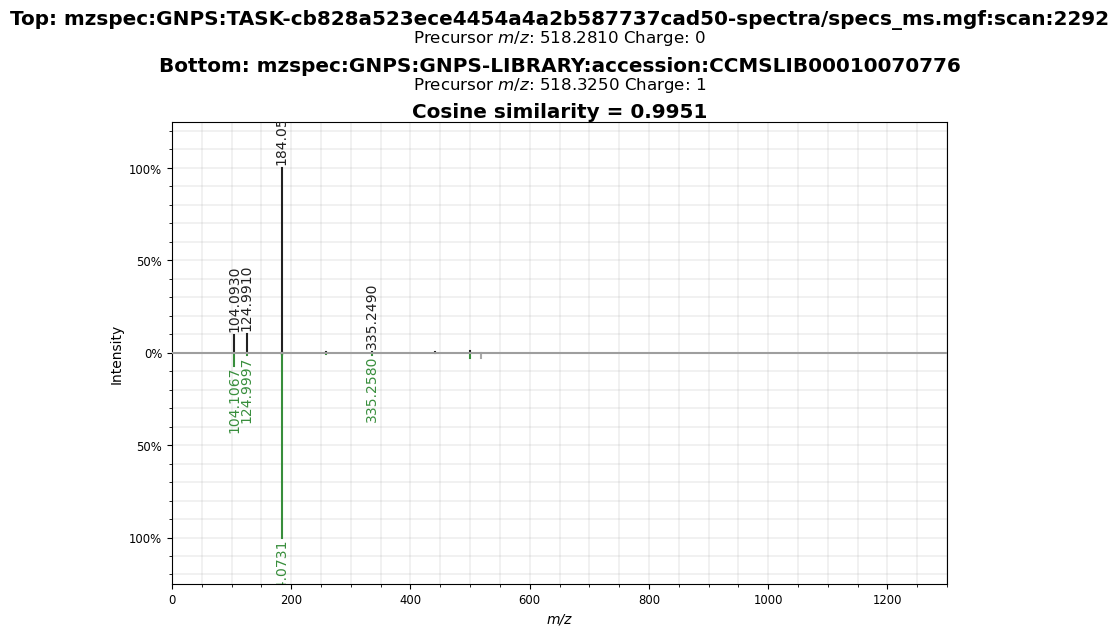


Supplementary Figure 34. Mirror plot image for the matching between the raw mass spectrum fragments and that of the GNPS library for PC(18:3/0:0). The upper fragments from the raw MS data file and the lower ones from the GNPS library.


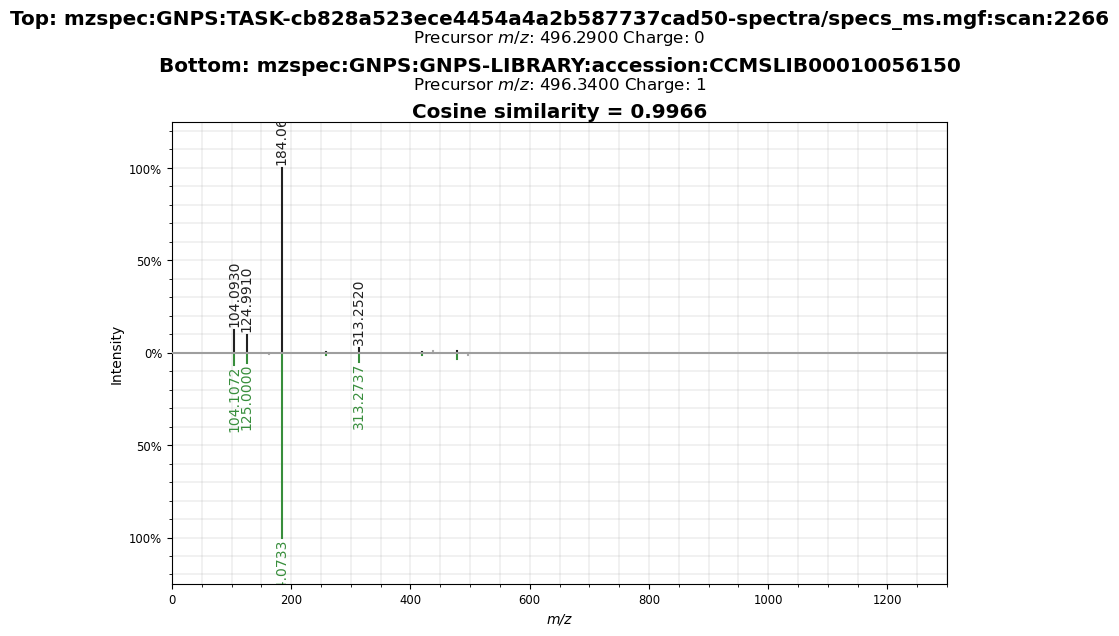


Supplementary Figure 35. Mirror plot image for the matching between the raw mass spectrum fragments and that of the GNPS library for PC(0:0/16:0). The upper fragments from the raw MS data file and the lower ones from the GNPS library.


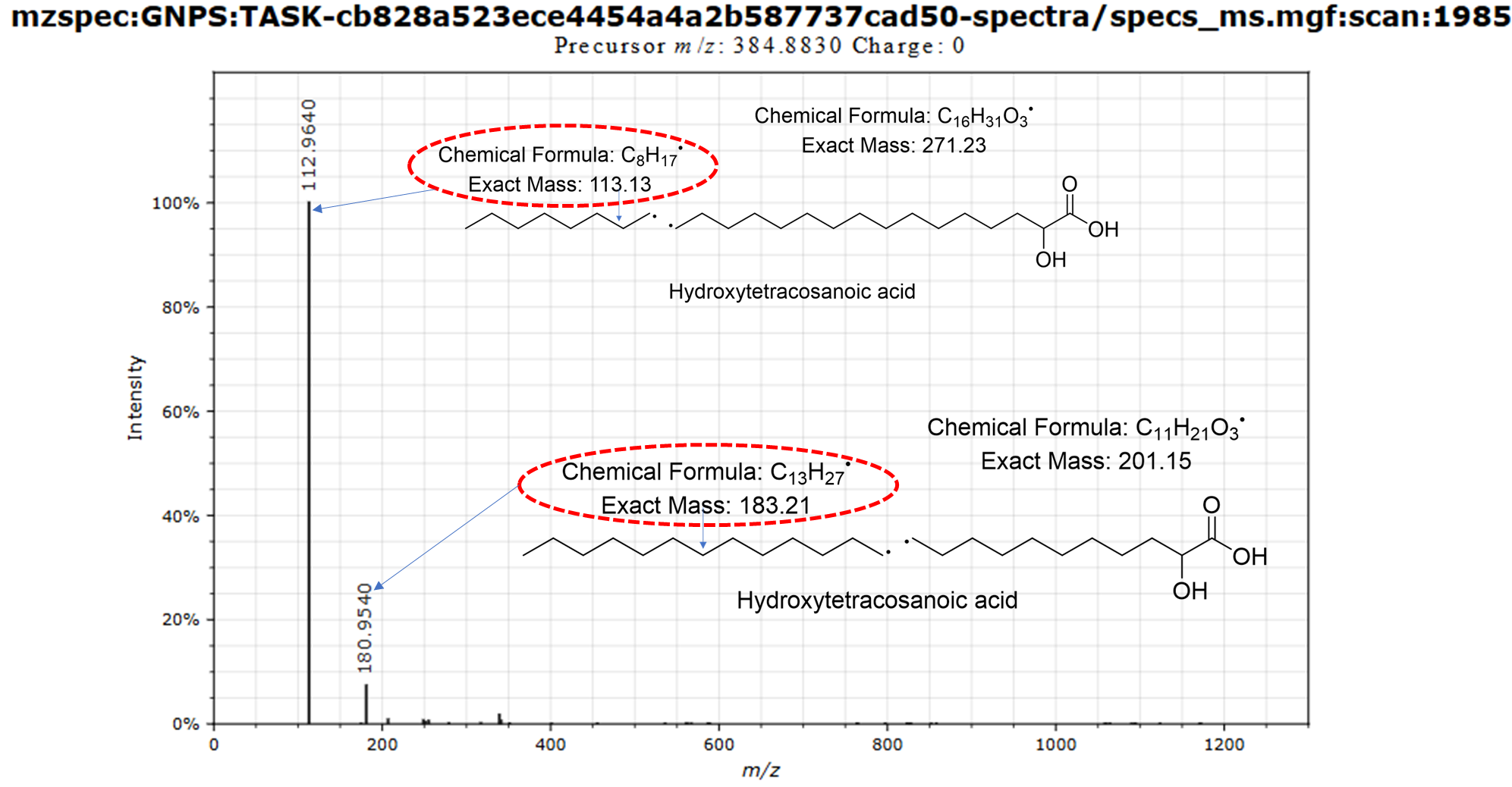


Supplementary Figure 36. LC-MS/MS of hydroxytetracosanoic acid


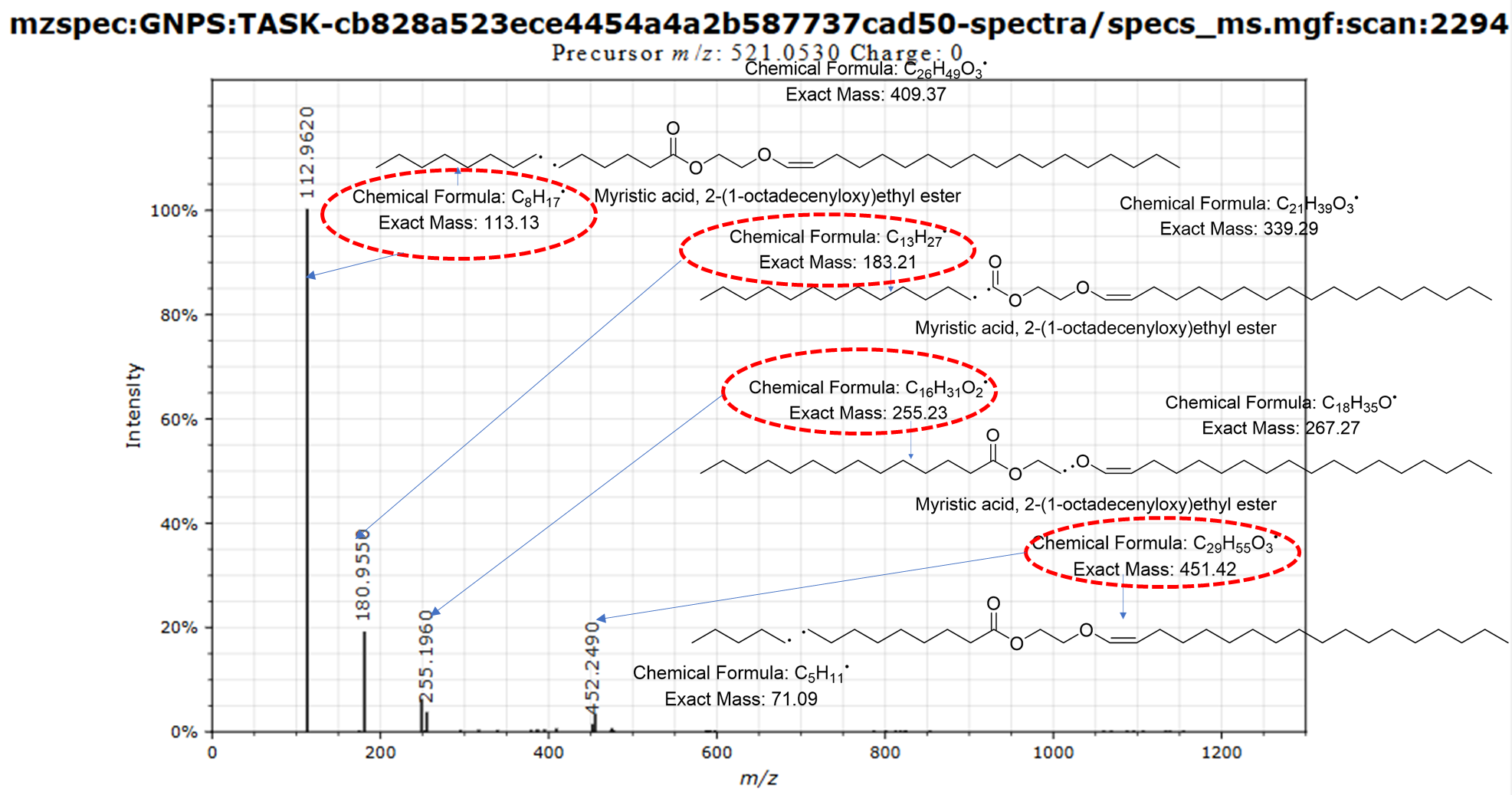


Supplementary Figure 37. LC-MS/MS of myristic acid, 2-(1-octadecenyloxy)ethyl ester


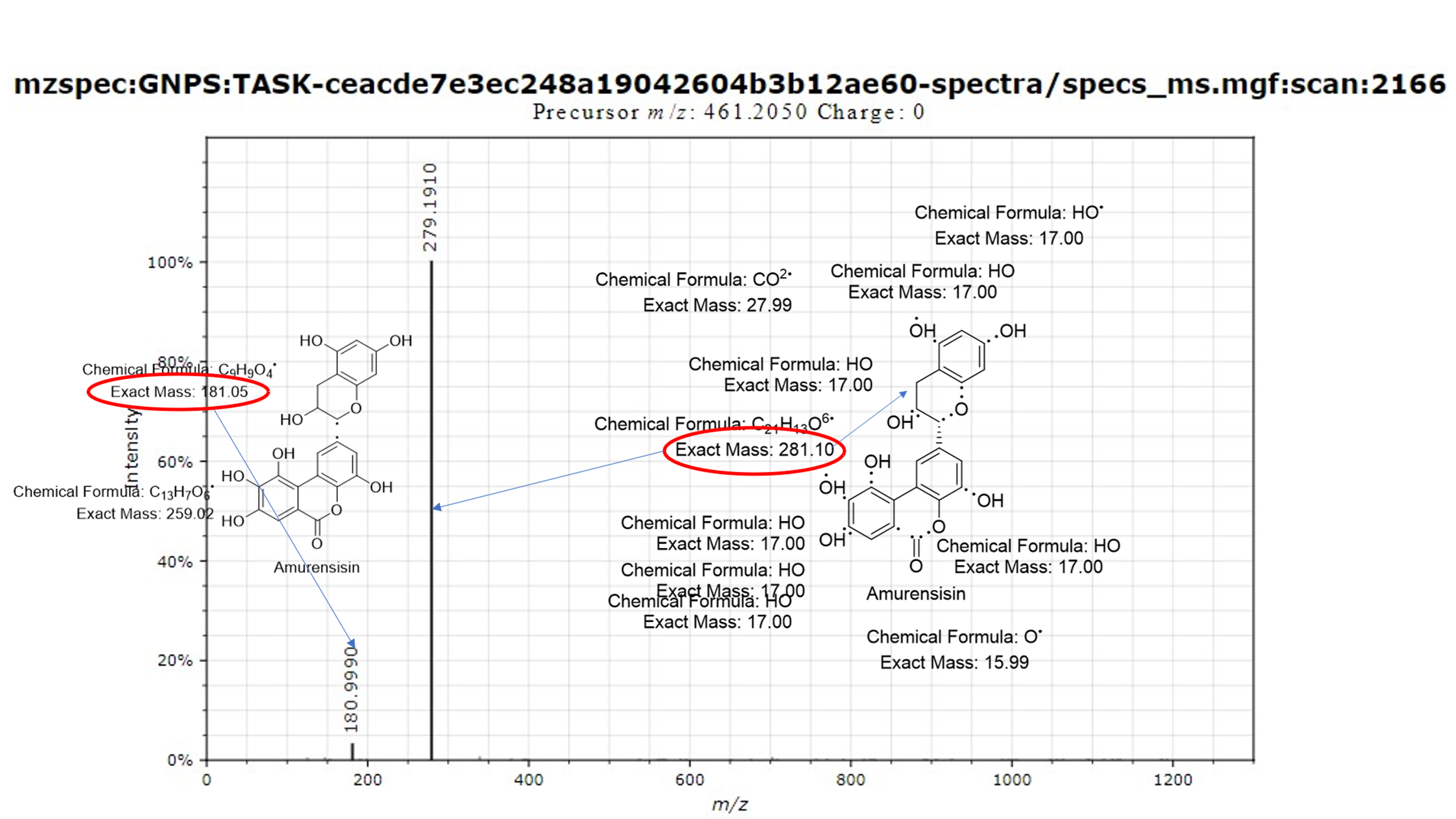


Supplementary Figure 38. LC-MS/MS of amurensisin


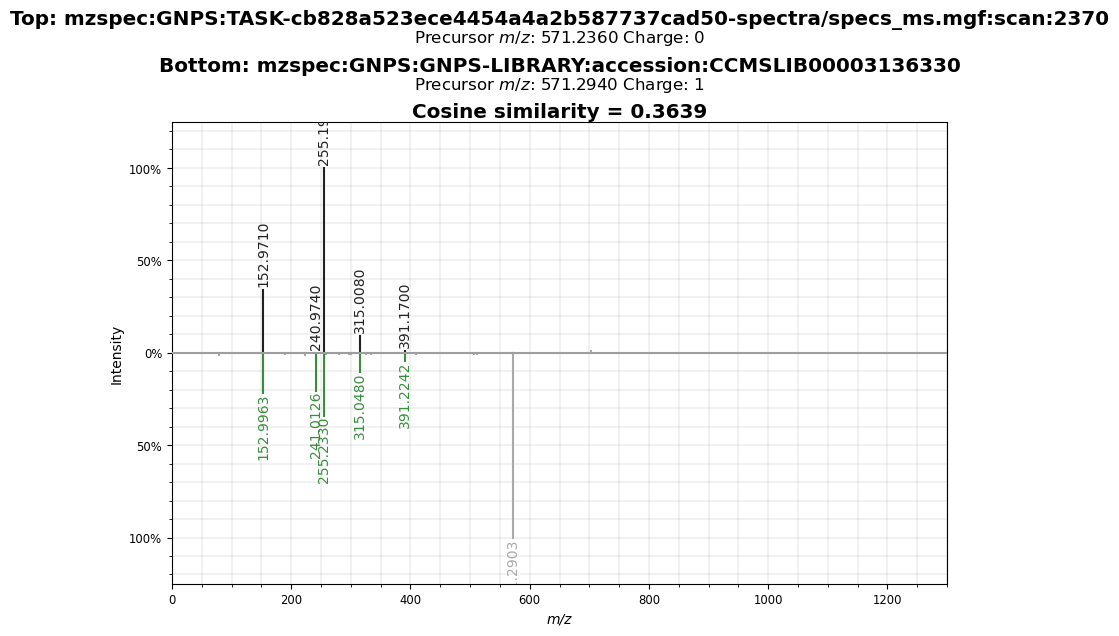


Supplementary Figure 39. Mirror plot image for the matching between the raw mass spectrum fragments and that of the GNPS library for PI(16:0/0:0). The upper fragments from the raw MS data file and the lower ones from the GNPS library.


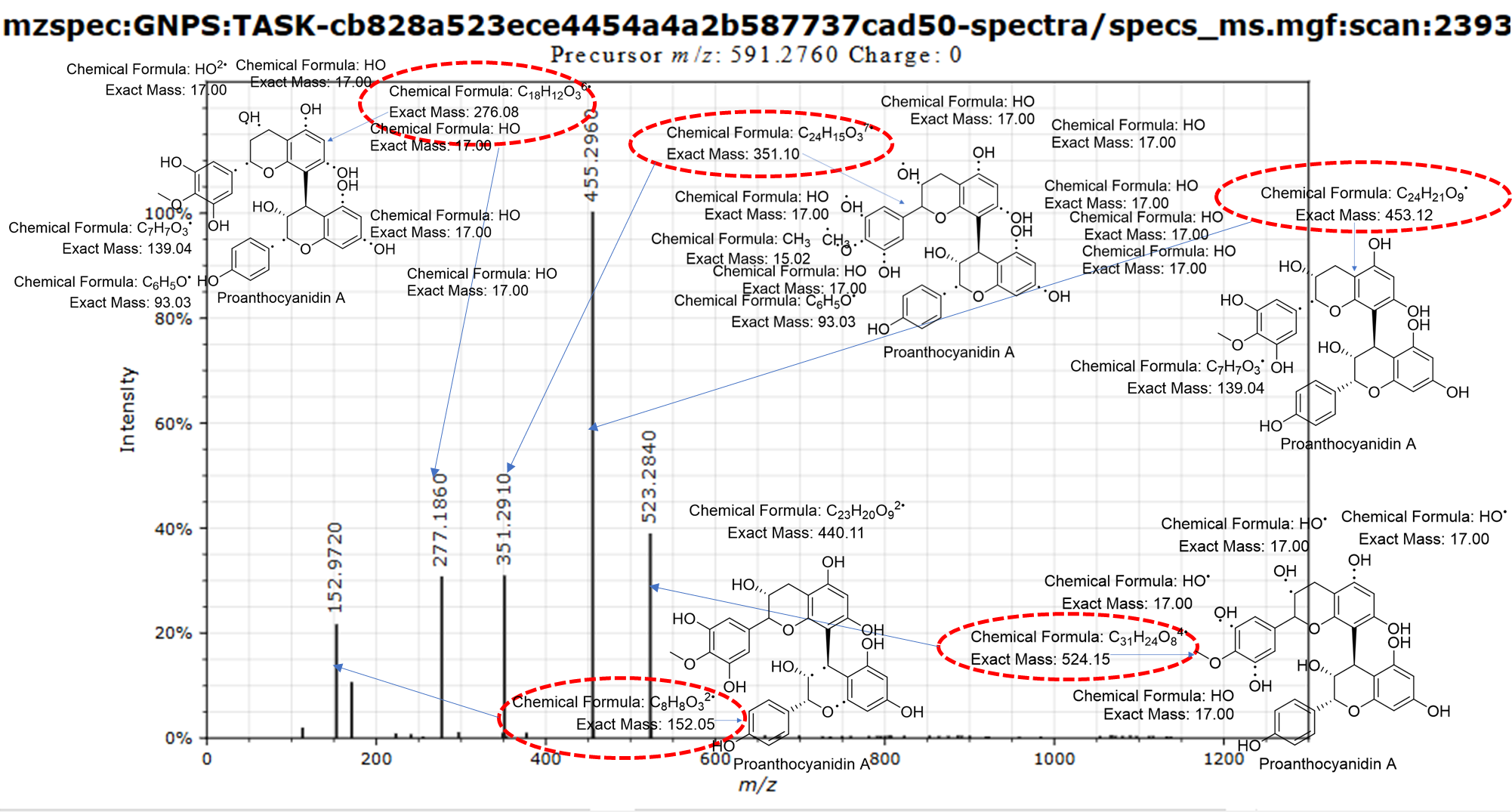


Supplementary Figure 40. LC-MS/MS of Proanthocyanidin A


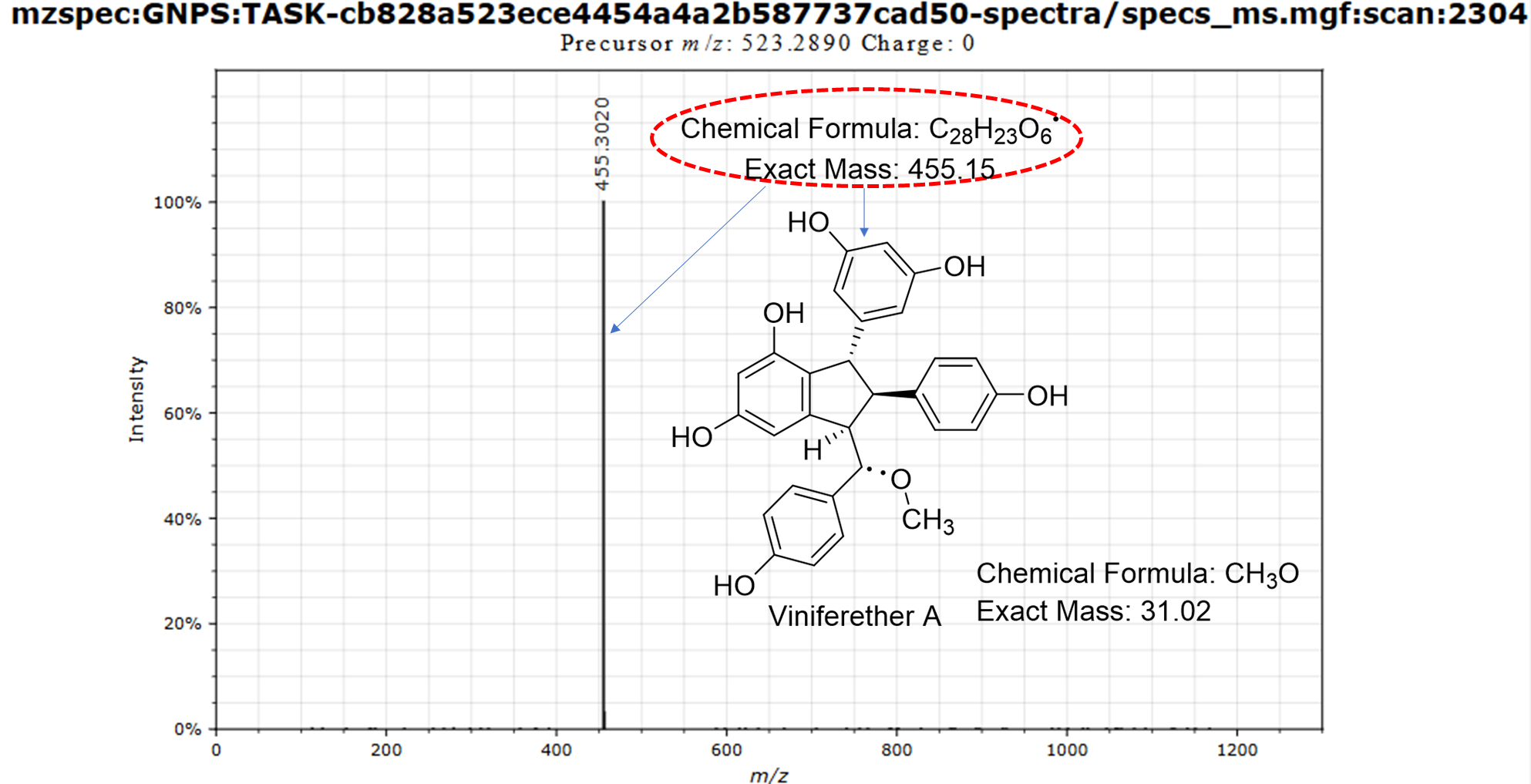


Supplementary Figure 41. LC-MS/MS of viniferether A


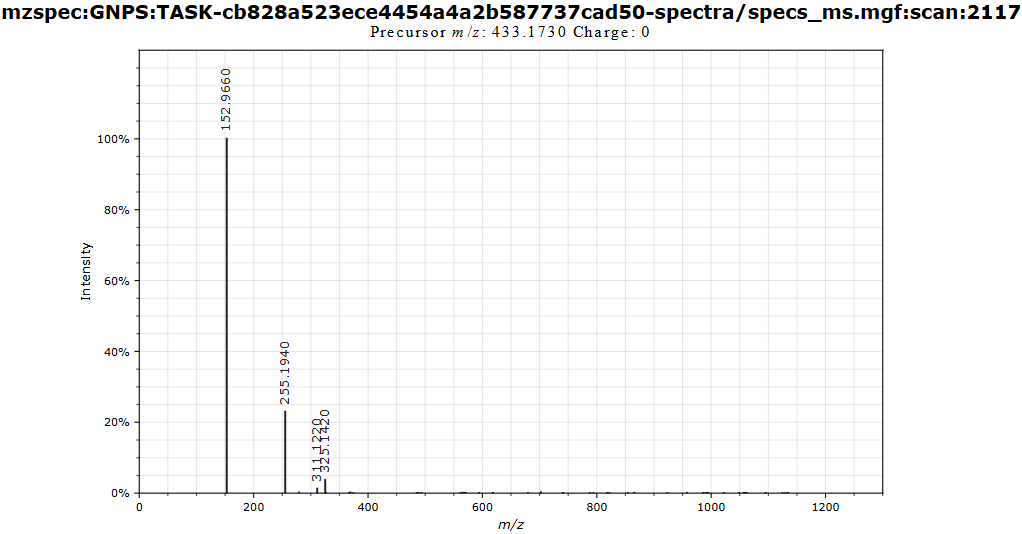


Supplementary Figure 42. LC-MS/MS of ergosterol


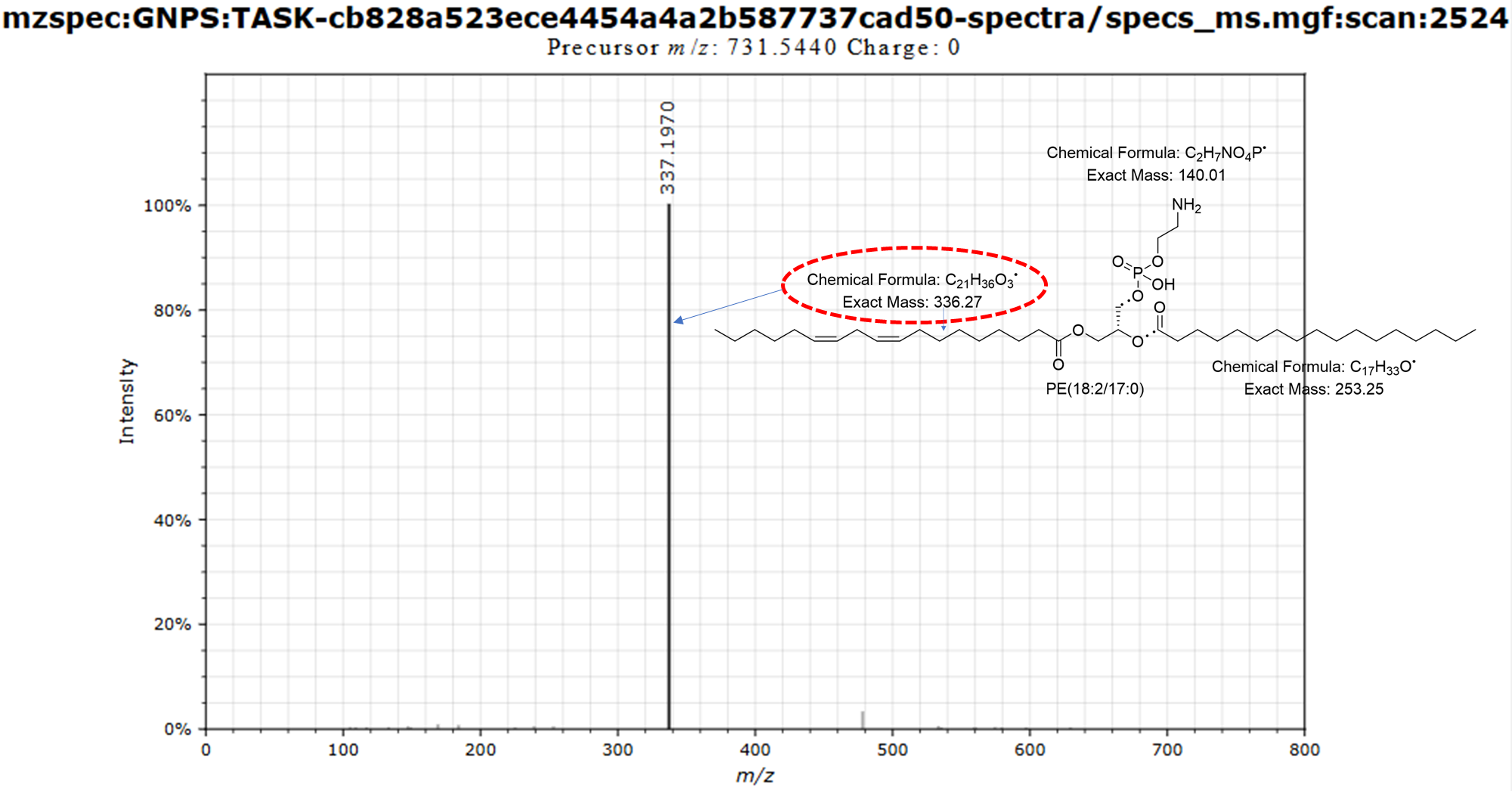


Supplementary Figure 43. LC-MS/MS of PE (18:2/17:0)


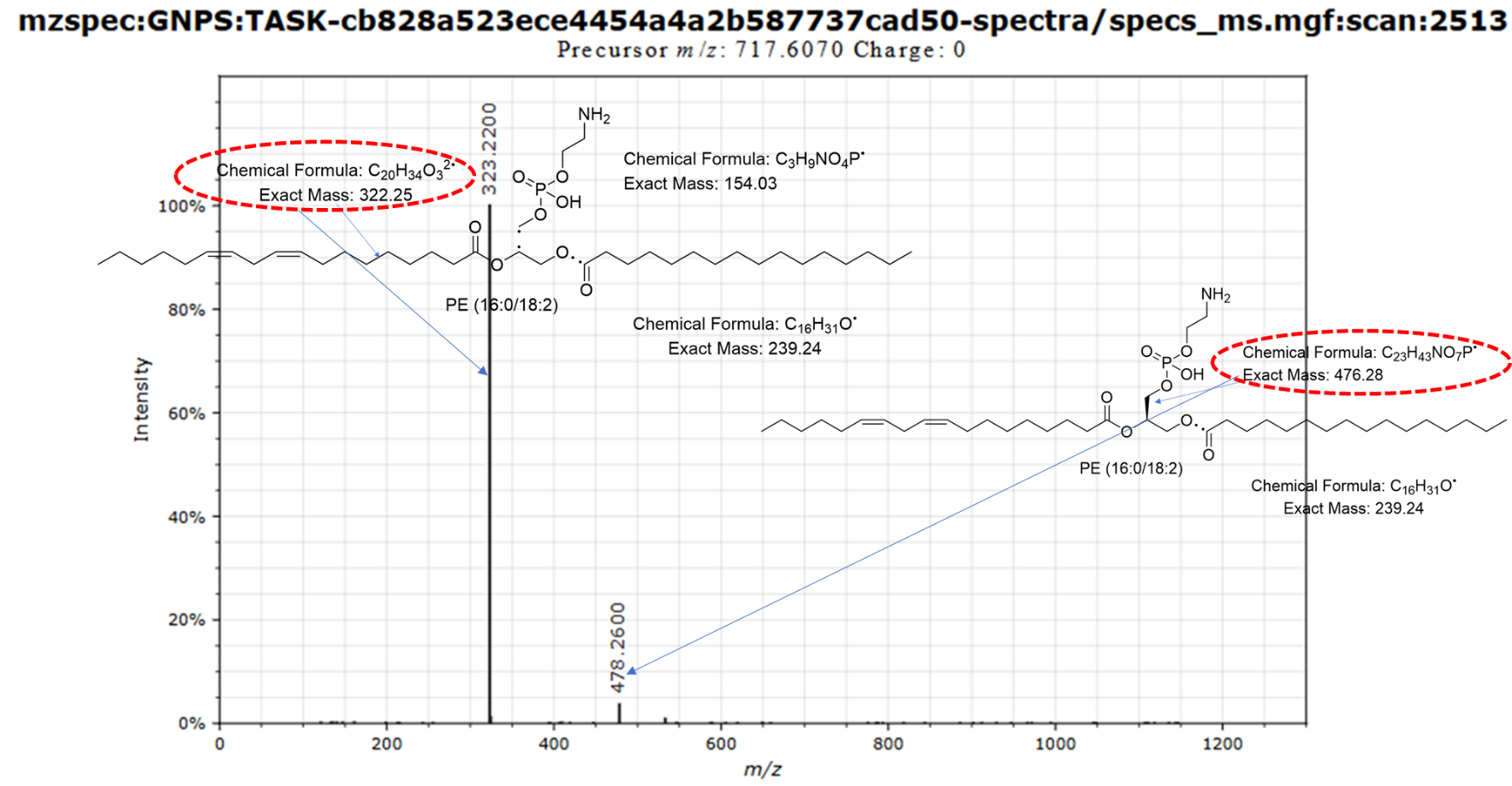


Supplementary Figure 44. LC-MS/MS of PE (16:0/18:2)


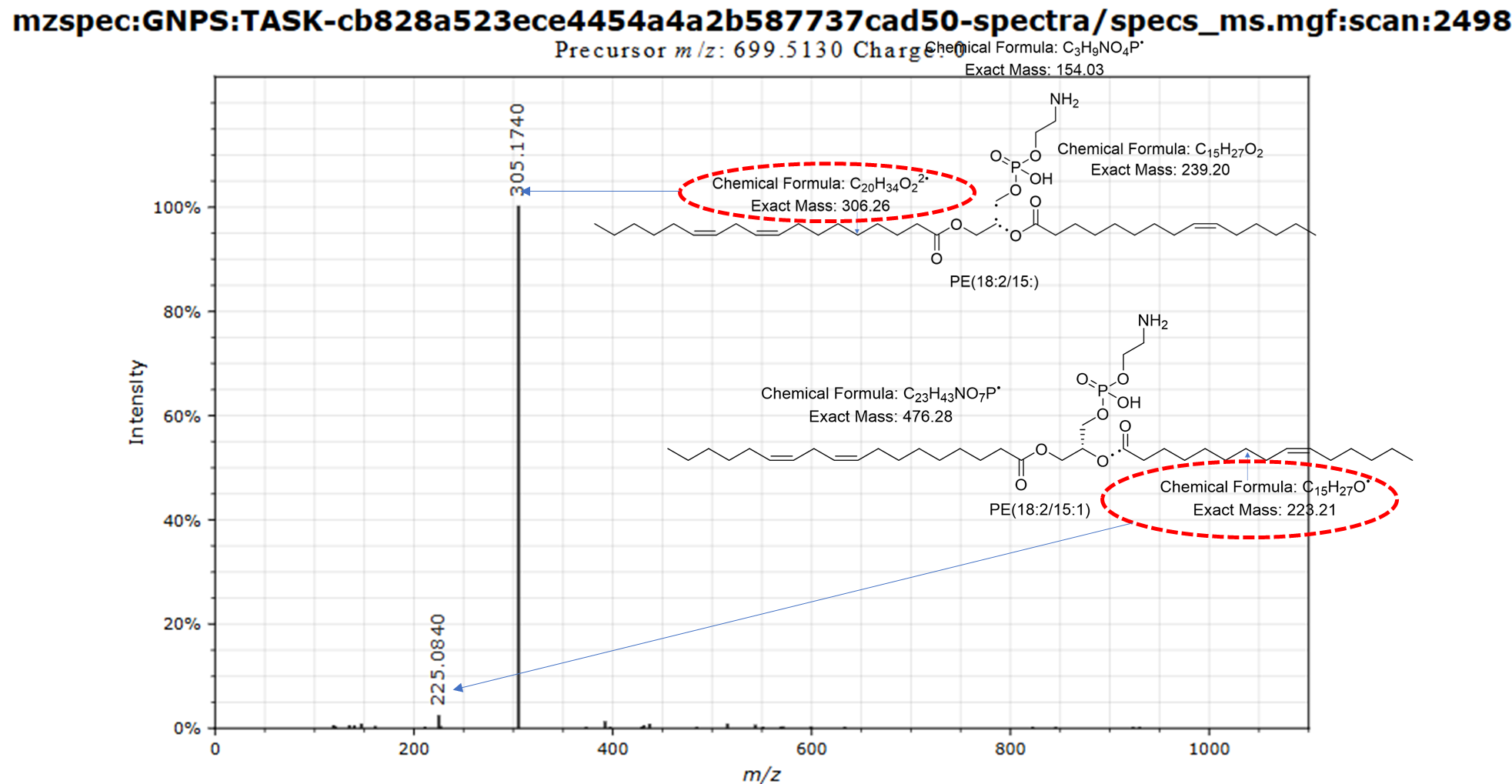


Supplementary Figure 45. LC-MS/MS of PE(18:2/15:1)


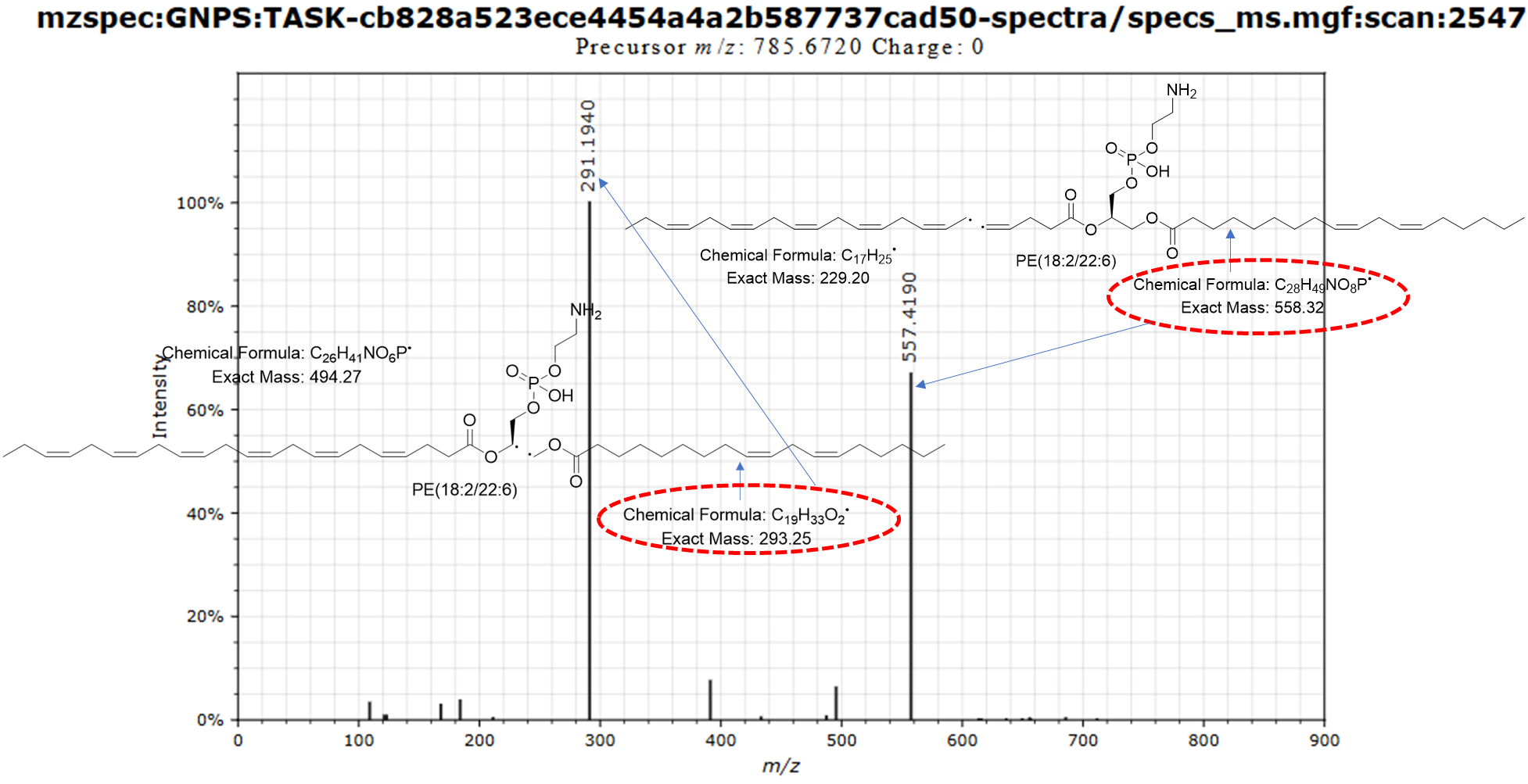


Supplementary Figure 46. LC-MS/MS of PE(18:2/22:6)


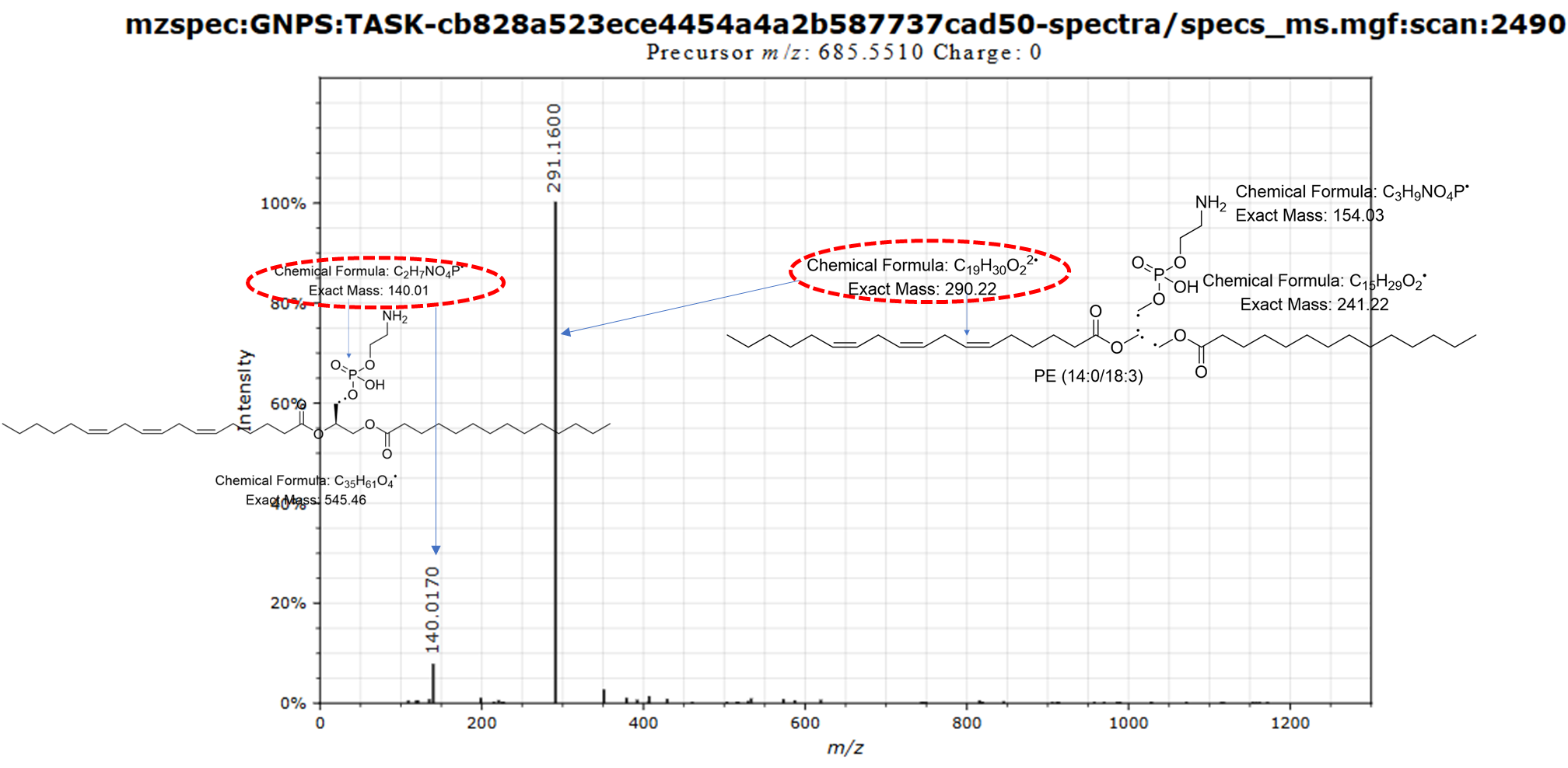


Supplementary Figure 47. LC-MS/MS of PE(14:0/18:3*)*


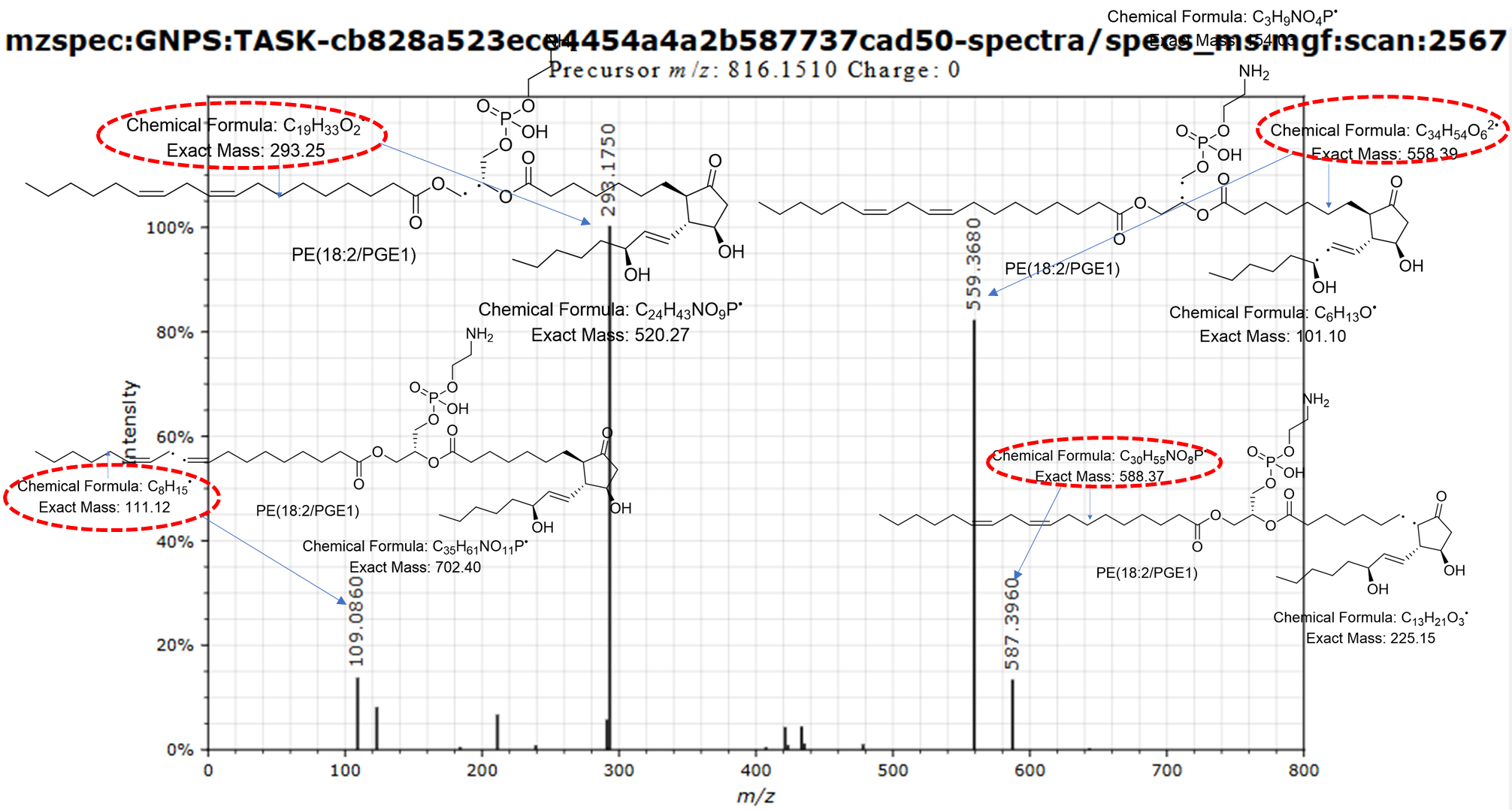


Supplementary Figure 48. LC-MS/MS of PE(18:2/PGE1)


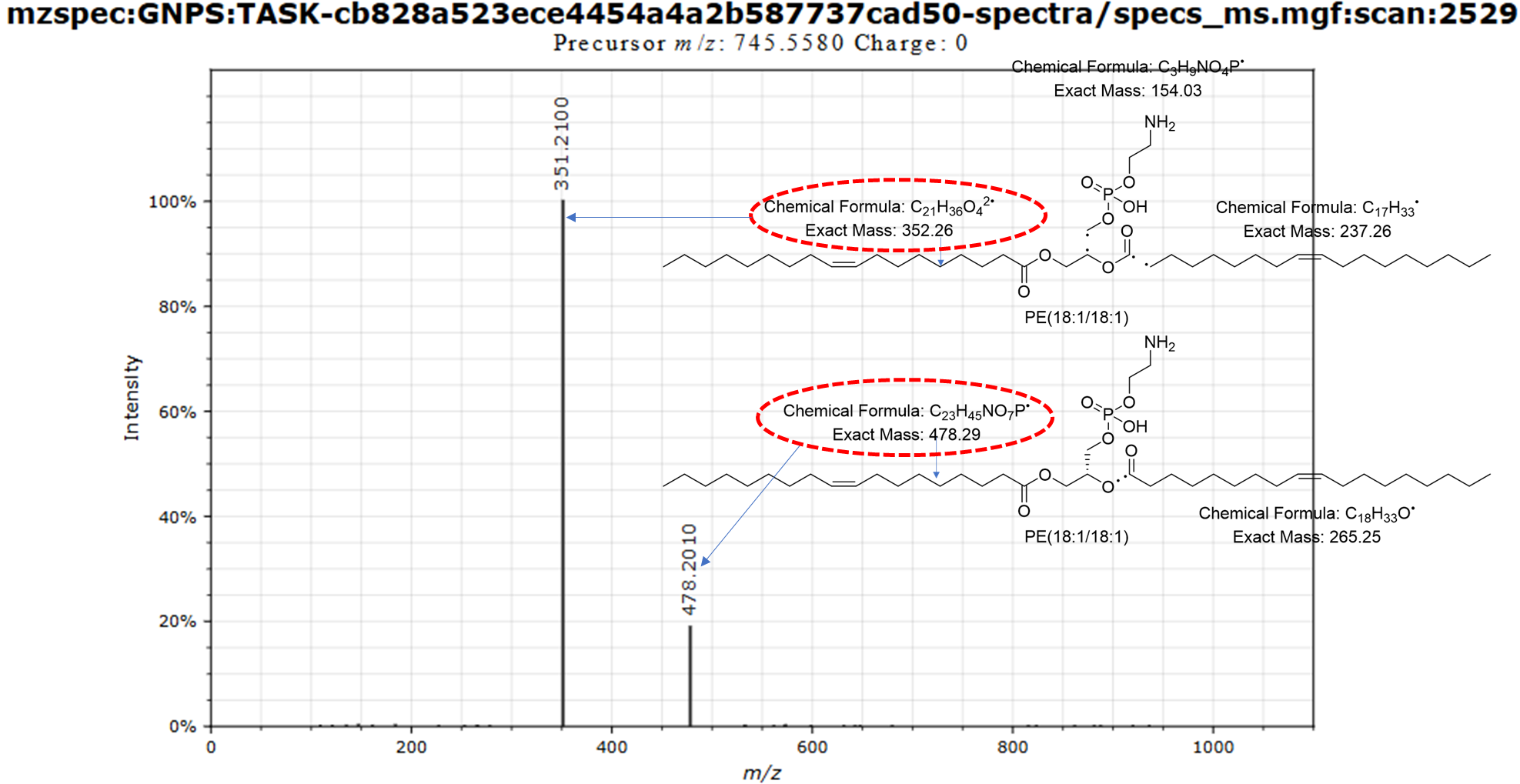


Supplementary Figure 49. LC-MS/MS of PE(18:1/18:1)


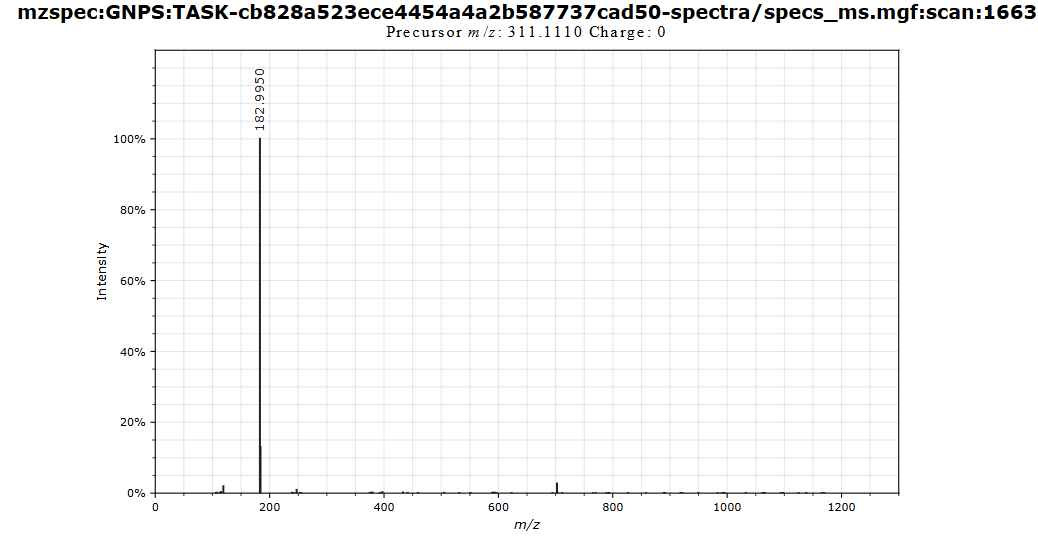


Supplementary Figure 50. LC-MS/MS of 7,10-DiHODE


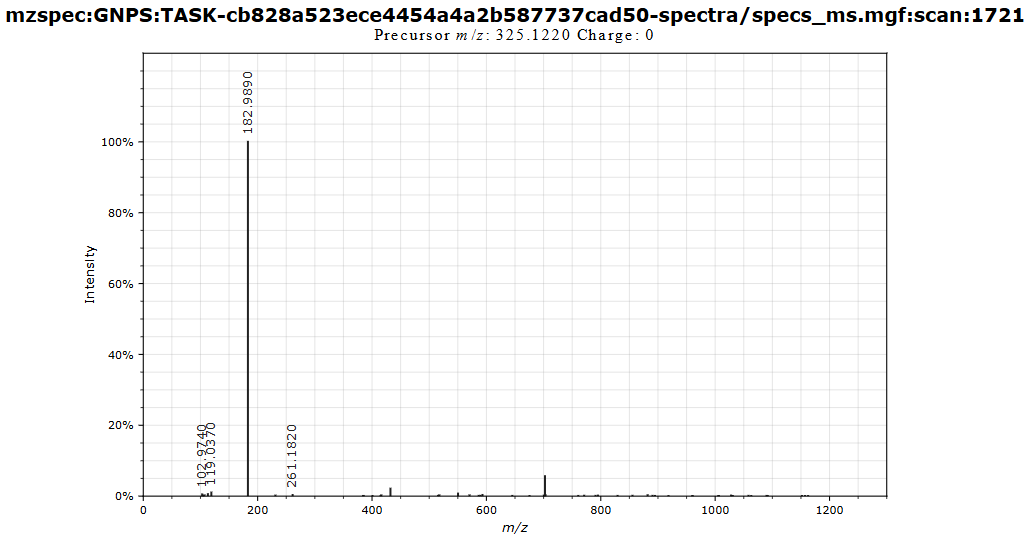


Supplementary Figure 51. LC/MS-MS of bilobalide


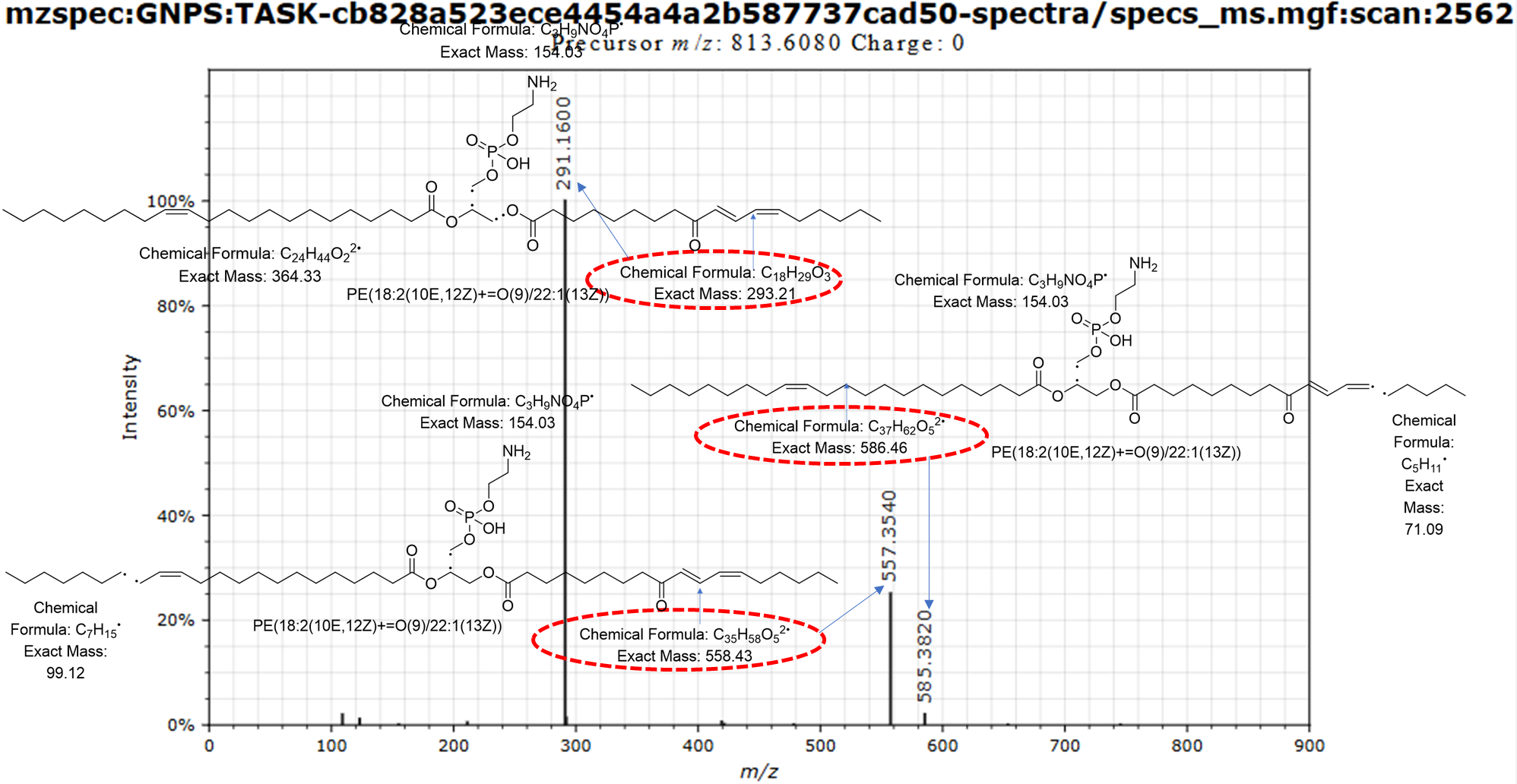


Supplementary Figure 52. LC-MS/MS of PE(18:2(10,12)+=O(9)/22:1(13))


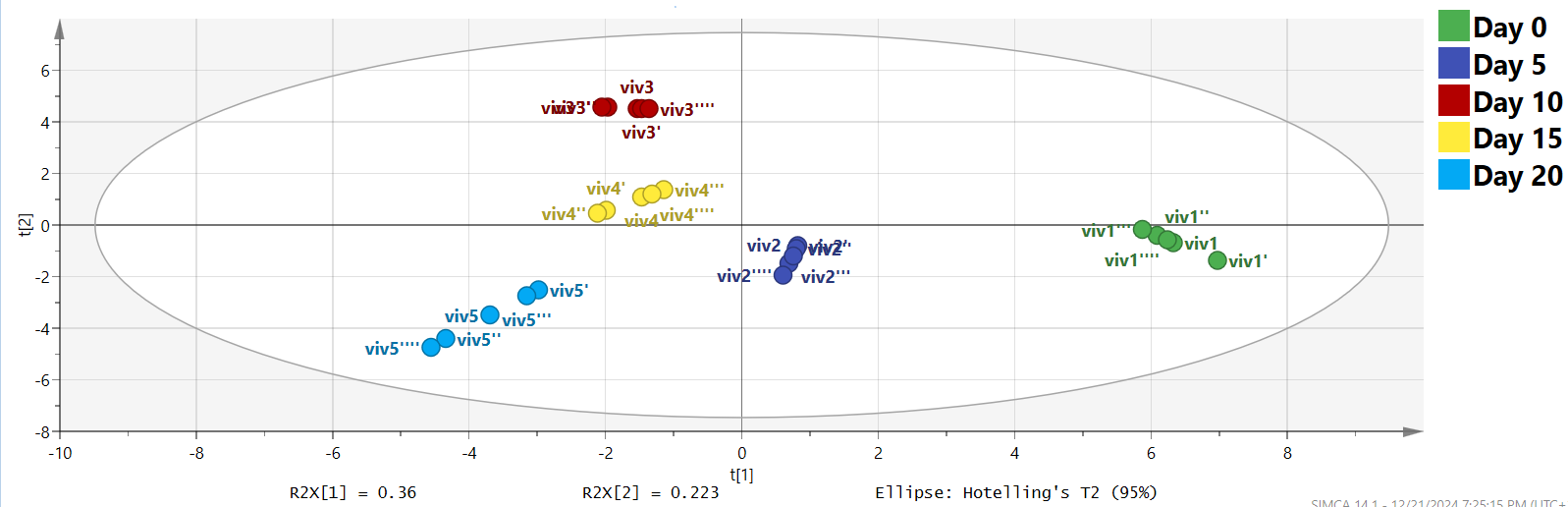


Supplementary Figure 53. PCA score scatter plot of the tested grape samples.
